# WNT10B/β-catenin signalling induces HMGA2 and proliferation in metastatic triple-negative breast cancer

**DOI:** 10.1002/emmm.201201320

**Published:** 2013-01-11

**Authors:** Peter Wend, Stephanie Runke, Korinna Wend, Brenda Anchondo, Maria Yesayan, Meghan Jardon, Natalie Hardie, Christoph Loddenkemper, Ilya Ulasov, Maciej S Lesniak, Rebecca Wolsky, Laurent A Bentolila, Stephen G Grant, David Elashoff, Stephan Lehr, Jean J Latimer, Shikha Bose, Husain Sattar, Susan A Krum, Gustavo A Miranda-Carboni

**Affiliations:** 1 Department of Obstetrics and Gynecology, David Geffen School of Medicine at UCLA, Jonsson Comprehensive Cancer CenterLos Angeles, CA, USA; 2 UCLA and Orthopaedic Hospital Department of Orthopaedic Surgery and the Orthopaedic Hospital Research Center, David Geffen School of Medicine at UCLALos Angeles, CA, USA; 3 Institute of Pathology, Charité University Medicine/UKBFBerlin, Germany; 4 Department of Brain Tumor Biology, The University of ChicagoChicago, IL, USA; 5 Department of Pathology, The University of ChicagoChicago, IL, USA; 6 California NanoSystems Institute and Department of Chemistry and Biochemistry, University of CaliforniaLos Angeles, CA, USA; 7 Department of Pharmaceutical Sciences, Nova Southeastern University, Fort Lauderdale-DavieFL, USA; 8 Department of Biostatistics, UCLA School of Public Health, David Geffen School of Medicine at UCLALos Angeles, CA, USA; 9 Baxter Innovations GmbHVienna, Austria; 10 Department of Pathology and Laboratory Medicine, David Geffen School of Medicine at UCLALos Angeles, CA, USA; 11 Department of Pathology and Laboratory Medicine, Cedars Sinai Medical CenterLos Angeles, CA, USA

**Keywords:** cancer stem cells, HMGA2, metastasis, triple negative breast cancer, wnt signalling

## Abstract

Wnt/β-catenin signalling has been suggested to be active in basal-like breast cancer. However, in highly aggressive metastatic triple-negative breast cancers (TNBC) the role of β-catenin and the underlying mechanism(s) for the aggressiveness of TNBC remain unknown. We illustrate that WNT10B induces transcriptionally active β-catenin in human TNBC and predicts survival-outcome of patients with both TNBC and basal-like tumours. We provide evidence that transgenic murine *Wnt10b*-driven tumours are devoid of ERα, PR and HER2 expression and can model human TNBC. Importantly, HMGA2 is specifically expressed during early stages of embryonic mammogenesis and absent when WNT10B expression is lost, suggesting a developmentally conserved mode of action. Mechanistically, ChIP analysis uncovered that WNT10B activates canonical β-catenin signalling leading to up-regulation of HMGA2. Treatment of mouse and human triple-negative tumour cells with two Wnt/β-catenin pathway modulators or siRNA to HMGA2 decreases HMGA2 levels and proliferation. We demonstrate that WNT10B has epistatic activity on HMGA2, which is necessary and sufficient for proliferation of TNBC cells. Furthermore, HMGA2 expression predicts relapse-free-survival and metastasis in TNBC patients.

## INTRODUCTION

In 2011, over 230,000 new cases of invasive breast cancer were diagnosed in the United States and consequently breast cancer is the second leading cause of death in women. These breast cancers are divided into 6–10 subtypes that are highly heterogeneous both histologically (Weigelt & Reis-Filho, 2009) and by gene expression profiling (Banerji et al, 2012; Perou et al, 2000; Sorlie et al, 2003): luminal A, luminal B, ERBB2/HER2, normal breast-like, basal-like and triple-negative (TN). Targeted therapies for certain of these subtypes exist: luminal A breast cancers are oestrogen receptor alpha (ER) positive and are treated with ER pathway inhibitors, such as tamoxifen or aromatase inhibitors. ERBB2/HER2/neu (HER2) positive tumours are treated with the monoclonal antibody herceptin. The triple-negative breast cancer (TNBC) subtype [*i.e.* lacking expression (or overexpression) of ER and HER2, as well as progesterone receptor (PR)] is specifically associated with a poor prognosis and a highly metastatic phenotype. Currently, treatment options for TNBC are limited to cytotoxic chemotherapy (Pal et al, 2011). TNBC is more prevalent among premenopausal women of African American ancestry (39%) than in non-African American women (16%; Carey et al, 2006). Survival disadvantage is seen in TNBC when compared to other cancer subtypes regardless of stage at diagnosis, with a 5-year survival of only 77% compared with 93% for other breast cancers. Much work is still needed to develop targeted neoadjuvant therapies for the TNBC subtype.

The *Wnt/β-catenin* pathway has been shown to be activated in basal-like tumours (Khramtsov et al, 2010). *Wnt*/β-catenin signalling is activated by interaction of Wnt ligands with its receptors subsequently leading to the stabilization of β-catenin. Stabilized β-catenin translocates to the nucleus and induces specific transcriptional programs influencing cellular responses including, but not limited to, cellular proliferation, development, differentiation, neoplasia and stem cell maintenance (Moon et al, 2004; Nusse, 2005; Wend et al, 2010).

*Wnt10b* plays a very fundamental role during mammary gland development, as it is the earliest discernible ectodermal event (E11.25) defining the mammary gland ridge. It is expressed in the mammary anlagen and is characteristic for the definitive mammary lineage (Veltmaat et al, 2004). Elevated expression of *Wnt10b* results in mammary tumorigenesis in mice, and has been detected in human breast carcinoma cell lines (Wend et al, 2011). In order to model human breast tumorigenesis several *Wnt*/β-catenin pathway-related mouse models have been generated, and it is believed that tumours originate from stem cells and/or progenitor cells (*e.g. MMTV-Wnt1*, *MMTV*-*Wnt10b* and *MMTV-ΔN-*β*-catenin* amongst others; Wend et al, 2011).

Although nuclear β-catenin is upregulated in more than 50% of breast cancer cases (Cowin et al, 2005), mutations in genes encoding intracellular signalling components are rare. This may suggest deregulation at the cell surface to be a possible key mechanism to explain high levels of β-catenin in breast cancer. It is well established that signalling components that function transiently during embryonic development might become an oncogene by constitutively re-activating embryonic signalling programs in adult tissue(s) (Polyak & Weinberg, 2009). In mammary gland development and breast tumorigenesis an exclusive *Wnt* pathway component to be considered is *Wnt10b*. However, the knowledge about the functional and mechanistic consequences of Wnt/β-catenin signalling in mammary progenitor cells and breast oncogenesis is still limited. We have recently shown that mechanistically *Wnt10b*-driven (*Wnt10b*^*TG*^) tumours degrade the tumour suppressor p27^Kip1^ in a SKP2-independent manner mediated by CUL4A E3-ligase activity leading to increased proliferation (Miranda-Carboni et al, 2008).

In this study we have identified HMGA2 as the most highly expressed gene in *Wnt10b*-driven tumours. The HMGA protein family includes HMGA1a and HMGA1c, which are encoded by the same gene, and the closely related HMGA2, which is known to be over-expressed in breast cancer (Peluso & Chiappetta, 2010). HMGA2 is expressed in breast tumours of high histological grade (3), but not in those of lower grades (Rogalla et al, 1997). HMGA family members play important roles in stem cell self-renewal, proliferation and differentiation (Fusco & Fedele, 2007). They are widely expressed during early embryogenesis but are restricted as foetal development progresses; they are not expressed in normal adult tissue (Fusco & Fedele, 2007; Zhou et al, 1995). HMGA2 associates with chromatin and regulates transcription by altering chromatin structure but itself does not bind DNA (Reeves, 2000). HMGA2 directly regulates CCNA2, CCNB2 and physical phospho-RB/E2F1 interactions (reviewed in Fusco & Fedele, 2007).

In the present study, we provide evidence that *Wnt10b*-driven tumours are devoid of ERα, PR and HER2 protein expression and can be used as a translational model for human TNBC. We show that both WNT10B and HMGA2 are highly expressed in a subset of human primary TNBC tumours correlating with predictive poor survival outcome in both basal-like and TNBC patients. From the mouse model, we are able to translate WNT10B, HMGA2 and proliferation markers (amongst others) to human TNBC cell lines and primary TNBC samples. Treatment of TNBC-derived cell lines and primary tumour mouse cells with known *Wnt* pathway inhibitors down regulates expression of HMGA2 and cell proliferation markers. WNT10B has an epistatic effect on HMGA2, and HMGA2 is essential and necessary for proliferation in both mouse and human TN cell lines. More importantly, the regulation of HMGA2 by WNT10B is developmentally conserved in the embryonic mouse mammary gland anlagen. The translational *Wnt10b* model may provide a novel therapeutic tool to develop inhibitors to control *Wnt/β-catenin*-mediated proliferation in human TNBC.

## RESULTS

### Human triple-negative breast cancers (TNBC) express WNT10B, show active Wnt signalling, and have high proliferation, demonstrating that WNT10B has clinical relevance and prognostic value

Compelling evidence suggests that WNT10B may be a valuable candidate for the development of therapeutic regimens for certain human diseases [reviewed; (Wend et al, 2011)]. The *Wnt10b*-driven tumour mouse model has been shown to be useful to model human breast cancer (Miranda-Carboni et al, 2008), but still lacking is direct evidence that WNT10B can be linked to human primary TNBC patients. Thus we questioned: (i) Is WNT10B expression detectable in primary TNBC samples? (ii) Can WNT10B expression activate canonical Wnt-signalling-mediated gene expression in primary TNBC? and (iii) Does WNT10B expression predict survival outcome? It is important to note that the molecular, clinical and pathological profiles of basal-like and TNBC are similar as they overlap by 60–90% (Al Tamimi et al, 2010); the terms are often interchangeable but they are not identical in their gene expression profiles leading to at least six to eight complex subtypes (Perou et al, 2000). In our study the term basal-like will be utilized when referencing microarray data (*i.e.* mRNA expression of ERα^−^, PR^−^ and HER2^−^) and the term TNBC will refer to analysis by a pathologist who subsequently classified the tumours as ERα^−^, PR^−^, HER2^−^ by immunohistochemistry (IHC). Triple-negative (TN) will be used to describe both basal-like and TNBC.

**Figure 1:**
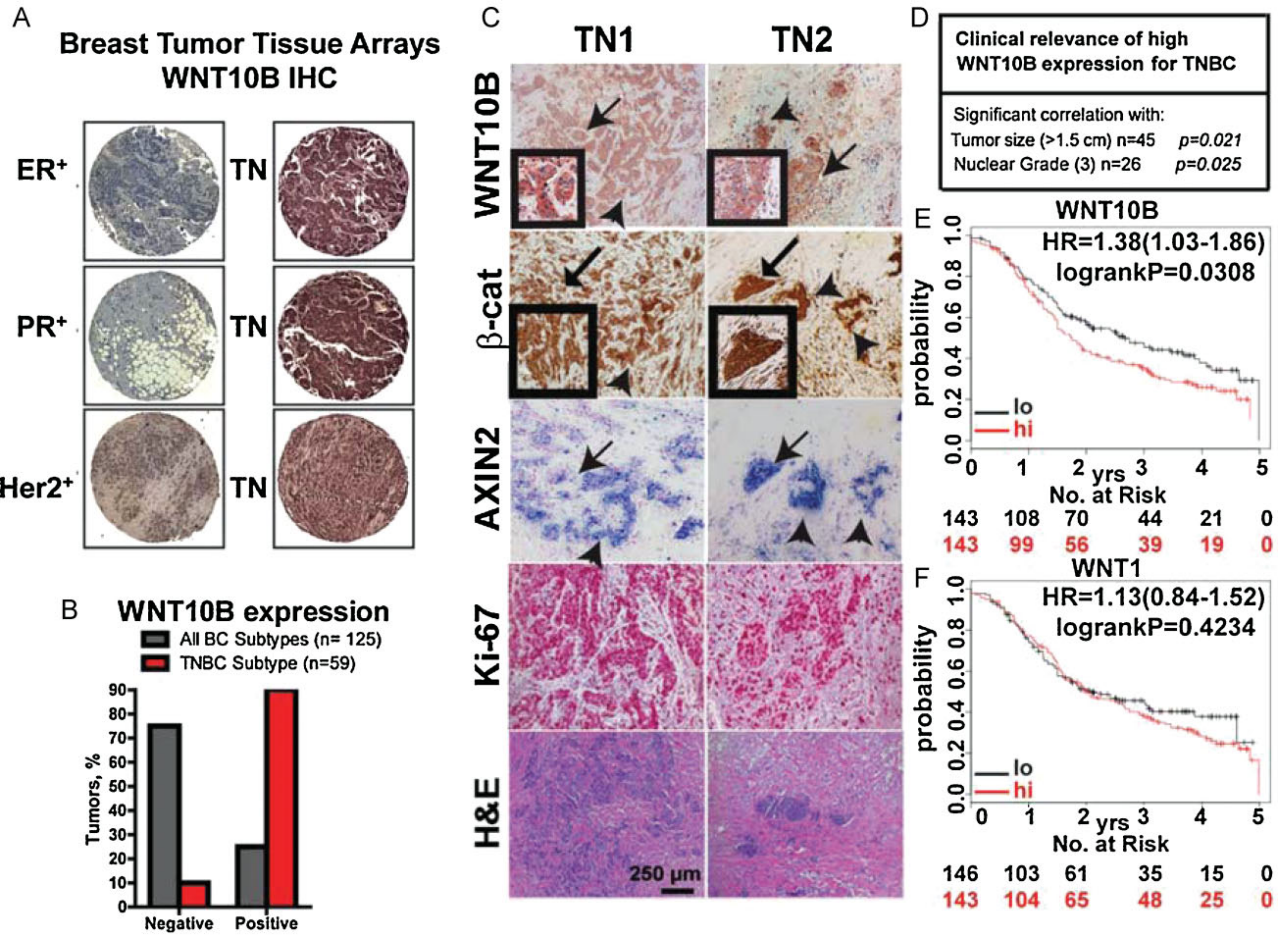
Human triple-negative breast cancers (TNBC) express WNT10B, show active Wnt signalling and have high proliferation, and WNT10B has clinical relevance and prognostic value **A, B.** Immunohistochemical (IHC) analysis of tissue microarrays of different breast cancer subtypes shows predominant WNT10B expression in TNBC (quantifications are shown in **B**).**C.** TNBC tumours were analysed by IHC for WNT10B, β-catenin and Ki-67 expression. AXIN2 (a Wnt/β-catenin target gene) mRNA levels were determined by *in situ* hybridization (ISH). Arrows indicate a similar localization for IHC and ISH. Ten times magnifications are shown. Inserts show 40× magnifications.**D.** High WNT10B expression in TNBC is significantly correlated with large tumour size and high nuclear grade (WNT10B expression was determined by IHC in TNBC patient samples and results were correlated with patient data). Associations between markers and clinical parameters that were measured on a continuous or ordinal scale were evaluated using Kendall's Tau coefficient. Associations between markers and binary clinical parameters were tested using an exact trend test, associations with nominal parameters were tested using Fisher's exact test. *p* Values and patient numbers (*n*) are indicated in the table. Additional information can be found in Supporting Information Fig S7 and the Materials and Methods Section.**E, F.** Kaplan–Meier survival analysis showing significantly improved recurrence-free survival of basal-like breast cancer patients with tumours that express lower levels of WNT10B (E) but not WNT1 (F). Patients with higher (red) and lower expression (black) are indicated as well as numbers of patients at risk at specific time points (below each diagram). Hazard ratios (HR) and *p* values (log rank *p*) are depicted for each survival analysis. Kaplan–Meier survival data were generated using the publicly accessible online tool KM-plotter. *p* Values of <0.05 were considered to be statistically significant (D–F).

To determine the expression of WNT10B in human primary breast cancer, we analysed commercially available tumour microarrays (TMA) consisting of 125 samples of all different subtypes by IHC (TNBC ∼15% of total: Ohio St. Univ. Human Tissue Bank). These 18 TNBC tumours on the TMA from the Ohio Tissue Bank Cohort were selected to be between the ages of 33 and 45 years old at the time of diagnosis. Expression of WNT10B is absent or low in ER^+^, PR^+^ and HER2^+^ tumours (Fig 1A). In contrast, most of the TNBC samples found in the TMA were very high for WNT10B expression. Additionally, we collected 59 TNBC from patients and our pathologist quantified and scored our samples as either positive or negative for WNT10B expression for this analysis (Fig 1B). Most of the samples in the TMA were negative (>75%) and a few were positive (<10%) for the presence of WNT10B protein. Conversely, most of the samples in our collection of 59 TNBC score positive (>80%) for WNT10B.

We next analysed primary TNBC samples from a German cohort (*n* = 14) for the presence of both WNT10B and β-CATENIN to verify if they overlapped (Fig 1C). In sequential tumour sections the expression of WNT10B and β-CATENIN correlate in similar regions (*i.e.* arrow and arrow-heads). More importantly, the same areas also show high expression of *AXIN2*, a well-known canonical Wnt direct-target (Lustig et al, 2002) demonstrated by *in situ* hybridization (ISH). The same tumours also had high expression of Ki67 (Fig 1C). We have validated the specificity of the WNT10B antibody utilizing transgenic *MMTV-Wnt10b-IRES-LacZ*-derived tumours and cell lines, human triple negative breast cancer cell lines and *Wnt10b*-knockout embryos (E.14.5; Supporting Information Fig S1A and B). We also contrasted the expression of both β-CATENIN [*i.e.* non-phospho (active) β-catenin (SER33/37/Thr41) *vs.* a pan-β-catenin-antibody] and AXIN2 in TNBC with other breast cancer subtypes (ER^+^, PR^+^, HER2^+^ and TP^+^; Supporting Information Fig S8A–C).

The hallmark of clinical relevance in cancer biology is to evaluate the association of a given gene expression with survival outcome. To this end we utilized the available clinical data from our collection of 59 TNBC samples, and identified two clinical parameters that exhibit significant correlations with high WNT10B expression (Fig 1D): (i) big tumour size >1.5 cm (*n* = 45, *τ* = 0.28, *p* = 0.021), and (ii) high nuclear grade status 3 (*n* = 26, *τ* = 0.420, *p* = 0.025). Pathologists associate these two characteristics for a given tumour to have an overall poorer outcome.

Concurrently, we employed a publicly available database and Kaplan–Meier software (KM-plotter) that has the ability to assess 22,277 genes in over ∼3000 breast cancer samples [http://kmplot.com/breast/; (Gyorffy et al, 2010)]. We utilized the basal-like database and identified >280 patients that were screened for both *WNT10B* and *WNT1* expression. Recurrence-free survival was depicted using the Kaplan–Meier survival estimates with a medium cutoff for *WNT10B* and *WNT1* expression (Fig 1E–F). Remarkably, survival outcome differed between patients with low and high WNT10B expression values. Cox proportional hazards regression revealed an increased risk in patients with high WNT10B expression (HR = 1.38, *p* = 0.03). In contrast, *WNT1* expression was unable to predict survival outcome (HR = 1.13, *p* = 0.42). Additionally, *WNT10B* expression could not predict survival outcome of patients of any other breast cancer subtype tested with the KM-plotter.

In summary, these results illustrate for the first time that *WNT10B* expression has significant clinical relevance and probability for predicting recurrence-free survival in breast cancer for both basal-like and TNBC tumours. Our hypothesis that WNT10B would activate canonical Wnt-signalling and activate gene expression was validated with *AXIN2* expression in our TNBC patient samples.

### The Wnt10b^LacZ^ mammary tumours are triple-negative

**Figure 2:**
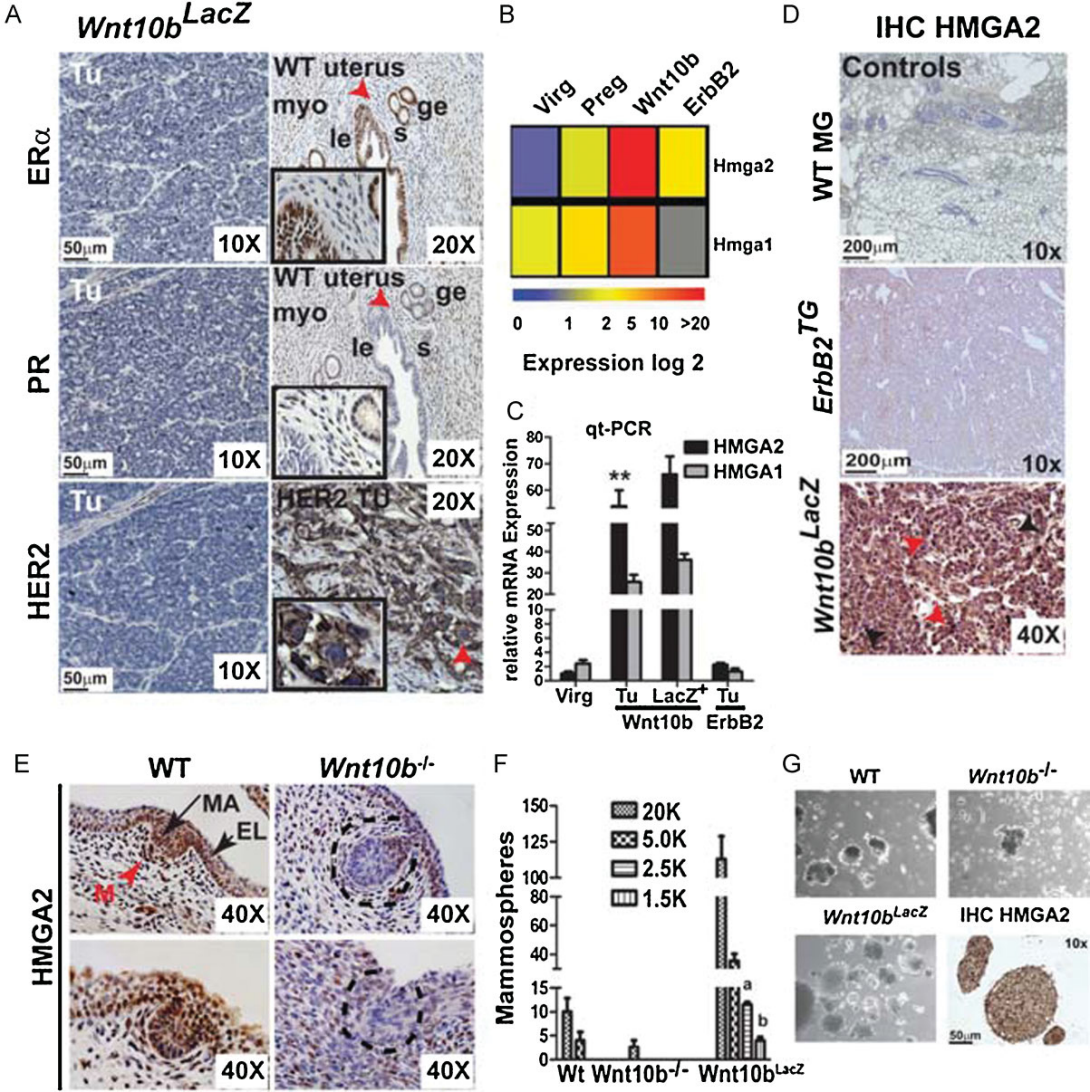
The self-renewal marker HMGA2 is highly expressed in *Wnt10b^TG^* mammary tumours and mammary placodes **A.** IHC for oestrogen receptor α (ERα), progesterone receptor (PR) and Her2 to validate the triple-negative phenotype of *Wnt10b*^*LacZ*^ tumours. Wild-type (WT) uterus and Her2 tumour samples were used as positive controls. Highlighted are myoepithelial (myo), glandular epithelial (ge), luminal epithelial (le) and stromal cells (s).**B.** Hierarchical clustering of microarray data of *Hmga2* and *Hmga1* expression in *Wnt10b*-driven tumours.**C.** Validation of *Hmga2* and *Hmga1* expression in tumour cells (Tu), Lin^−^LacZ^+^ (LacZ^+^) tumour cells compared to wild-type virgin (Virg) and *ErbB2*^*TG*^ tumour cells, as determined by qt-PCR. Error bars represent the means and the standard deviations from three independent experiments; **p-value = 0.007 *versus* Virg or *ErbB2*^*TG*^ Tu samples (Student's *t*-test).**D.** IHC of HMGA2 in WT mammary glands, and *ErbB2*^*TG*^ and *Wnt10b*^*LacZ*^ tumours. Red arrowheads highlight cells positive for HMGA2 and black arrowheads negative cells, respectively.**E.** IHC of HMGA2 in WT and *Wnt10b knockout (Wnt10b*^*−/−*^) mammary placodes (mp) at embryonic Day 14.5 reveals loss of HMGA2 expression in *Wnt10b*^*−/−*^ mp. Dashed black lines indicate *Wnt10b*^*−/−*^ mp. Mesenchymal (M: red arrow head), epidermis layer (EL: short black arrow) and mammary anlagen (MA: long black arrow).**F, G.** Mammosphere assays (MSA) of WT and *Wnt10b*^*−/−*^ mammary gland cells and *MMTV-Wnt10b*^*LacZ*^ primary tumour cells that were serially passaged (at Day 10 and 20) and analysed at Day 30. Secondary spheres were counted in sequential dilutions and imaged using a phase contrast microscope. Biological replicates (*n* = 3) and six technical replicates are shown. *Wnt10b*^*LacZ*^-derived MSAs analysed by IHC for HMGA2 is shown (10×) in lower right panel of G (bar, 50 µm). In F error bars represent the means and the standard deviations from three independent experiments; *p* values: *a* = 0.04, *b* = 0.03 *versus* wild-type or *Wnt10b*^*−/−*^ samples (Student's *t*-test). *p* Values of <0.05 were considered to be statistically significant (C, F).

To determine how well our novel *MMTV-Wnt10b-IRES-LacZ* (*Wnt10b*^*Lac*Z^) mouse model resembles human TNBC, we conducted IHC for estrogen receptor-alpha (ERα), progesterone receptor (PR) and HER2 protein expression levels (Fig 2A). *Wnt10b*-driven tumours are phenotypically like human TNBC—devoid of ERα, PR and HER2. In contrast, ERα and PR protein expression, in the uterus of wild-type female mice, is present in various subpopulations of myoepithelial, stromal cells and both luminal and glandular epithelial cells (Fig 2A). Interestingly, the *Wnt10b* tumorigenic mouse model contrasts the *MMTV-Wnt1* model, in part, because *Wnt1*-driven tumours have been reported to express hormone receptors (Teissedre et al, 2009). HER2 protein expression is very high in mouse *MMTV-neu2*/*ErbB2* (*ErbB2*^*TG*^) tumours but not expressed at all in the *Wnt10b*^*Lac*Z^ tumours. Furthermore, *Wnt10b*-driven tumours express the basal-epithelial markers CK5 and CK6, in contrast to *ErbB2*^*TG*^ tumours (Supporting Information Fig S2A–C). We also show that *Wnt10b*^*Lac*Z^-driven tumours express high levels of β-galactosidase activity, have transcriptionally active nuclear β-catenin and correlating with high levels of AXIN2 protein expression (Supporting Information Fig S2D–F).

### HMGA2 is highly expressed in Wnt10b^TG^ mammary tumours and lacking in embryo mammary placodes of Wnt10b-knockout mice

In search for novel gene networks that are activated during early stages of development, repressed in adult tissue and reactivated during tumorigenesis, we identified a substantial number of candidates within our tumour microarray data (Miranda-Carboni et al, 2008). Of those candidates, high mobility group A family member 2 (*Hmga2*) was the highest regulated gene [125-fold increased expression over wild-type cells (*p* < 10^−9^); Fig 2B]. Furthermore, the related family member *Hmga1* was found to be up-regulated by 10-fold. We next validated *Hmga2* and *Hmga1* expression by qt-PCR (Fig 2C). There is twice as much *Hmga2* expression in both primary tumour cells and (Lin^−^) LacZ^+^ FACS-sorted tumour cells, than the *Hmga1* expression profile in the same cells. Neither *Hmga1* nor *Hmga2* were found to be expressed in virgin wild-type mammary tissue or *ErbB2*^*TG*^ tumours analysed by qt-PCR (Fig 2B–C). We next validated the expression of HMGA2 by IHC in *Wnt10b*^*LacZ*^-driven tumours (Fig 2D). There is strong expression of HMGA2 protein levels in most of the *Wnt10b*^*LacZ*^-driven tumours cells. In contrast, both virgin wild-type mammary gland tissue and *ErbB2*^*TG*^ tumour samples did not express any detectable HMGA2 protein, as would be expected.

We next determined if the *Wnt10b*-induced expression of *Hmga2* is embryologically conserved during early mammogenesis. To this end we analysed embryonic tissues (E14.5) from both wild-type and *Wnt10b*-knockout mice (*Wnt10b*^*−/−*^) for the presence of HMGA2 by IHC (Fig 2E). There is specific expression of HMGA2 in mammary anlagen epithelial cells (MA), as well as the epidermis layer (EL) and mesenchymal (M) cells of wild-type mice. In contrast, HMGA2 levels are strongly decreased throughout the three subtypes of tissues, particularly, in the mammary anlagen of the *Wnt10b*^*−/−*^ mice. Furthermore, this time point is the identical stage in which endogenous *Wnt10b* is high during early mammogenesis (Veltmaat et al, 2004).

Based on the above, we hypothesized that the loss of the self-renewal marker HMGA2 would have a functional consequence in adult *Wnt10b*^*−/−*^ mammary cells to generate mammospheres. Mammary gland cells were isolated from wild-type virgin and *Wnt10b*^*−/−*^ mice as well as from primary *Wnt10b*^*LacZ*^ tumours. Cells were grown under mammosphere assay (MSA) culturing conditions and sequentially passaged at 10 days and at 20 days generating tertiary-mammospheres, in a series of dilutions, followed by the assessment of MSA numbers at 30 days (Fig 2F). *Wnt10b*^*LacZ*^ tumours have approximately one self-renewing stem cell per 200 cells. In contrast, wild-type mammary cells were unable to generate MSA at the same frequency and the cells from *Wnt10b*^*−/−*^ mice do not have the capability of self-renewal (Fig 2F). Finally, mammospheres from time-point 30 days show high expression of HMGA2 (Fig 2G).

In summary, *Wnt10b*^*LacZ*^-driven tumours are devoid of hormone receptors and HER2 expression, with specific expression of HMGA2 in tumour tissue only. More importantly, we also provide evidence that HMGA2 is regulated by *Wnt10b*-expression in a developing embryo (E.14.5 early mammogenesis) and in the absence of WNT10B expression HMGA2 protein levels are diminished or completely lost in multiple tissues.

### Wnt10b/β-catenin signalling directly regulates HMGA2 expression in mouse mammary cell lines and regulates proliferation in mammary tumour cell lines

Based on the preceding experiments, we hypothesized that one potential function of HMGA2 in *Wnt10b*-driven tumours is the control of proliferation. HMGA2 is known to directly regulate the cell cycle proteins CCNA2 and CCNB2, and Rb/E2F1 protein–protein interactions (amongst other targets; De Martino et al, 2009; Fedele et al, 2006; Tessari et al, 2003). These cell proliferation markers (amongst others) are also upregulated in *Wnt10b*-driven tumours, as shown by microarray gene expression analysis and verifying qt-PCR (Supporting Information Fig S3A and B). *Wnt10b*-driven tumours, when compared to *ErbB2*^*TG*^-driven tumours, have an increased expression pattern for G1-, S- and G2-phase Cyclins.

**Figure 3:**
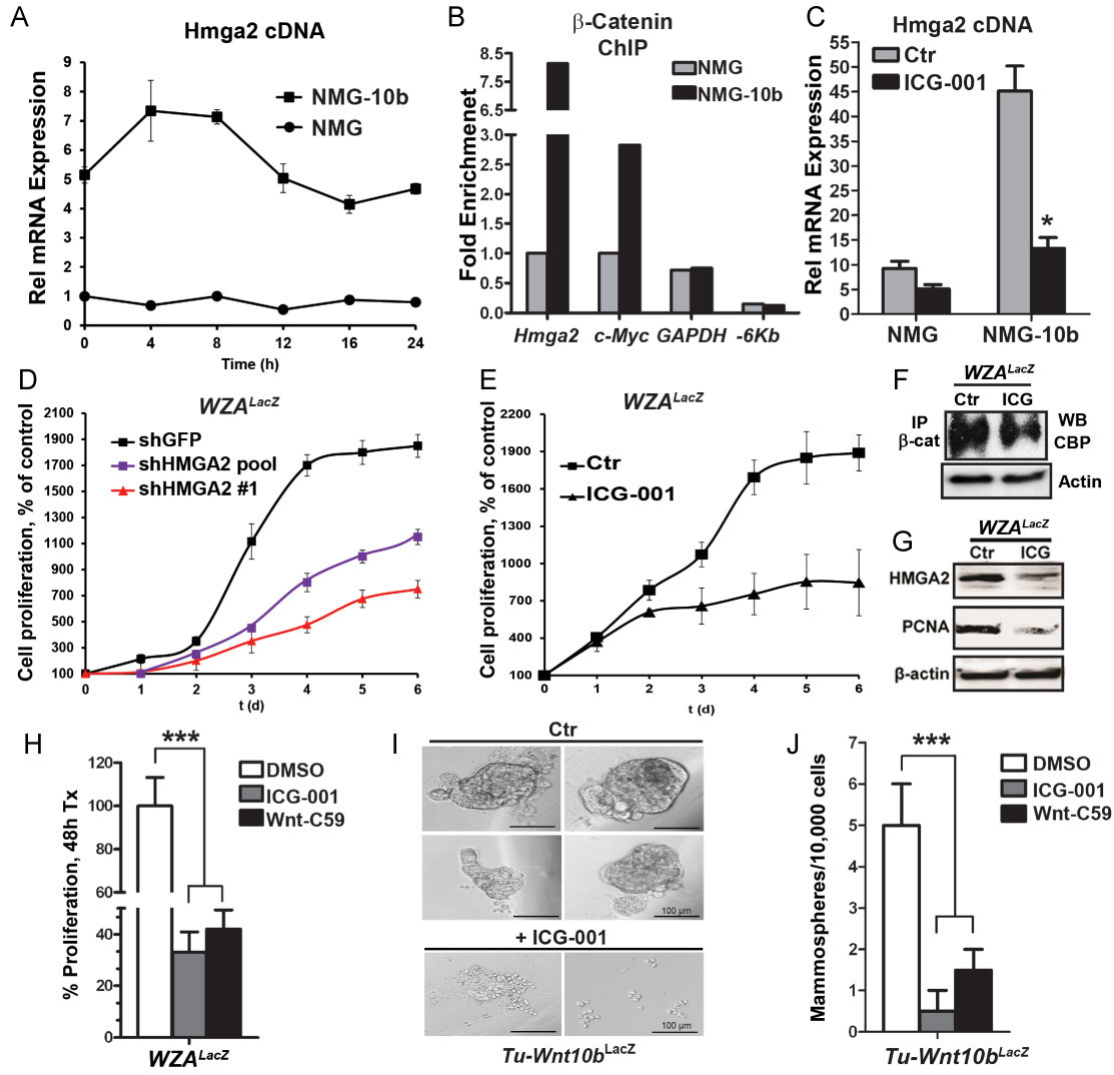
*Wnt10b* induces Hmga2-dependent proliferation via β-catenin in mouse mammary epithelial and tumour cells Time course experiment to analyse *Hmga2* expression in parental NMuMG (NMG) cells and NMuMG cells overexpressing Wnt10b (NMG-10b) after release from growth arrest (determined by qt-PCR). Error bars represent the means and the standard deviations from three independent experiments.Assessment of enrichment of β-catenin at the *Hmga2*, *c-Myc* and *Gapdh* promoters by chromatin immunoprecipitation (ChIP) 8 h after release from growth arrest. An upstream −6 Kb element of the *Hmga2* chromosomal ORF-loci devoid of WRE sites was used as negative control.Forty-eight hours treatment of NMG and NMG-10b cells with the Wnt/β-catenin inhibitor ICG-001 (10 µM in 1% DMSO) leads to decreased *Hmga2* expression, as determined by qt-PCR. Error bars represent the means and the standard deviations from three independent experiments; *p-value = 0.04 *versus* untreated sample (Student's *t*-test).shRNA-Mediated knockdown of *Hmga2* leads to attenuated growth of *WZA*^*LacZ*^ tumour cells. Shown are two different *shHmga2* clones compared to a control *shGFP* clone. Error bars represent the means and the standard deviations from three independent experiments.Attenuated proliferation of *WZA*^*LacZ*^ tumour cells upon treatment with ICG-001 (10 µM in 1% DMSO). Error bars represent the means and the standard deviations from three independent experiments.Immunoprecipitation and Western blot analysis verifying decreased interaction of β-catenin and CBP after ICG-001 treatment of *WZA*^*LacZ*^ tumour cells.Decreased expression of HMGA2 and PCNA in ICG-001-treated *WZA*^*LacZ*^ tumour cells, as determined by immunoblotting analysis.Quantification of proliferation of *WZA*^*LacZ*^ cells upon treatment with the Wnt pathway inhibitors ICG-001 or Wnt-C59 compared to vehicle control (DMSO). Error bars represent the means and the standard deviations from three independent experiments; ***p-value = 0.0008 (ANOVA).Images of MSA from primary *Wnt10b*^*LacZ*^ mammary tumour cells after control (DMSO, upper panel) or ICG-001 (lower panel) treatment.MSA formation efficiency of primary *Wnt10b*^*LacZ*^ mammary tumour cells after control (DMSO), ICG-001, and Wnt-C59 treatment. Error bars represent the means and the standard deviations from 12 independent experiments; ***p value = 0.0006 (ANOVA). *p* Values of <0.05 were considered to be statistically significant (C, H, J).

To gain mechanistic insights on how *Wnt10b* regulates HMGA2 expression we turned to our previously published mouse cell line system NMuMG (NMG) and NMuMG*-Wnt10b* (NMG-10b; Miranda-Carboni et al, 2008). NMG and NMG-10b cells were synchronized in early G_1_ by maintenance at 100% confluency for 2–3 days. Cells were then released and harvested at various times for analysis by qt-PCR. NMG-10b cell lines induce five- to eightfold greater *Hmga2* expression, at all-time points, than the control parental cell line NMG (Fig 3A). We also silenced *Hmga2* in the NMG-10b cell line utilizing a *Lentiviral-shHmga2* system to verify that the increased proliferation was due to HMGA2 expression. We observed decreased proliferation (>35%) after Hmga2 silencing, but not restored to NMG parental proliferation levels (Supporting Information Fig S3C).

To determine if the regulation of *Hmga2* expression by *Wnt10b* was indirect or mediated directly by β-CATENIN signalling loci, we utilized *in silico* analysis to identify potential TCF/LEF1-binding sites that could represent Wnt response elements (WRE) within the mouse *Hmga2* loci (http://genome.ucsc.edu/: for the genomic upstream sequences and http://www.gene-regulation.com/cgi-bin/pub/programs/patch/bin/patch.cgi: PATCH analysis for transcription binding sites).

To test the functionality of these putative *Lef1/Tcf4* sites, and to examine whether *Hmga2* represents a previously unrecognized *Wnt* target gene, chromatin immunoprecipitation (ChIP) analysis was performed with an antibody to β-catenin. NMG and NMG-Wnt10b cells were synchronized and released for 8 h. We chose 8 h after release from confluency because maximal *Hmga2* mRNA expression levels were obtained at that time point (Fig 3A). A this time point, β-catenin is enriched by eightfold at the *Hmga2* proximal promoter in Wnt10b-expressing cells but not in the parental cell line (Fig 3B). β-CATENIN is also enriched 2.5-fold at the promoter of a well-known *Wnt*-direct target gene, *c-Myc*, over the parental line (He et al, 1998). In contrast, β-CATENIN is not enriched at an upstream −6 Kb element of the *Hmga2* chromosomal ORF-loci devoid of WRE sites nor to the promoter of a negative control gene (GADPH) in either NMG or NMG-10b cell lines.

To further characterize if the expression of *Hmga2* depends on active β-catenin signalling we treated our cell lines with the *Wnt/β-catenin* signalling inhibitor ICG-001 (10 µM; Fig 3C). The inhibitor selectively inhibits TCF/β-catenin transcription in a CBP-dependent manner without interfering with β-catenin/p300 interactions (Emami et al, 2004). The data show that NMG-10b cells respond to ICG-001 treatment with decreased *Hmga2* expression close to the levels of the parental NMG cells. Together the results suggest that expression of *Hmga2* is dependent on β-catenin/CBP interactions mediated by *Wnt10b* signalling.

Based on the above experiments we hypothesized that if we silence *Hmga2* in a *Wnt10b*^*LacZ*^ tumour-derived cell line (*WZA*^*LacZ*^) we would block proliferation. To this end we generated stable clonal lines with lentivirally expressed short hairpin to *Hmga2* or the control *GFP* (Fig 3D). Two independent *shHmga2*-clones decreased proliferation by 38% (pool) and the other by 72% (#1) relative to control *shGFP*. Silencing of *Hmga2* and concurrent downregulation of *Ccna2* expression was verified by qt-PCR (Supporting Information Fig S3D and F). The results suggest that HMGA2 expression is necessary for maximal proliferation of *WZA*^*LacZ*^ cells.

We next wanted to verify if these tumour-derived cells, *WZA*^*LacZ*^, would respond to the *Wnt*-inhibitor ICG-001. To this end, we used non-synchronized randomly cycling cells in the presence or absence of ICG-001 (10 µM) and quantitated the effects on proliferation (Fig 3E). Consistent with our modelling, growth-inhibition was observed (>60%) after ICG-001 treatment. Furthermore, a second *Wnt10b*^*LacZ*^ cell line responded as well (Supporting Information Fig S3E). To confirm if the mechanism of action by ICG-001 is preserved in the mouse model, we conducted immunopercipitation (IP) experiments with β-CATENIN in the absence or presence of the inhibitor and immunoblotted for CBP (Fig 3F). ICG-001-treated cells lose the interactions between β-CATENIN and CBP. Moreover, we verified by immunoblotting that ICG-001 treatment resulted in decreased expression of HMGA2 and the proliferation marker PCNA (Fig 3G). We also wanted to determine if proliferation of *WZA*^*LacZ*^ cells would be dependent on *Wnt10b*-secretion and therefore treated the cells with Wnt-C59 (Cellagen Technology, Inc.), which blocks palmitylation of Wnt-proteins mediated by Porcupine, similar to Wnt-inhibitors IWP-2, -3 and -4 (Fig 3J). The data shows that Wnt-C59 is capable to significantly (*p* = 0.001) inhibit proliferation and comparable to the ICG-001 treated cells.

We next hypothesized that primary derived tumour cells that have a very potent *in vitro* ‘self-renewal’ capacity (Fig 2F and G) would respond to ICG-001 treatment. To do so, mammospheres were generated in low-adherence 96-well plates, as previously described from Lin^−^LacZ^+^ sorted cells, and the effects on mammosphere formation were measured in the presence or absence of ICG-0001 (10 µM; Fig 3I). ICG-001 completely blocked sphere formation. In parallel, we also wanted to determine if mammosphere formation from primary tumour cells would be dependent on *Wnt10b*-secretion and therefore treated the cells with Wnt-C59 (Fig 3J). Sphere formation was inhibited 10-fold by ICG-001 and threefold by Wnt-C59, respectively. The data suggest that both upstream *Wnt-*ligands, and downstream effectors of β-catenin signalling, are required for maintenance of sphere formation.

### Human TNBC cell lines express WNT10B and HMGA2 and are sensitive to β-catenin pathway inhibition

**Figure 4:**
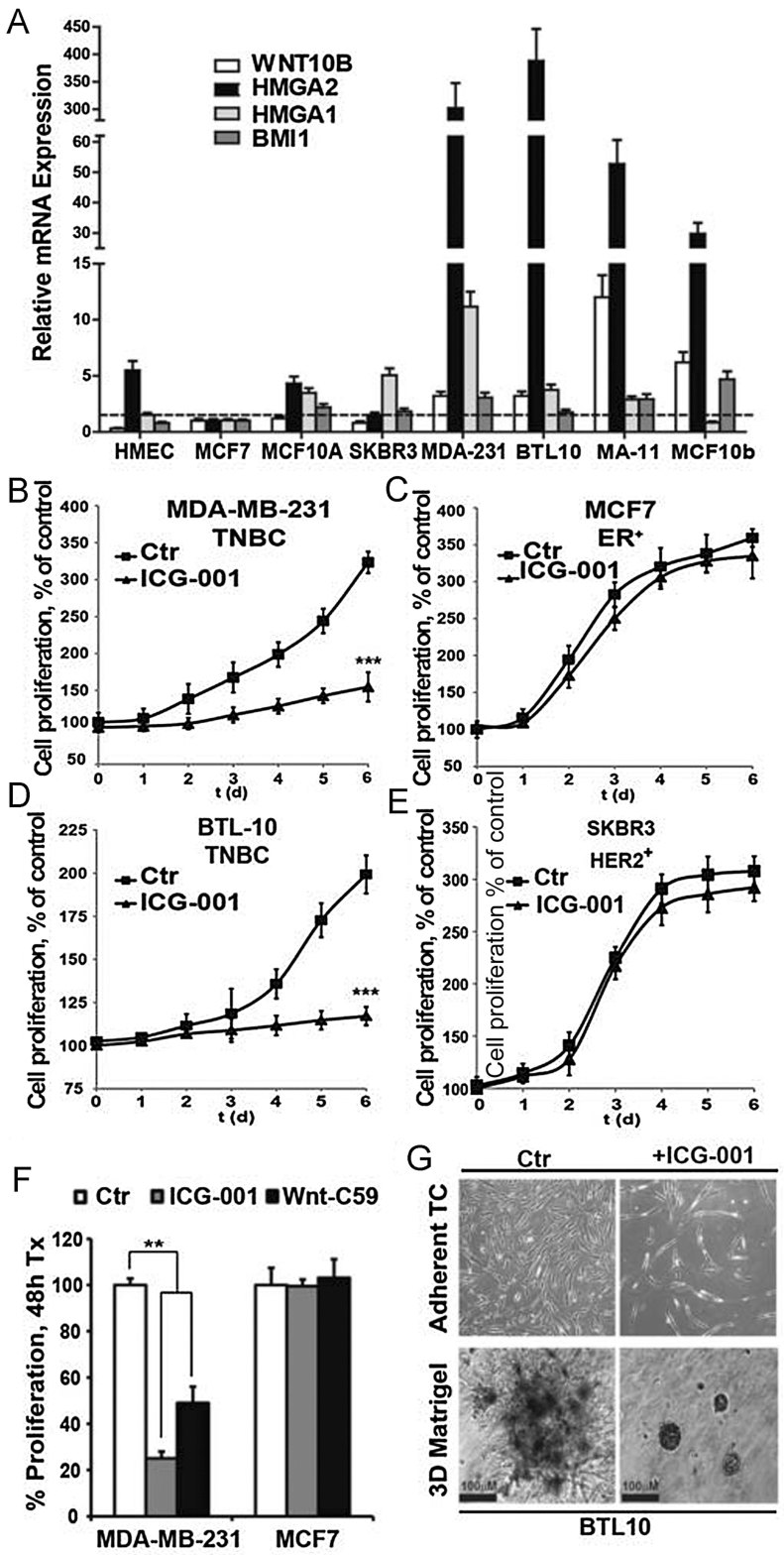
Triple-negative breast cancer (TNBC) cells in human express a specific gene signature and their self-renewal depends on canonical Wnt/β-catenin signalling **A.** Human TNBC cell lines (MDA-231, BTL10, MA-11) and other non-triple negative (TN) mammary cell lines tested by qt-PCR for expression of WNT10B and self-renewal markers. Relative mRNA levels are normalized to human MCF7 cells. Error bars represent the means and the standard deviations from three independent experiments.**B–E.** Proliferation of human TNBC cells (B, D) or human non-TN breast cancer cells (C, E) upon treatment with the Wnt/β-catenin inhibitor ICG-001 (10 µM in 1% DMSO). Error bars represent the means and the standard deviations from three independent experiments. In B, ***p value = 0.0005 *versus* control-treated cells (Student's *t*-test). In D, ***p value = 0.0007 *versus* control-treated cells (Student's *t*-test).**F.** Quantification of proliferation of human MDA-MB 231 and MCF7 cells upon treatment with the Wnt pathway inhibitors ICG-001 or Wnt-C59 compared to vehicle control (DMSO). Error bars represent the means and the standard deviations from three independent experiments; **p-value = 0.004 (ANOVA).**G.** Phase contrast images of BTL10 cells in adherent (upper panel) and mammosphere cell culture (lower panel) upon treatment with the Wnt/β-catenin inhibitor ICG-001 (10 µM in 1% DMSO, *n* = 3). *p* Values of <0.05 were considered to be statistically significant (B, D, F).

We next wanted to determine whether our identified gene signature is also expressed in human TNBC cell lines. We analysed by qt-PCR for the presence of *WNT10B*, *HMGA2*, *HMGA1* and *BMI-1* (as a control for self-renewal markers; amongst others) in a subset of human breast cancer cell lines: HMEC (human mammary epithelial cells; normal control), MCF7 (ER^+^ tumour-derived), MCF-10A (non-tumorigenic mammary epithelial), SK-BR-3 (HER2^+^ tumour-derived), MDA-MB-231 (mesenchymal stem-cell like (MSL)/TN), JL-BTL10 (BTL10 derived from TNBC stage 2B and grade 3 from a 38-year-old African-American women established by Jean Latimer), MA-11 (TNBC metastasising to bone), and MCF7-*Wnt10b* (MCF7-10b, a stable cell line expressing mouse *Wnt10b* in human ER^+^ tumour-derived cells (Miranda-Carboni et al, 2008; Fig 4A). *WNT10B* is most highly expressed in TN cell lines when compared to non-TN cell lines. Concurrently, both *HMGA2* and *HMGA1* are also most highly expressed in the TNBC cell lines and MCF7-10b cells have a high level of induction for *HMGA2* (>30-fold) and *BMI-1* (>5-fold) but not *HMGA1*. This may suggest that *HMGA2* and *BMI-1* are direct downstream targets of the WNT10B ligand. Moreover, HMGA2 is not responsive to 17β-estradiol (E2) treatment of parental MCF7 cells or in MCF7-10b cells; MCF7-10b cells are still responsive to E2 treatment by upregulation of *XBP1* and pS2 (Krum et al, 2008b), and express known canonical *Wnt*-signalling target genes, such as *c-myc*, *CCND1* and *DKK1* (Supporting Information Fig S4A; The Wnt homepage: Wnt targets, http://www.stanford.edu/group/nusselab/cgi-bin/wnt/). Importantly, treatment of MCF7-10b cells with ICG-001 blocks proliferation and down regulates HMGA2 expression (Supporting Information Fig S4B and C). MA-11 cells were originally isolated from a malignant bone marrow metastasis of a breast cancer patient and express high levels of both *WNT10B* and *HMGA2* (Micci et al, 2001). BTL10 cells are a novel TNBC cell line from an African-American patient that has been engineered to retain an undifferentiated state while in culture (Latimer et al, 2010). BTL10 cells show a high level of *HMGA2* induction (>400-fold), express *WNT10B* (threefold *vs.* HMEC control) and are *Wnt/β-catenin* responsive (Supporting Information Fig S4D).

We next hypothesized that inhibition of canonical Wnt-signalling would block proliferation in TN cell lines but not in cell lines derived from other breast cancer subtypes. For this purpose, non-synchronized randomly cycling cells were treated with the Wnt/β-catenin signalling inhibitor ICG-001 (10 µM) and the effects on proliferation were measured (Fig 4B–E). Both of the human TNBC cell lines (MDA-MB-231 and BTL10) had a >60% reduction in proliferation in the presence of ICG-001 (*p* = 0.001, Fig 4B and D). In contrast, proliferation of both MCF7 (ER^+^) and SKBR3 (HER2^+^) cells was not affected by ICG-001 treatment (Fig 4C and E). HMEC cells also did not respond to treatment (Supporting Information Fig S4E). We next wanted to determine if the inhibition of proliferation by ICG-001 on MDA-MB-231 was also dependent on *Wnt*-ligand secretion. To this end we treated the cells with Wnt-C59 inhibitor and measured the effects on proliferation (Fig 4F). Inhibition of *Porcupine* with Wnt-C59 blocked proliferation by >50% (*p* = 0.01). In contrast, in non-TN MCF7 control cells proliferation was not affected by either ICG-001 or Wnt-C59 treatment. To test the effects of ICG-001 on the capacity to block ‘self-renewal’, we generated mammospheres from BTL10 TNBC cell lines. We chose BTL10 because they have strong Wnt-reporter activity (Supporting Information Fig S4D) and retain an undifferentiated state while in culture resembling primary breast cancer explants. ICG-001 treatment of BTL10 cells robustly blocked mammosphere self-renewal in 3D-cultured cells and effectively stopped growth in 2D-cultured cells (Fig 4G).

In summary, we have illustrated that our mouse gene signature has been translated specifically to human TNBC cell lines and not to other subtypes of breast cancer cell lines. We also demonstrated that blocking of proliferation is dependent on both WNT-ligand secretion and β*-catenin* signalling.

### Inhibition of β-catenin signalling in human TNBC cell lines disrupts LEF-1, TCF4 and CBP protein–protein interaction that leads to direct repression of HMGA2 expression

**Figure 5:**
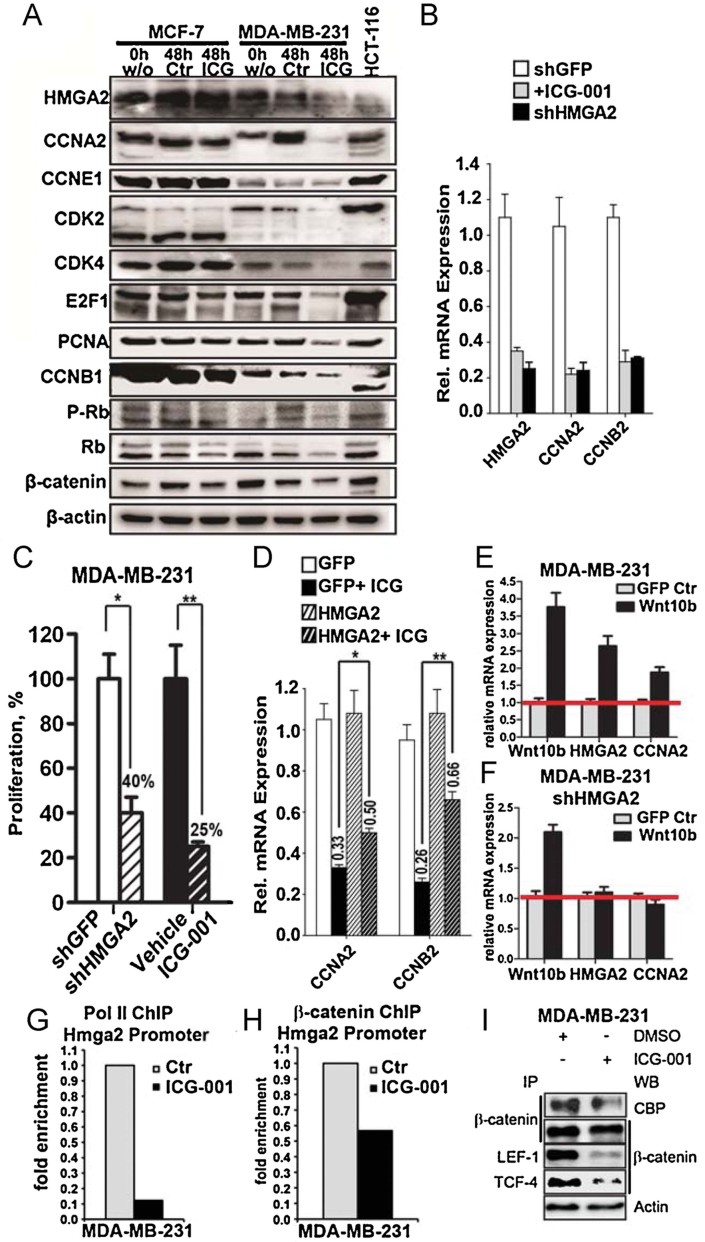
Inhibition of Wnt/β-catenin signalling in human TNBC leads to decreased expression of HMGA2 and cell cycle regulators by blocking CBP/LEF/TCF-dependent transcriptional activity **A.** Effect of ICG-001 (10 µM)-induced inhibition of Wnt/β-catenin signalling on the expression of cell cycle regulators and proliferation markers in synchronized (0 h) and released (48 h) human MCF7 (non-triple-negative) and MDA-MB-231 (triple-negative) cells, as detected by Western blot analysis. One percent DMSO was used as control treatment. β-actin served as a loading control. HCT-116 colorectal carcinoma cells served as control cells.**B.** Reduced expression of *CCNA2* and *CCNB2* in MDA-MB 231 cells after *HMGA2* knockdown or ICG-001 treatment, as determined by qPCR. Error bars represent the means and the standard deviations from three independent experiments.**C.** Quantification of proliferation of MDA-MB-231 cells after shRNA-mediated knockdown of *HMGA2* or ICG-001 treatment (10 µM). Error bars represent the means and the standard deviations from four independent experiments; *p values = 0.02 and **p = 0.008 *versus* control treatments (Student's *t*-test).**D.** qt-PCR expression analysis for *CCNA2* and *CCNB2* in control (pcDNA3) and *HMGA2*-overexpressing (pcDNA3-HMGA2) MDA-MB-231 cells treated with vehicle control or ICG-001. Error bars represent the means and the standard deviations from five independent experiments; *p values = 0.01 and **p = 0.004 *versus HMGA2*-overexpressing cells (Student's *t*-test).**E, F.** WNT10B-induced upregulation of *CCNA2* depends on *HMGA2*, as determined in an epistasis experiment and analysed by qPCR. Error bars represent the means and the standard deviations from three independent experiments.**G, H.** qt-PCR analysis of ChIP experiments with MDA-MB-231 cells after release from growth arrest and ICG-001 treatment (48 h) using RNA Pol II and β-catenin antibodies. Values were normalized to both the input template and a nonregulated locus (*hHBB*).**I.** Immunoprecipitation and Western blot analysis illustrating a CBP/LEF/TCF-dependent inhibition of β-catenin signalling by ICG-001 in human TNBC MDA-MB-231 cells. Actin served as a loading control. *p* Values of <0.05 were considered to be statistically significant (C, D).

We next revisited our previously defined ‘intrinsic proliferation signature’ from our *Wnt10b*-driven tumours (Supporting Information Fig S3A and B) and hypothesized that a translational TNBC model should preserve this signature in human TNBC cells. Moreover, these proliferation genes might be the target(s) for ICG-001-mediated growth arrest. To test this, we synchronized both MCF7 and MDA-MB-231 cells at G_1_-phase by contact inhibition (for 2–3 days) and then harvested and released the cells for 16 h [S-phase; (Miranda-Carboni et al, 2008)]. We treated the released cells with ICG-001 for an additional 48 h. Whole cell extracts were analysed by immunoblotting for HMGA2 and proliferation markers in the presence or absence of ICG-001 (Fig 5A). HMGA2, Cyclin A2, CDK2, CDK4, E2F1 and PCNA protein expression were down-regulated in MDA-MB-231 cells treated with ICG-001. These results are consistent with a cell cycle arrest phenotype. In contrast, MCF7 cells had no significant expression changes of these proliferation markers. Cyclin E1 and B1 were unchanged in both cell lines. HCT-116 colon cancer cells have constitutively active *Wnt/β-catenin* signalling but do not express as high levels of HMGA2 as in TNBC cell lines (Fig 5A).

We next wanted to determine if cell cycle arrest protein activity is restored upon ICG-001 treatment and hypothesized that RB would be hypo-phosphorylated due to the loss of CDK2 and 4. To test this we probed the same protein extracts with both a phospho-specific antibody (pRB {Ser807/811}) that can measure Cyclin D1/CDK4 and/or Cyclin A2, E/CDK2 activity and an antibody detecting total RB (Fig 5A). In MDA-MB-231 cells, 48 h after release, RB is hyper-phosphorylated (pRB) and in the presence of ICG-001 this hyper-phosphorylation is lost, consistent with the loss of both CDK4 and CDK2 enzymatic activity.

To ensure that the observed results were due to the loss of transcriptional activation by β-catenin and not mediated by protein turnover we concurrently conducted qt-PCR on MDA-MB-231 cells for *HMGA2*, proliferation markers and *BIRC5* [Survivin, a well-known target of ICG-001 in colon cancer (Emami et al, 2004; Supporting Information Fig S5A)]. We further validated the effects of ICG-001 on various subtypes of TNBC cells lines and purchased commercially available cell lines from ATCC to ensure authenticity. ICG-001 treatment down regulated HMGA2 and two of its downstream targets (CCNA2 and CCNB2) and BIRC5 a known direct target of ICG-001 in a subset of MDA-MB derived cell lines (Supporting Information Fig S5B). *HMGA2* mRNA level is down-regulated in the presence of ICG-001, suggesting that it is directly regulated by β-catenin/CBP interactions. We also silenced β-catenin with siRNA (80% reduction) and verified the loss of *HMGA2* (30% reduction), which was accompanied by reduced *WNT10B* expression (80% reduction) amongst other targets (Supporting Information Fig S5C). Corroborating our hypothesis, ICG-001 treatment of BTL10 cells also led to down-regulated mRNA levels of several *Cyclins*, *HMGA2*, and *BIRC5* (Supporting Information Fig S5D). Taken together, these results argue that the loss of protein expression of our proliferation markers is due to the loss of transcriptional activity mediated by β-catenin signalling and not protein turnover.

To determine if HMGA2 is necessary and sufficient for proliferation of MDA-MB-231 cells we conducted genetic loss-of-function and gain-of-function assays. To this end we genetically silenced *HMGA2* expression utilizing the human *Lentiviral-shHMGA2* and *shGFP* system, as previously mentioned. There is a 60% decrease in proliferation in the *shHMGA2* cells (*p* < 0.05), which is very similar to the growth inhibition in the parental cells when treated with ICG-001 (75% reduction, *p* = 0.01; Fig 5C). Next, we exposed *shGFP* control cells to ICG-001 treatment for 48 h and compared it to *shHMGA2* untreated cells by analysing the mRNA expression levels of *HMGA2*, *CCNA2* and *CCNB2* using qt-PCR (Fig 5B). We observed a >70% downregulation for all the genes tested in the *shHMGA2* clones, similar to the ICG-001-induced reduction in *shGFP* control cells.

To rescue the inhibition phenotype we transfected MDA-MB-231 cells with *pcDNA3-GFP* and *pcDNA3-HMGA2* vectors for 24 h and subsequently treated with ICG-001 for an additional 24 h. The cells were harvested and qt-PCR was conducted for *CCNA2* and *CCNB2* (Fig 5D). HMGA2 overexpression was able to moderately rescue both *CCNA2* (>30% *p* < 0.05) and *CCNB2* (60% *p* = 0.01) in the presence of ICG-001. The above results would suggest that HMGA2 is essential and necessary for cellular proliferation of MDA-MB-231 cells that depend on β-catenin signalling.

To test our model for the order of action of genes in a regulatory hierarchy that governs the *Wnt10b/β-catenin* signalling pathway, we designed an epistatic functional assay for *WNT10B*. We transfected MDA-MB-231 cells with *pcDNA3-GFP* and *pcDNA3-WNT10B* vectors, cells were harvested and qt-PCR was conducted for *WNT10B*, *HMGA2* and *CCNA2* (Fig 5E). Concurrently, we repeated the previously mentioned transfection with our MDA-MB-231-*shHMGA2* silenced verified cell lines (Fig 5F). WNT10B induced the expression of *HMGA2* and its known downstream target *CCNA2* in control cells. In contrast, cell lines silenced for *HMGA2* can still express *WNT10B* but the expression of *HMGA2* and its downstream target *CCNA2* is lost. The above experiment supports the epistatic activity of WNT10B on both *HMGA2* and *CCNA2* gene expression in triple-negative MDA-MB-231 cells.

We searched the literature and found that the mechanism for the disruption of β-catenin and CBP protein–protein interaction, in the presence of *Wnt* inhibitor ICG-001, had mostly been characterized in colon cancer cells (Emami et al, 2004). We therefore wanted to verify that similar biochemical interactions were also relevant for HMGA2 regulation in MDA-MB-231 cells. To this end we utilized our previously mentioned *in silico* analysis to identify potential TCF/LEF1-binding sites that could represent Wnt response elements (WRE) within the human *HMGA2* loci (http://genome.ucsc.edu/: for the genomic upstream sequences and http://www.gene-regulation.com/cgi-bin/pub/programs/patch/bin/patch.cgi: PATCH analysis for transcription binding sites). We conducted ChIP analysis by examining *HMGA2* promoter occupancy by RNA Polymerase II (RNA Pol II) and β-CATENIN in MDA-MB-231 cells exposed for 48 h to ICG-001 (10 µM; Fig 5G and H). RNA Pol II occupancy was disrupted by 10-fold and loss of β-CATENIN occupancy on the *HMGA2* promoter was two-fold. These results support that the direct regulation of HMGA2 by β-catenin signalling is conserved from mouse (Fig. 3) to humans.

To gain further insight into the mechanism of action mediated by ICG-001 treatment in MDA-MB-231 cells, we conducted a set of IP experiments to test for the known transcriptional machinery of the *Wnt* signalling pathway (Fig 5I). We first immunoprecipitated β-CATENIN and immunoblotted for CBP to show that the biochemical protein–protein interactions are diminished in the presence of ICG-001. The above experiment was then repeated and immunoblotted for β-CATENIN and, as expected, no loss of overall protein levels for β-CATENIN in the presence of ICG-001 could be detected. We next conducted IP's for both LEF-1 and TCF-4 proteins individually and immunoblotted for the presence of β-CATENIN protein levels. We observed that in both cases β-CATENIN protein–protein interactions with either LEF-1 or TCF-4 were dramatically decreased after ICG-001 treatment.

Taken together, these data suggest that the ICG-001-induced loss of cell cycle proliferation is mediated in part by decreasing expression of the ‘intrinsic proliferation signature’. Mechanistically, cell cycle arrest is restored in the presence of ICG-001 by disruption of β-CATENIN protein–protein interaction with CBP, LEF-1 and TCF-4. More importantly, HMGA2 is both necessary and sufficient to maintain proliferation in TNBC cell lines and that WNT10B has an epistatic effect on *HMGA2* expression and its subsequent downstream target *CCNA2.*

### HMGA2 predicts survival outcome in TNBC

**Figure 6:**
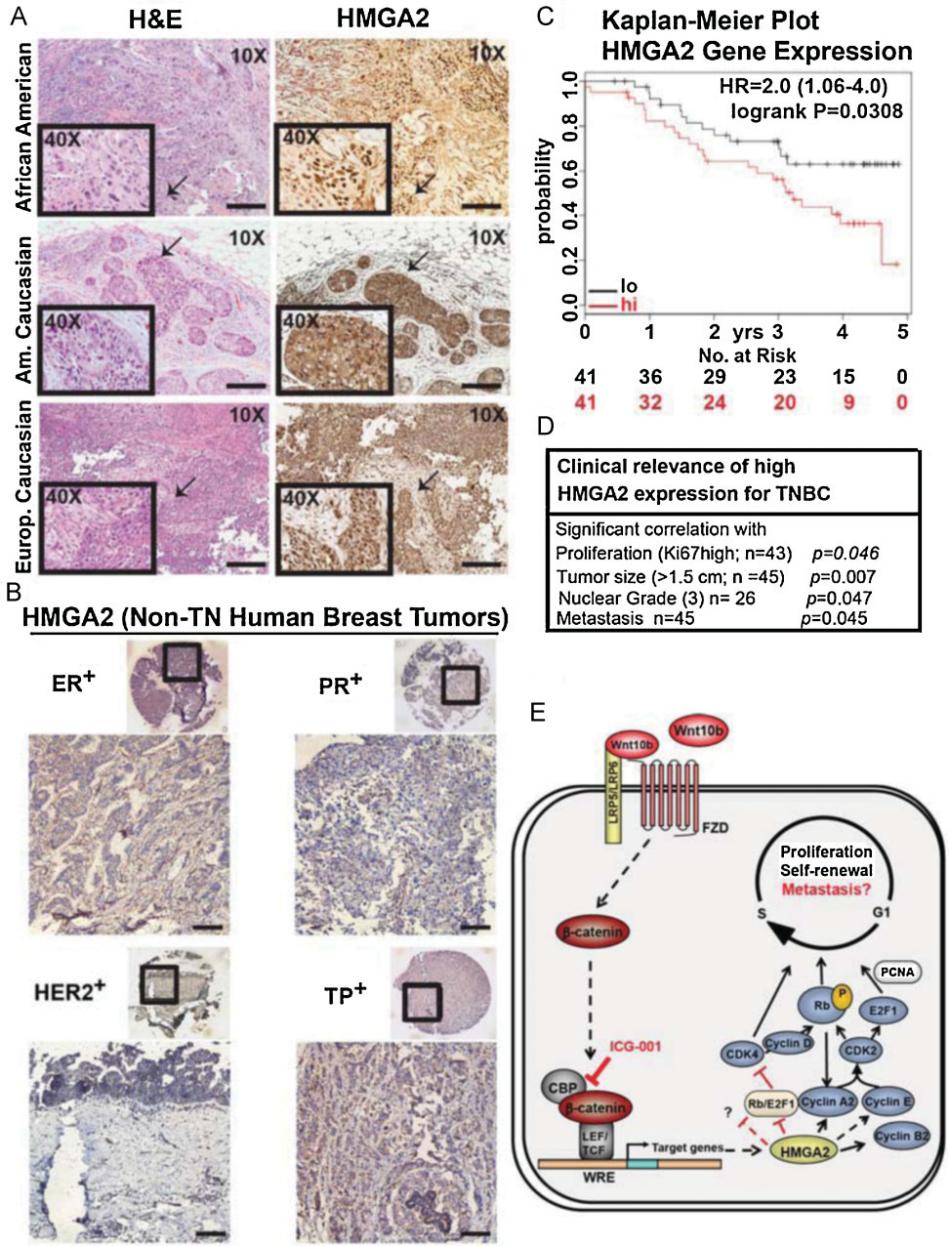
Triple-negative human breast cancers specifically express high levels of nuclear HMGA2, whose expression has clinical relevance and predicts recurrence-free survival of TNBC patients Detection of nuclear HMGA2 in human TNBC from different patient groups, as shown by IHC of tissue microarrays. hematoxylin–eosin (H&E) staining from an adjacent section is shown on the left. Arrows indicate enlarged areas shown in insets (40×). Bar, 200 µm.Absence of nuclear HMGA2 expression in other subtypes of human breast cancer (ER^+^, PR^+^, HER^+^, and triple-positive), as analysed by IHC. Bar, 200 µm.Kaplan–Meier survival analysis showing significantly improved recurrence-free survival of Basal-like breast cancer patients with tumours that express lower levels of HMGA2. Patients with higher (red) and lower expression (black) are indicated as well as numbers of patients at risk at specific time points (below each diagram). Hazard ratios (HR) and *p* values (log rank *p*) are depicted for each survival analysis. Kaplan–Meier survival data were generated using the publicly accessible online tool KM-plotter.High HMGA2 expression in TNBC is significantly correlated with large tumour size, high proliferation, high nuclear grade and metastasis (HMGA2 expression was determined by IHC in TNBC patient samples and results were correlated with patient data). Associations between markers and clinical parameters that were measured on a continuous or ordinal scale were evaluated using Kendall's Tau coefficient. Associations between markers and binary clinical parameters were tested using an exact trend test, associations with nominal parameters were tested using Fisher's exact test. *p* Values and patient numbers (*n*) are indicated in the table. Additional information can be found in Supporting Information Fig S7 and the Materials and Methods Section. *p* Values of <0.05 were considered to be statistically significant (C, D).Proposed model for WNT10B/β-catenin signalling inducing HMGA2 expression. WNT10B activates canonical Wnt/β-catenin signalling leading to up-regulation of HMGA2 and self-renewal by induction of cell cycle proliferation. ICG-001 disrupts CBP/β-catenin interaction leading to the loss of HMGA2. Black dash arrows and red dash blocking bars represent a possible mechanism of HMGA2 action and/or loss of β-catenin transcriptional activity.

Based on the ability of WNT10B to have clinical significance in TNBC, we hypothesize if HMGA2 would be able to have the same outcome. To this end we conducted IHC analysis for HMGA2 in 14 samples from African-Americans (AA; from The University of Chicago), 31 Caucasian-American samples (from Cedars-Sinai Medical Center) and 14 German samples. There is strong expression of nuclear HMGA2 protein in the TNBC samples (Fig 6A and Supporting Information Fig S6A–C). Furthermore, nuclear HMGA2 expression is restricted to TNBC as it was not detectable in ERα^+^, PR^+^, HER2^+^ and triple positive (TP) breast cancer samples (Fig 6B). More interestingly, HMGA2 expression was not detected at all or at very low levels in four adjacent ‘normal’ breast tissue biopsies, which are from the AA TNBC samples (Supporting Information Fig S6D). We determined the frequency of nuclear HMGA2 expression to be >80% in our TNBC sample set (Supporting Information Fig S6E).

We next determined if, like *WNT10B*, *HMGA2* could predict survival outcome in the previously mentioned KM-plotter basal-like data set (Fig 1E and F; Gyorffy et al, 2010). Analysed as previously described, Kaplan–Meier survival probabilities with a medium cutoff for *HMGA2* were generated (Fig 6C). Remarkably, basal-like patients with high *HMGA2* mRNA levels (80% of the patients) had a significant lower probability of survival than those with low *HMGA2* levels [log-rank test *p* = 0.0308, hazard ratio (HR) = 2.0], albeit fewer patients passed the criteria than in the WNT10B analysis (Fig 1E).

Next we illustrate that in our cohort of TNBC samples HMGA2, like WNT10B, has predictive value for tumour size >1.5 cm (*n* = 45, *τ* = 0.33, *p* = 0.007) and high nuclear grade status 3 (*n* = 26, *τ* = 0.37, *p* = 0.047; Fig 6D and Supporting Information Fig S7A–D). Remarkably, and unlike WNT10B, HMGA2 was significantly associated with Ki67 status (*n* = 43, *τ* = 0.24, *p* = 0.046) and metastasis (*n* = 45, *p* = 0.045).

In summary, we have presented evidence supporting that HMGA2 expression is a clinical marker that has the capacity to predict survival outcome in TNBC and basal-like breast cancer patients. We validated the above by showing the restricted expression of HMGA2 only in primary TNBC samples and not in ‘normal’ breast tissue or in other breast cancer subtypes.

## DISCUSSION

Breast cancer heterogeneity is vast even within the five subtypes identified originally from microarray analysis (Sorlie et al, 2003) or within the 10 subtypes identified from whole-exome sequences of DNA (Banerji et al, 2012). That provides major challenges such as identification of the underlying molecular mechanisms and development of specific therapeutics for different subtypes of breast cancer. Triple negative breast cancer has been shown to be a very diverse group of cancers with at least six classifications (Lehmann et al, 2011; Marotta et al, 2011; Perou, 2010). Furthermore, important questions remain: why does TNBC become resistant to chemotherapy? Why do malignant TNBC develop? Therefore, the generation of *in vivo* models that highly resemble a given subtype of breast cancer is of enormous importance.

In this study, we determined if the global gene expression pattern of *Wnt10b*-driven mouse mammary tumours carries an ‘intrinsic gene signature’, which can be translated to the known phenotypes associated with human TNBC. We provide evidence that *Wnt10b*-induced mammary tumours are devoid of ERα, PR or HER2, and thus can be classified as TN. Furthermore, the consistent expression of other markers such as nuclear β-catenin, *Axin2*, CK5 and CK6, and a specific subset of cell cycle regulators demonstrate the high similarity of the mouse and human TN mammary tumours.

We observed β-catenin over-expression in >80% of our human TN breast cancer samples. Although all cells may not have apparent nuclear localization by IHC it is known that cytoplasmic accumulation of β-catenin is also an indicator for activated Wnt/β-catenin signalling and can predict survival outcome (Geyer et al, 2011; Khramtsov et al, 2010; Lugli et al, 2007; Martensson et al, 2007; Wong et al, 2003; Supporting Information Fig S8). Moreover, active gene transcription of a known direct target of canonical *Wnt*-signalling, AXIN2, by both ISH and IHC demonstrates transcriptional activation by β-catenin. Additionally, we correlate the expression of the WNT10B-ligand in similar regions but we cannot at this time exclude that other WNT-ligands mediate canonical Wnt-signalling (*i.e.* WNT1 or WNT3A amongst others) that may be responsible for the above observation. We hypothesized that in breast cancer, β-catenin over-expression during tumorigenesis is driven by components of Wnt family members, such as WNT10B or another Wnt-ligand (Klemm et al, 2011) rather than by mutations of members of the Wnt/β-catenin pathway as is the case for sporadic colon cancer (Klaus & Birchmeier, 2008). To this end we illustrate that WNT10B has a predictive survival outcome in both TNBC and basal-like human breast cancer. In contrast, WNT1 ligand did not have a predictive survival outcome.

It is well established that signalling components that function transiently during embryonic development might become an oncogene by constitutively re-activating embryonic signalling programs in adult tissue(s) (Polyak & Weinberg, 2009). To this end we provide evidence that HMGA2 is specifically expressed in the mammary anlagen during embryogenesis (E14.5) at the same period as WNT10B expression is high and, furthermore, HMGA2 is lost in the absence of WNT10B. Using genetic gain- and loss-of-function assays we show that HMGA2 is both necessary and sufficient for proliferation of TNBC cells. More importantly, we provide evidence that WNT10B has epistatic function on HMGA2 and its downstream target CNNA2. This interpretation further supports that during embryogenesis and tumorigenesis both WNT10B and HMGA2 have an order of action that in a regulatory hierarchy governs the *Wnt*-signalling pathway. We have linked HMGA2 regulation by *Wnt10b/β-catenin* signalling from the embryonic mammary gland anlagen to triple-negative breast cancer.

We identified HMGA2 to be highly expressed in *Wnt10b*^*LacZ*^ TN tumours and in both human TNBC cell lines and primary TNBC tumour samples. HMGA2 has predictive value in both TNBC and basal-like patients. More importantly, high HMGA2 expression is significantly correlated with TNBC metastasis (*p* = 0.045). Thus, HMGA2 is an excellent candidate as the readout for patients who may undergo clinical-trails with ICG-001 or *Porcupine* inhibitors, that is, to measure their progression if responding to treatment. Our model provides evidence for both secretion of *Wnt*-ligand and downstream activation of β-catenin to modulate HMGA2 expression and proliferation.

Mechanistically, our data now provide evidence that WNT10B activates canonical β-catenin signalling leading to proliferation and up-regulation of HMGA2 in TNBC directly by components of the Wnt canonical signalling pathway. Furthermore, the Wnt/β-catenin inhibitor ICG-001 can significantly suppress mRNA and protein levels of HMGA2 and block proliferation in TNBC cells (model in Fig 6E). Our data also illustrate that HMGA2 expression is significantly correlated with the capacity to predict metastasis in our relatively small TNBC cohort samples (*i.e.* 12/45; Fig 6D and Supporting Information Fig S7D). Accordingly, we observed that *Wnt10b*^*LacZ*^ transgenic mice give rise to rare spontaneous lung metastasis expressing high levels of *Hmga2* and HMGA2 in FACS-sorted Lin^−^LacZ^+^ lung metastasis cells (Wend et al, 2012). The above results support the rationale that the *Wnt10b/β-catenin/Hmga2* axis needs to be further tested for their functional role in TNBC and related lung-metastasis in a preclinical experimental model. In conclusion, the important clinical relevance of our results and the discovered mechanism is proven by the negative survival outcome of TN breast cancer patients over-expressing *HMGA2* as well as *WNT10B*. Thus, inhibition of the *Wnt10b/β-catenin/HMGA2* signalling axis in TNBC patients could open new opportunities for future therapies.

## MATERIALS AND METHODS

### Human breast cancer tissues

Studies based on formalin-fixed paraffin-embedded samples and details can be found in the Supporting Information and Supporting Information Table S1. Informed consent was obtained from all subjects and experiments conformed to the principles set out in the WMA Declaration of Helsinki and the NIH Belmont Report. Studies were approved by the Institutional Review Boards of the collaborating pathologists and the University of California at Los Angeles.

### Mice

*MMTV-Wnt10b*^*TG*^ mice were described (Miranda-Carboni et al, 2008) *MMTV-Wnt10b-IRES-LacZ* mice will be described elsewhere (Miranda-Carboni et al. Generation of a *MMTV-Wnt10b-IRES-LacZ* transgenic mouse model. Manuscript in preparation). Wnt10b^*−/−*^ mice were described (Miranda-Carboni et al, 2008) and have been de-derived using the same strategy as previously described (Stevens et al, 2010) with an identical phenotype as previously published. The study was approved by the Office of Animal Research Oversight of the University of California at Los Angeles.

### Cell isolation and FACS analysis

Cell isolation, Lineage (Lin-)-depletion, cell labelling and FACS analysis were carried out as previously described (Stingl et al, 2006). EasySep™ immunomagnetic cell separation was utilized according to the manufacturer's protocol (Stemcell Technologies, Cat.# 19757). Details can be found in Supporting Information Materials and Methods Section.

### Cell culture, cell synchronization and proliferation assay

All cells were maintained in a humidified atmosphere with 5% CO_2_ in DMEM plus 1% Pen/Strep and 10% FCS. Mammary cell medium contained 1 µg/ml insulin. Synchronization of cell lines at G1, for cell cycle progression analysis and MCF7-Wnt10b cells were described elsewhere (Miranda-Carboni et al, 2008) ICG-001 (10 µM in 1% DMSO) was used in experiments with synchronized cell as follows: treatment began 16 h post-release (*i.e.* S-phase) for an additional 48 h. Cell proliferation was measured using WST-1 cell proliferation assay (Roche), according to the manufacturer's protocol.

### Knockdown experiments

All siRNA oligonucleotides were purchased from Thermo Scientific (Dharmacon) as specific oligo pools (SMARTpool). Oligonucleotide sequences for transient RNAi can be found in Supporting Information Table S2. Thirty picomole of β-catenin (CTNNB1) or Luciferase siRNA were transfected by electroporation with Amaxa Nucleofector (Lonza) according to the manufacture's protocol. Cells were used for experiments 48 h after transfection. MDA-MB 231 and *WZA*^*LAcZ*^ cells were transduced with either human or mouse for four target-sets of pLKO.1-ShHMGA2 (Thermo-Open Biosystems) and with pLKO.1-ShGFP (Addgene) using standard protocols. Clones were selected by puromycin.

### Transient transfections

MDA-MB231 cells were transfected using electroporation with an Amaxa Nucleofector (Lonza: pcDNA-GFP) following the manufacturer's protocol. Plasmids are identified in the text (pcDNA3.1, pBA-WNT10B (from Addgene, and subsequently cloned into pcDNA3.1) and pcDNA3.1HMGA2 (from Addgene).

### Mammosphere formation assay (MSA)

MSA cultures were carried according to manufacturer's protocol (Stemcell Technologies) using ultra-low adherence dishes (Nalge Nunc) and complete Mammocult culture medium (Stem Cell, Inc.) containing ICG-001 (10 µM) or DMSO (1:1000) as control (each *n* = 3). Details can be found in the Supporting Information Materials and Methods Section.

### Immunohistochemistry (IHC) and *in situ* hybridization (ISH)

Formalin-fixed paraffin-embedded samples were stained as described (Miranda-Carboni et al, 2008) and counterstained with hematoxylin QS (Vector Labs). ISH was performed as described (Huelsken et al, 2000) and sections were counterstained using nuclear fast red (Vector Labs). Details can be found in the Supporting Information Materials and Methods Section.

### Immunohistochemistry evaluation

Three observers (P.W., M.Y., M.J.) performed quantitative analysis of the tissue specimen without knowledge of specimen identification. Scoring was based on intensity and percentage of positively stained cells for WNT10B and HMGA2 by immunohistochemistry as follows: low; 1–30%, medium; 30–70%, high; ≥70% (Supporting Information Table S1). All discrepancies were resolved by a second examination using a multihead microscope.

### Cell extraction and Western blotting

Cells were lysed as previously described (Miranda-Carboni et al, 2008). Fifty to 250 µg of protein were loaded per lane and separated by SDS–PAGE 10% gels. After transfer, Immobilon-P (Millipore) was immunoblotted using primary antibodies (for antibody details see Supporting Information Materials and Methods Section). ImmunoPure-peroxidase conjugated secondary antibodies (Thermo Scientific) were used according to manufacturer's protocols.

### Immunoprecipitation

Immunoprecipitation reactions were conducted on whole-cell lysates from *WZA*^*LacZ*^ and MDA-MB-231 cells that were treated with vehicle control (DMSO) or ICG-001 (10 µM, 48 h). Immunoprecipitation reactions were conducted with β-catenin (sc-7199 H-102, Santa Cruz), LEF-1 (sc-8591 N-17, Santa Cruz) and TCF-4 (#2206, Cell Signaling) antibodies on 200–1500 µg of total protein and blotted with previously described antibodies.

### Chromatin immunoprecipitation (ChIP)

Cells were grown to confluency for 2–3 days and harvested at 0 and 8 h post-release. ChIP was performed as described previously (Krum et al, 2008a, b) Antibodies used included RNA pol II (#17-672, Millipore) and β-catenin (sc-7199 H-102, Santa Cruz). Each experiment was repeated at least three times. Primer pairs for each gene are provided (Supporting Information Tables S3 and S4). Additional details, materials and methods are available in the Supporting Information Material.

### Statistical methods

Due to the large number of comparisons of patient samples the analysis is considered exploratory. Associations between markers and clinical parameters that were measured on a continuous or ordinal scale were evaluated using Kendall's Tau coefficient (*τ*). Associations between markers and binary clinical parameters were tested using an exact trend test, associations with nominal parameters were tested using Fisher's exact test. Calculations were performed using the SAS system, version 9.1.3. A *p*-value of <0.05 was considered to be statistically significant. The publically available database KM-plotter was used to assess 22,277 genes in over 3000 breast cancer samples [http://kmplot.com/breast/; (Gyorffy et al, 2010)]. The KM-plotter software calculated the hazard ratio using Cox's proportional hazard model, and the score test of the proportional hazard model is equivalent to the log-rank test. For all other experiments statistical analysis was performed using Prism 5 software including Student's *t*-test and one-way ANOVA, and all graphs were generated using Microsoft Excel and Prism 5 software. The error bars were calculated and represented in terms of mean ± SD.

For more detailed materials and methods including bioinformatics, qt-PCR, primers, reporter gene assay and patient clinical information see the Supporting Information.

## For more information

Wnt homepage: http://www.stanford.edu/group/nusselab/cgi-bin/wnt/

KM-Plotter Breast and Ovarian dataset: http://kmplot.com/analysis/

Breast Cancer Homepage of the National Cancer Institute: http://www.cancer.gov/cancertopics/types/breast

Miranda-Carboni Lab Homepage: http://www.cancer.ucla.edu/index.aspx?page=645&recordid=547

## Supplementary material

As a service to our authors and readers, this journal provides supporting information supplied by the authors. Such materials are peer reviewed and may be re-organized for online delivery, but are not copy-edited or typeset. Technical support issues arising from supporting information (other than missing files) should be addressed to the authors.
