# Supplementary material for: WNT10B/β-catenin signalling induces HMGA2 and proliferation in metastatic triple-negative breast cancer
Source: EMBO Mol Med. 2013 Jan 11;5(2):264–79. doi: 10.1002/emmm.201201320 (PMC3569642; doi:10.1002/emmm.201201320)
Supplement: Supplementary file 1 [file emmm0005-0264-SD1.pdf]

EMBO Molecular Medicine

WNT10B/β-catenin Signaling Induces HMGA2 and Proliferation in Metastatic Triple-Negative Breast Cancer

Peter Wend, Stephanie Runke, Korinna Wend, Brenda Anchondo, Maria Yesayan, Meghan Jardon, Natalie Hardie, Christoph Loddenkemper, Ilya Ulasov, Maciej S. Lesniak, Rebecca Wolsky, Laurent A. Bentolila, Stephen G. Grant, David Elashoff, Stephan Lehr, Jean J. Latimer, Shikha Bose, Husain Sattar, Susan A. Krum and Gustavo A. Miranda-Carboni

Corresponding author: Gustavo Miranda-Carboni, David Geffen School of Medicine at UCLA

Review timeline:

Submission date:	24 February 2012
Editorial Decision:	24 March 2012
Revision received:	07 August 2012
Editorial Decision:	15 September 2012
Revision received:	26 October 2012
Editorial Decision:	07 November 2012
Accepted:	14 November 2012

Transaction Report:

(Note: With the exception of the correction of typographical or spelling errors that could be a source of ambiguity, letters and reports are not edited. The original formatting of letters and referee reports may not be reflected in this compilation.)

1st Editorial Decision 24 March 2012

Thank you for the submission of your manuscript to EMBO Molecular Medicine. We have now heard back from the three referees whom we asked to evaluate your manuscript.

Although the referees find the topic of the study important, they also raise a number of substantial concerns about the conclusiveness of the results and technical issues, which should be convincingly addressed in a major revision of the current mnanuscript.

As you will see from the enclosed reports, the article should be streamlined to clarify the message as suggested by both referees 1 and 3. All three reviewers are concerned with lack of controls, lack of or inappropriate statistical analysis, and unconvincing experiments. They do suggest, however, ways of improving the manuscript that would involve a lot of additional (experimental) work. I am uncertain whether you will be able (or willing) to return a revised manuscript within the 3 months deadline and I would understand your decision if you choose to rather seek rapid publication elsewhere.

Nonetheless, while it is clear that publication of the paper cannot be considered at this stage, I would be open to the submission of a revised manuscript providing that the referees' concerns must be fully addressed and that acceptance of the manuscript would entail a second round of review. I would add that it is particularly important that all of their suggestions are taken on board as we cannot consider its publication otherwise.

I should remind you that it is EMBO Molecular Medicine policy to allow a single round of revision only and that, therefore, acceptance or rejection of the manuscript will depend on the completeness

of your responses included in the next, final version of the manuscript.

I look forward to seeing a revised form of your manuscript as soon as possible.

Should you find that the requested revisions are not feasible within the constraints outlined here and choose, therefore, to submit your paper elsewhere, we would welcome a message to this effect.

Yours sincerely,

Editor
EMBO Molecular Medicine

***** Reviewer's comments *****

Referee #1 (Other Remarks):

In this manuscript, Wend et al provide evidence that transgenic murine WNT10B-driven tumors resemble human triple negative breast cancers. The authors suggest that WNT10B signaling activates beta-catenin leading to HMGA2 upregulation and proliferation. They go on to suggest that this observation applies to CD44+ cells, which is one of the known self-renewal markers in mammary epithelial cells. Finally, the authors use a chemical inhibitor of Wnt/B-catenin pathway to validate some of their findings. Identifying mouse models that mimic triple negative breast cancer is of interest given its current absence. The mechanisms that contribute to the triple negative breast cancer phenotype have been the center of much attention since this could be pivotal to tackle this aggressive disease.

This manuscript identifies an interesting model that could serve the study of triple negative breast cancer in the mouse and the description is largely convincing. However, this represents and incremental but not a substantial advance since the mechanistic analysis and the phenotypic explanations are rather confusing, not hypothesis driven and lack of important controls and gaps.

Major Points:

1. The manuscript is a collection of evidences, with important gaps that need to be filled. First, the authors show that when ER negative (not triple negative) breast cancer primary tumors are separate according to WNT10A mRNA levels but not WNT1 these two groups behave differently in terms of relapse (log rank test). It is not clear whether ERnegative patients that express WNT10b are TNBC or not. In turn, the authors, based on a small series of IHC analysis, suggest that protein levels exert this function and, in turn, this is specific of triple negative breast cancer. Unfortunately, no statistics are draw. Then the authors describe a transgenic mouse model that express WNT10b. These mice develop mammary tumors that resemble TNBC. This is an interesting observation, however how does WNT10b generate this phenotype is not clear at all. The authors suggest is via HMGA2, but do not show how. Moreover, they claim that HMGA2 triggers a self-renewal phenotype and more proliferation but they do not show what is the relationship with the TNBC. Overall, the message is not clear, the reader gets confused and no mechanistic link between WNT10b, signaling, expression of HMG2A and contribution of HMG2A to TNBC phenotype is observed. The authors need to make an important effort to streamline these points, connect them and provide data that fills the gaps.

2. The authors' state (page 4, third paragraph) based on the literature that 50% of breast cancers have nuclear b-catenin. However, when they analyze their patient sample series, they restrict WNT10b to a small subset of TNBC samples. They go on and attribute to tumors that express WNT10b TNBC properties. However, if 50% of breast cancer tumors have nuclear b-catenin in the nucleus (meaning Wnt signaling is ongoing) and >60% of breast cancer patients are ER+, it is unlikely that WNT10b through Wnt signaling leads to TNBC in a representative cohort of breast cancer patients. Indeed, this suggests that Wnt signaling (nuclear b-catenin) is not responsible for the TNBC phenotype and that other things, possibly downstream of WNT10b, are more relevant to establish such phenotype. The key point is what is responsible for TNBC independently of Wnt-signaling. A question that the authors do not address. Moreover, this point questions their transgenic mice model. Thus, prompting for the request to induce MMTV-WNT10b driven expression in a different genetic background to

confirm their observation.

3. As highlighted before, the authors should include data referring to the additional 24 TNBC samples from the cohorts from LA and Chicago. Moreover, statistics depicting the correlation between nuclear b-catenin and Wnt10b should be draw. Finally, a comparison with other breast cancer tumors subtypes is also necessary. It must be shown whether this correlation is unique of TNBC or it also occurs in other breast cancer subtypes (plus statistics).

4. Again, association between CD44 and TNBC markers (including nuclear b-catenin) lacks statistics. Significance is missing and without it no claims can be made. Similarly, this applies for WNT10b and AXIN2.

5. Page 8, second paragraph. The authors state that WNT10b expression was validated to be high in TNBC. The authors should stick with the data. The data depicted indicates that WNT10b is relevant in ER negative breast cancer but not in TNBC (the latter is included in the earlier). Finally, since the authors claim prediction of risk, COX proportional hazard analysis instead of Log Rank test should be performed.

6. The authors identify WNT10b as a potential marker of TNBC. How is it accumulated, expressed of induced in TNBC tumors?

7. The authors claim that MMTV-WNT10b mice BC tumors are enriched for cells with high self-renewal potential. Surprisingly, they define tumor-initiating cells (page 9, 2nd paragraph) as CD44high, CD24low. However, through their manuscript they only focus on CD44high, CD24 high. How come? A very convincing explanation must be provided. Clearly, their statement in page 22 and 23 of the discussion does not help, 2 out of the 3 arguments provided highlight the importance of CD24low for aggressiveness and stemnes.

8. The authors claim that the self-renewal marker HMGA2 is expressed and provides self-renewal functions. However, in figure 3b it can be observed how unsorted cells expressed higher levels of HMGA2 than tumor initiating cells. Thus, the stem cell population does not experience the higher induction of this gene. These results suggest that this protein might be necessary but not sufficient to explain this WNT10b driven phenotype. On top of that, how does WNT10b regulate HMGA2?

9. Inhibitors are very dangerous toys. They are accompanied with uncontrolled side effects. Thus, the authors must use and independent compound that targets the same principle to ensure robustness. Alternative, they could use a b-catenin shorthairpin construct to validate some of their key experiments.

10. Page 16, first paragraph. In figure 5 the authors use ICG-001 to block b-catenin signaling and test downregulation of HMGA2, CyclinA2 etc... The question is: what does happen in MCF710b cells? This is of particular interest given in these cells all nuclear b-catenin signaling is, in principle, generated by WNT10b.

11. The authors used the MDA-MB-231 cells to test whether ICG-100 cause an induction of INK4 locus CDK inhibitors. These results are puzzling, particularly because there are several reports in the literature claiming that these breast cancer cells are INK4 locus deleted (i.e Bisonga et al Cancer Genetics and Cytogenetics 125 (2001) 131-138 and others). This locus includes p16, p19 and p15. Thus it is very strange that the authors do not observe activation of p16 and p19 but observe activation of p15. Given the literature reports, the authors must verify that their MDA-MB231 cells have an intact INK4 locus (including INK4b).

12. To determine if HMGA2 is necessary and sufficient for proliferation of MDA-231 cells downstream of WNT10b, the authors should test if WNT10b exogenous expression effect is completely abolished by shHMGA2.

13. Given the same sample set was used to evaluate the differences in disease-free survival between groups that express low and high WNT10b and HMG2A, a correlation between the expression of both genes and its significance should be draw as this is pivotal to the authors claims.

Minor

1. Supplemental Figure 6 stainings are not comparable in terms of intensity, light and contrast.

Referee #2 (Other Remarks):

The triple negative breast cancer (TNBC) lacks targeted therapy. Identifying the critical pathways driving the growth of this type of breast cancers is a high priority in the breast cancer research field. The important role of the Wnt pathway in some of the TNBC has been implicated in several studies published in the past few years. The authors in the manuscript tried to demonstrate that the mouse MMTV-Wnt10B mammary tumor model mimics human TNBC. They further attempted to link the Wnt10B-induced canonical Wnt pathway to HMGA2 expression and the proliferation of cancer stem cells which they believed to be the CD44+ CD24+ cells. While the issue that the authors tried to address is important, the data presented are not convinicng or lack proper controls. Some of the concerns are listed below:

1. The authors concluded that WNT10B was strongly expressed in >60% TNBC based on IHC using a WNT10B antibody from Abcam (page 7 and Supplemental Material and Methods). The authors stated that that was the first time that WNT10B was detected in primary human breast tumors by IHC (page22). There are four antibodies against WNT10B from Abcam: ab70816, ab105860, ab66721, and ab91201 (http://www.abcam.com/index.html?pageconfig=searchresults). Based on the company's website, both ab70816 and ab105860 recognize some unknown proteins based on Western blot, especially at 47kDa and 93kDa regions, making them less specific to WNT10B. The ab66721 work for ICC (immunocytochemistry) but not IHC; and the ab91201 IHC staining signal is located outside the cells in several tissues, which is significantly different from the staining pattern seen in this manuscript. For these reasons, the detail information of the antibody used in this study and the positive/negative controls are critical for evaluating the result. Unfortunately, neigher the specific antibody information nor the positive and negative controls can be found in this manuscript, making it difficult to evaluate the result. Furthermore, based on the Oncomine search, there is no significant difference of the WNT10B expression level between TNBC and non-TNBC. Therefore, the staining status of WNT10B in non-TNBC samples should be presented to show whether WNT10B is specifically expressed in the TNBC. This oncomine analysis should be discussed.

2. The authors claimed that the LacZ+CD44+CD24+ cells in the Wnt10bLacZ mammary tumor are a "stem-cell population" (page 11 and other pages). This claim lacks critical evidence. The limiting dilution transplantation assay is the only accepted method to demonstrate that a specific cell population is a stem cell population. This assay needs the transplantation of both the proposed stem cell population and the non-stem cell population. Only when the proposed stem cell population gives rise to tumor growth at a significantly higher frequecy than the non-stem-cell population, the proposed stem cell population can be concluded to be a stem cell-enriched population. Unfortunately, the authors only transplanted the LacZ+CD44+CD24+ cells without including the non-LacZ+CD44+CD24+ cell transplantation comparison.

3. The authors demonstrated that the Wnt10b-driven tumors are TN tumors by showing ER, PR and Erbb2 negativity using qt-PCR (Fig2 and S3). Because the autors used pregnant mammary gland as a posittive contol, the relatively low mRNA level of ER or PR in the Wnt10b-driven tumors does not necessaryly mean that these tumors are ER/PR negative. IHC-based scoring on these tumors is necessary to evaluate whether these tumors are ER/PR negative. A similar problem is also true for concluding HER2 negativity.

4. The authors tried to demonstrate "nuclear HMGA2 expression is restricted to TNBC as it was not detectable in ER-α+, PR+, Her2+ and TP breast cancer samples (Fig.6B)" (page 19). While they stated that 38 TNBC samples were used to demonstrate that greater more than 80% of them were positive for nuclear HMGA2, they did not show how many ER-α+, PR+, Her2+ and TP breast cancer samples were used for comparison. This is a very important concern because the Oncomine TCGA microarray data show no significant difference of the HMGA2 expression level between the

TNBC and non-TNBC. There are many microarray datasets available in the Oncomine. Instead of chosing the large datasets such as the TCGA breast, the authors chose the relatively small dataset of Richardson to conclude that "HMGA2 is highly and specifically expressed in triple-negative (ER-PR-ErbB2-) human breast cancer". However, even in this dataset, the difference of HMGA2 expression between the TNBC and non-TNBC is not significant. Therefore, the authors' presentation of the analysis of public datasets was misleading.

5. Staining for nucleare β-catenin, axin2, CK6 is not convincing.

6. On page 3, the authors described "These breast cancers are divided into six subtypes that are highly heterogeneous both histologically (Weigelt & Reis-Filho, 2009) and by gene expression profiling (Perou et al, 2000; Sorlie et al, 2003): luminal A, luminal B, ERBB2/HER2, normal breast-like, basal-like and triple-negative (TN)." The terms used in the IHC-based clinical clasification and the microassay-based clasification should not be mixed together.

7. CD61 and Sca1 are not MaSC marker (page 8).

8. The rationale of the comparison of mammosphere-forming frequency between normal mammary gland cells and tumor cells is not clear (page 12).

9. Some of the citations in text and bibliography are not formated well or incomplete.

Referee #3 (Other Remarks):

The manuscript by Wend et al. reports on the role of Wnt signalling in triple negative breast cancer by making use of interesting mouse models and of primary human material. Overall, I found that, although it relies on previous evidence on the role of Wnt in TNBC and as such it is not entirely novel, it contains several advancements in our knowledge of the underlying cellular and molecular mechanisms and in particular on the role played by WNT10B as a ligand and of HMGA2 as a downstream effector. Having said that, the manuscript is excessively long as it encompasses a rather extensive body of experimental evidence presented in a confusing fashion that makes it extremely difficult for the reader to go through. Also, too often statements are based on "data not shown" and the rationale for a number of experiments has not been explained. In view of this, I'd propose major revision of the manuscript along the lines of my specific comments listed here below.

1. Page 6-7. The panel of 14 (German) and 24 (US) primary TNBCs samples is rather limited and, ideally, larger cohorts should be employed to validate the observations on WNT10B expression. Also, the evidence relative to cells earmarked by nuclear beta-catenin localization is central to this manuscript but is, somewhat surprisingly, not presented (data not shown). The IHC images presented in Fig. 1A, especially with Ab's directed against beta-catenin are not convincing and nuclear beta-catenin is not evident. As it is generally known, IHC analysis of beta-catenin subcellular localization is tricky and antigen retrieval protocol based on neutral pH should be preferred. From the images in my possession, strong (overstained?) cytoplasmatic staining is seen which makes it difficult to evaluate nuclear expression. The IF-based evidence for the co-localization of nuclear beta-cat with basal epithelial markers and CD44 (Fig. S1B-C) is also not very convincing and again this might be due to the poor graphic resolution of the figures in my pdf file. Also, CD44 is here mentioned as a "stem cell marker" with no references to back this up. To the best of my knowledge, although there are reports on the use of CD44 to enrich (cancer) stem cells (when combined with other CD markers) in specific tissues, CD44 is more often expressed in progenitor cells.

2. Pages 7-8. I admittedly am not an expert on this but the data relative to WNT10B as a predictor of survival outcome (Fig. 1C-D) do not appear to be very strong. Again, important data to support this concept (LRP6 as a predictor and the specificity of WNT10B for TNBC but not for ER-a-positive breast cancers is stated but not presented (data not shown). Overall, the conclusive paragraph at page 8 contains a number of rather hard conclusions which are not entirely supported by the experimental evidence (also the evidence relative to AXIN2 expression is not particularly convincing (Fig. 1A; also in view of the scarcity of specific Ab's capable of recognizing AXIN2 in IHC) and which

should be tempered.

3. Page 8-9. To analyse the expression of MaSC markers in Wnt10b-driven tumors qt-PCR and IHC (again as data not shown) analysis is employed. However, the markers employed were originally found to enrich for MaSCs primarily by FACS analysis when employed in specific combinations (e.g. CD29hiCD24+). As such, it is difficult to interpret the significance of these results. Instead, I would have expected a thorough FACS analysis of these mouse tumors coupled with limiting dilution transplantation assays in immune-deficient or syngeneic hosts. Also, the sentence on CD44+CD24-/low in human TNBC (page 9) appears as "out of the blue" and not so relevant to this section of the result. In general, these type of comments which entirely rely on previous literature should be confined to the Discussion section.
The transplantation assays performed with Lin-Lacz+ tumour cells have been performed with extremely high number of cells (Fig. S2B-C; >0.5*106) and are not informative relative to their identity as CSCs or tumour-initiating cells. Also in this case, the data relative to Lacz+CD44hiCD24hi transplantation are not shown and it is not clear what the rationale was to prefer this specific combination of CD markers to others previously demonstrated to encompass CSCs in Wnt-driven tumors (e.g. CD29hiCD24+).

4. Page 10. To characterize (quantify?) Lacz+CD44hiCD24hi tumor cells, FACS analysis was employed (Fig. 2D). This part is unclearly written and I had difficulties in understanding what was actually done. The figure shows 3 distinct FACS panels relative to Lin-Lacz+ (tumour) cells (i.e. not Lacz+CD44hiCD24hi as stated in the text) with no indication to what they represent (not even in the legend). From the text, with some guessing, one infers that, from left to right, they show normal mammary gland cells from wild type and a transgenic Wnt10bLacz mice, and tumour cells from Wnt10b-driven tumors. Provided my interpretation is correct, it is not clear what the significance of these analyses is in the absence of transplantation assays (at limiting cell multiplicities) for each of these subpopulations. Once again, the rationale for the choice of CD24 and CD44 instead of other previously established combinations of CD markers is unclear.
In the last paragraph, it is stated that the expression of specific Wnt targets was in fact lower in Lacz+CD44hiCD24hi tumor cells when compared with Lacz+ and bulk tumour cells. This is surprising as it indicates a lower level of Wnt signalling in the alleged CSC population. These data should be confirmed by Wnt reporter assays rather than by evaluation of few downstream targets. This is also partly contradictory with the co-localization of these stem cell markers and nuclear beta-catenin.

5. Page 11. When it comes to Hmga2 expression, it is notable that this is highest in the bulk (unsorted) tumour cells when compared to all other subpopulations. Again, this questions the relationship between Wnt signalling, nuclear beta-cat and cancer stemness, also in relation to Hmga2 expression. Notably, Hmga1 is mostly expressed in Lacz+CD44hiCD24hi tumor cells but this observation is somewhat left unnoticed and is not followed up any further. From this perspective, the conclusive statement of this paragraph according to which Hmga1 and Hmga2 expression is increased in the stem-cell population is true only for the former but not for the latter.

6. Page 12. The mammosphere assays were performed with bulk tumour (and normal) cells and as such are not very informative. Preferably, FACSorted subpopulations of tumour cells should have been employed under controlled experimental conditions (limiting cell multiplicities in matrigel).

7. Page 19. The data relative to the high (>80%) frequency of nuclear HMGA2 expression in TNBCs is again presented as "data not shown". Also, it is said that many of these TNBC were in fact metaplastic breast cancers and as evidence for that H&E staining is presented (Fig. 6A and SA-C). Metaplasia should be confirmed by IHC with specific markers for squamous or other "illegitimate" differentiation lineages. To the best of my capacity to analyse the figures (with the previously mentioned limitation of the poor graphic resolution) the images do not support this.

8. Pages 21-25. In view of my general introductory comments, the lengthy discussion should be shortened considerably.

9. Page 23. "...we also provide evidence that Wnt10b-driven tumors are highly enriched in Lacz+CD44hiCD24hi cells that have cancer-initiating capacity when serially passaged in syngeneic mice (data not shown)". These data are indeed not shown throughout the manuscript and as such the

authors cannot make this claim! Likewise, self-renewal assays (repeated cycles of CSC sorting by FACS, their transplantation at limiting dilution thus demonstrating not only their self-renewal but also their capacity to differentiate and recapitulate the primary tumors) were never formally performed. In this page, self-renewal of CD44+ cells (?) is implied but was never shown. Likewise, in the follow-up of this discussion, it is suggested that CD44+ cells from TNBC tumors may be enriched in CSCs but the evidence for this is limited to immortalized cell lines resembling TNBC and not to primary patient material.
Overall, I believe that the evidence relative to WNT10B and HMGA2 expression and their downstream effectors in mouse and human TNBCs is novel and of general interest. However, when it comes to cancer stemness, its relationship with Wnt signalling, and the identification of a specific subpopulation of TNBC cells the evidence is much weaker and in part simply not shown or not appropriately performed. Given the value of the data on the functional role of to WNT10B and HMGA2 expression in TNBC, I would have preferred a shorter and easier to read manuscript with focus on these two genes, independently from the cancer stem cell aspects which are clearly less developed. Hopefully, my comments will be of help to the authors to improve their interesting manuscript.

1st Revision - authors' response 07 August 2012

(please see next page)

1st Revision - authors' response 07 August 2012

General response to all reviewers:

We would like to thank the reviewers for their critical and insightful reviews of our manuscript. We have made a major revision of the manuscript to address the major concerns raised. We have also removed whole sections from our previously submitted manuscript, based on the recommendation from the reviewers. Additionally, we have worked rigorously to stream line our story and focused on the editing of the MS for easier reading and focused our discussion on how WNT10b regulates HMGA2 and its role on proliferation. We have removed almost 10,000 characters from the text to achieve your recommendations. Please find the point-by-point responses to the critiques.

***** Reviewer's comments *****

-

Referee #1 (Other Remarks):

In this manuscript, Wend et al provide evidence that transgenic murine WNT10B-driven tumors resemble human triple negative breast cancers. The authors suggest that WNT10B signaling activates beta-catenin leading to HMGA2 upregulation and proliferation. They go on to suggest that this observation applies to CD44+ cells, which is one of the known self-renewal markers in mammary epithelial cells. Finally, the authors use a chemical inhibitor of Wnt/B-catenin pathway to validate some of their findings. Identifying mouse models that mimic triple negative breast cancer is of interest given its current absence. The mechanisms that contribute to the triple negative breast cancer phenotype have been the center of much attention since this could be pivotal to tackle this aggressive disease.

This manuscript identifies an interesting model that could serve the study of triple negative breast cancer in the mouse and the description is largely convincing. However, this represents and incremental but not a substantial advance since the mechanistic analysis and the phenotypic explanations are rather confusing, not hypothesis driven and lack of important controls and gaps.

Major Points:

1. The manuscript is a collection of evidences, with important gaps that need to be filled. First, the authors show that when ER negative (not triple negative) breast cancer primary tumors are separate according to WNT10A mRNA levels but not WNT1 these two groups behave differently in terms of relapse (log rank test). It is not clear whether ER negative patients that express WNT10b are TNBC or not. In turn, the authors, based on a small series of IHC analysis, suggest that protein levels exert this function and, in turn, this is specific of triple negative breast cancer. Unfortunately, no statistics are draw. Then the authors describe a transgenic mouse model that express WNT10b. These mice develop mammary tumors that resemble TNBC. This is an interesting observation, however how does WNT10b generate this

phenotype is not clear at all. The authors suggest is via HMGA2, but do not show how. Moreover, they claim that HMGA2 triggers a self-renewal phenotype and more proliferation but they do not show what is the relationship with the TNBC. Overall, the message is not clear, the reader gets confused and no mechanistic link between WNT10b, signaling, expression of HMG2A and contribution of HMG2A to TNBC phenotype is observed. The authors need to make an important effort to streamline these points, connect them and provide data that fills the gaps.

1) "It is not clear whether ER negative patients that express WNT10b are TNBC or not. In turn, the authors, based on a small series of IHC analysis, suggest that protein levels exert this function and, in turn, this is specific of triple negative breast cancer. Unfortunately, no statistics are draw."

Point by point response: We have a new Figure 1 which now allows for the assessment of the WNT10B antibody on a set of TMAs. We have quantitated the results. We show new survival curves from basal-like cancer and illustrate that WNT10B has predictive value (KM-plotter). We have also finished our analysis on 59 TNBC samples illustrating that WNT10B has predictive value for two categories: tumor size and nuclear grade with significant statistics (p=0.021 and p=0.025).

i) "Then the authors describe a transgenic mouse model that express WNT10b. These mice develop mammary tumors that resemble TNBC. This is an interesting observation, however how does WNT10b generate this phenotype is not clear at all. The authors suggest is via HMGA2, but do not show how".

Point by point response: We illustrate that Wnt10b^{LacZ}-driven tumors are devoid of ER, PR and HER2 protein expression connecting our model better to TNBC in the text. We have now addressed mechanistic questions on how we think HMGA2 contributes to our model and Figure 2-3 show new data. i) We illustrate that HMGA2 expression is lost in Wnt10B-knockout (*Wnt10b^{KO}*) mice in early embryogenesis in the mammary anlagen ii) We illustrate evidence that beta-catenin directly ChIP's to the HMAG2 promoter and our new biochemical data show that it requires CBP interactions. iii) Silencing of HMGA2 leads to a block in proliferation in both mouse mammary gland cell lines and a tumor-derived cell line.

2) "Moreover, they claim that HMGA2 triggers a self-renewal phenotype and more proliferation but they do not show what is the relationship with the TNBC…"

Point by point response: Based on the critique we have removed much of the self-renewal references from the MS as we realized that we lack experimental support. We now focus on "self-renewal" to compare cells from wildtype *vs. Wnt10b^{KO} vs. WNt10b^{LacZ}* mice for the capacity to generate mammospheres over a 30 days period (Fig. 2 F-G).

2. The authors' state (page 4, third paragraph) based on the literature that 50% of breast cancers have nuclear b-catenin. However, when they analyze their patient sample series, they restrict WNT10b to a small subset of TNBC samples. They go on and attribute to tumors that express WNT10b TNBC properties. However, if 50% of breast cancer tumors have nuclear b-catenin in the nucleus (meaning Wnt signaling is ongoing) and >60% of breast cancer patients are ER+, it is unlikely that WNT10b through Wnt signaling leads to TNBC in a representative cohort of breast cancer patients. Indeed, this suggests that Wnt signaling (nuclear b-catenin) is not responsible for the TNBC phenotype and that other things,

possibly downstream of WNT10b, are more relevant to establish such phenotype. The key point is what is responsible for TNBC independently of Wnt-signaling. A question that the authors do not address. Moreover, this point questions their transgenic mice model. Thus, prompting for the request to induce MMTV-WNT10b driven expression in a different genetic background to confirm their observation.

Point by point response: We thank the reviewer for the critique. We understand the controversy over the relevance of beta-catenin in breast cancer (why is not always nuclear).

a) We have corrected the paragraph in question and changed it to "… ß-catenin is upregulated in more than 50% of breast cancer cases (Cowin et al, 2005)…" and removed "nuclear". We apologize for the over statement; it was an error when editing and it now reflects what is known in the field.

b) We hope that new data Fig. 1A-B and Supplemental Fig. S1A-B clarifies our results and statement. We have attempted to show the correlation of WNT10B in similar regions as that of beta-catenin and have conducted both *in situ* hybridization (ISH) and immunohistochemistry (IHC) for AXIN2, a well-known beta-catenin direct target gene. Furthermore, we illustrate that we have active gene expression of the Wnt pathway in our TNBC cases. This suggests that even though beta-catenin does not always look nuclear it can still activate transcription of its target genes (e.g. Axin2). We have also added new data on nuclear β-catenin that corresponds to AXIN2 expression (Suppl. Fig. S1C, D). Also illustrate additional β-catenin IHC supplemental Fig. S8.

c) We could not generate a new genetic mouse model (e.g. in a different genetic background) in the time for the revision of this manuscript. But we did conduct IHC for ER-α, PR and HER2 $Wnt10b^{LacZ}$-induced tumors and they were negative illustrating their TN phenotype. We now also provide positive control staining for tissues such as mouse uterus and ErbB2 tumor.

3. As highlighted before, the authors should include data referring to the additional 24 TNBC samples from the cohorts from LA and Chicago. Moreover, statistics depicting the correlation between nuclear b-catenin and Wnt10b should be draw. Finally, a comparison with other breast cancer tumors subtypes is also necessary. It must be shown whether this correlation is unique of TNBC or it also occurs in other breast cancer subtypes (plus statistics).

Point by point response: We thank the reviewer for the valuable critique. We have addressed the WNT10B comparison to tissue from other breast cancer subtypes by analyzing 125 samples from commercially available tissue microarrays (TMA) and illustrate several samples for TN, ER^+ PR^+ and $Her2^+$ breast tumors. Our pathologists have quantified the results (Fig. 1B). We provide a new table for the tumors (Supplemental Table 1) and depict the statistics in supplemental Fig S7.

Reviewer comments: "Moreover, statistics depicting the correlation between nuclear b-catenin and Wnt10b should be draw"

Point by point response: We thank you for this valuable recommendation. We would like to respond to this point by provide data only for the reviewers eyes by showing the data but not including in the manuscript as it is planned to be used for another ongoing project. (Data not shown in this Peer Review Process file.)

4. Again, association between CD44 and TNBC markers (including nuclear b-catenin) lacks statistics. Significance is missing and without it no claims can be made. Similarly, this applies for WNT10b and AXIN2.

Point by point response: We have removed all data for CD44 and nuclear b-catenin co-staining to stream line and shorten the MS. We apologize but we did not have time to conduct statistics on AXIN2 because we need to cover over 180 samples to do this. We lacked the time and resources to meet this critique. But we did conduct ISH and IHC for AXIN2 in over 14 samples (i.e. German cohort group) and the results held.

(Continued on next page.)

5. Page 8, second paragraph. The authors state that WNT10b expression was validated to be high in TNBC. The authors should stick with the data. The data depicted indicates that WNT10b is relevant in ER negative breast cancer but not in TNBC (the latter is included in the earlier). Finally, since the authors claim prediction of risk, COX proportional hazard analysis instead of Log Rank test should be performed.

Point by point response: We have addressed the reviewer's concerns as follows:

i) During the revision we have conducted a completely new analysis of TNBC patient material showing the significant correlation between high WNT10B expression and poor clinical outcome, such as tumor size and nuclear grade (Fig. 1D).

ii) We have added the basal-like data from the KM-plotter showing that WNT10B is relevant (Fig. 1E) but in contrast WNT1 is not.

iii) The hazard ratio (and the corresponding 95% confidence interval) was in fact estimated using Cox's proportional hazard model. The hazard ratios are depicted in the KM survival curves (HR, upper right in the diagram). The score test of the proportional hazard model is equivalent to the log-rank test.

iv) We also added the following in the text: "Remarkably, *WNT10B* was able to predict survival outcome, time-to-event curves analyzed by Cox proportional hazards regression analysis generated a hazard ratio (HR) of 1.38 ($p=0.038$)" to paragraph 2 page 8.

7. The authors claim that MMTV-WNT10b mice BC tumors are enriched for cells with high self-renewal potential. Surprisingly, they define tumor-initiating cells (page 9, 2nd paragraph) as CD44high, CD24low. However, through their manuscript they only focus on CD44high, CD24 high. How come? A very convincing explanation must be provided. Clearly, their statement in page 22 and 23 of the discussion does not help, 2 out of the 3 arguments provided highlight the importance of CD24low for aggressiveness and stemnes.

Point by point response: We thank the reviewer for these thoughts and we agree that this section still requires much more work and it is still very preliminary at best to be published. Therefore, we have completely removed the figure and all references to CD44 and CD24 markers and stemness.

8. The authors claim that the self-renewal marker HMGA2 is expressed and provides self-renewal functions. However, in figure 3b it can be observed how unsorted cells expressed higher levels of HMGA2 than tumor initiating cells. Thus, the stem cell population does not experience the higher induction of this gene. These results suggest that this protein might be necessary but not sufficient to explain this WNT10b driven phenotype. On top of that, how does WNT10b regulate HMGA2?

Point by point response: We have completely revised Figure 3 and focused our comments and interpretations to the mechanism by which WNT10b regulates Hmga2.

i) We removed references in the text and figures about HMGA2 as a self-renewal marker.

ii) We illustrate that WNT10b can induce *Hmga2* expression in a normal mouse cell line NuMG. More importantly we provide the mechanism of action to be beta-catenin/CBP dependent (Fig. 3B-C) and that cell proliferation is affected after shRNA-mediated knockdown of Hmga2 in the presence of WNT10B (Suppl. Fig. S3).

iii) We have silenced Hmga2 expression in tumor-derived cell lines and block proliferation.
iv) We have inhibited sphere formation of primary tumor cells with both Wnt-ligand inhibitor (Wnt-C59) and ICG-001 to disrupt nuclear beta-catenin activity. (Fig. 3 H,J).

9. Inhibitors are very dangerous toys. They are accompanied with uncontrolled side effects. Thus, the authors must use and independent compound that targets the same principle to ensure robustness. Alternative, they could use a b-catenin shorthairpin construct to validate some of their key experiments.

Point by point response: We agree and thank the reviewer for this comment.

We have now addressed this concern several times in the MS by also testing our model to exposure to Wnt-C59 (a *Porcupine* inhibitor like IWSP 2, 3 and 4), which blocks secretion of Wnt ligands. We have compared the effects of Wnt-C59 directly to ICG-001 (Fig. 3J and Fig. 4F). The results measured for Wnt-C59 are very similar to those induced by ICG-001.

10. Page 16, first paragraph. In figure 5 the authors use ICG-001 to block b-catenin signaling and test downregulation of HMGA2, CyclinA2 etc... The question is: what does happen in MCF710b cells? This is of particular interest given in these cells all nuclear b-catenin signaling is, in principle, generated by WNT10b.

Point by point response: We have addressed this concern in Supplemental Fig. S4B-C. To the text we added the following

i) Moreover, HMGA2 is not responsive to 17β-estradiol (E2) treatment of parental MCF7 cells or in MCF7-10b cells; MCF7-10b cells are still responsive to E2 treatment by upregulation of *XBP1* and *pS2* and express known canonical *Wnt*-signaling target genes, such as *c-myc*, *CCND1* and *DKK1* (Supplemental Fig. S4A). Importantly, treatment of MCF7-10b cells with ICG-001 blocks proliferation and down regulates HMGA2 expression (Supplemental Fig. S4B-C and page 15, 1st paragraph).

11. The authors used the MDA-MB-231 cells to test whether ICG-100 cause an induction of INK4 locus CDK inhibitors. These results are puzzling, particularly because there are several reports in the literature claiming that these breast cancer cells are INK4 locus deleted (i.e Bisonga et al Cancer Genetics and Cytogenetics 125 (2001) 131-138 and others). This locus includes p16, p19 and p15. Thus it is very strange that the authors do not observe activation of p16 and p19 but observe activation of p15. Given the literature reports, the authors must verify that their MDA-MB231 cells have an intact INK4 locus (including INK4b).

Point by point response: We strongly regret this over site and thank the reviewer for this very important observation. We have removed all references to INK locus from the MS. We have gone back to each experiment and discovered where the probable mistakes occurred in switching out the inappropriate cell line. We are fortunate that this was solely for that INK locus western results and all of the qt-PCR conducted in that context. We purchased some variants of MDA-MB cell lines from the ATCC to ensure we had the right phenotype with ICG-001 treatment (Fig. S4F) and we added the following to the text of

the manuscript and repeated many of the same experiments to ensure that we have the same results as the ATCC cell lines.

> "We further validated the effects of ICG-001 on various subtypes of TNBC cells lines, we purchased commercially from ATCC to ensure authenticity of the cell lines. ICG-001 treatment down regulated HMGA2 and two of its downstream targets CCNA2 and CCNB2 and BIRC5, which is a known direct target of ICG-001 in a subset of MDA-MB derived cell lines (Supplemental Fig. S4F)

12. To determine if HMGA2 is necessary and sufficient for proliferation of MDA-231 cells downstream of WNT10b, the authors should test if WNT10b exogenous expression effect is completely abolished by shHMGA2.

Point by point response: We thank the reviewer for these insightful comments and we think that the requested experiments have strengthened our manuscript substantially.

i) We added two new sections to Fig. 5 (E,F) to address the above comment.
ii) We added the following to the text of the MS: Page 19, 2nd paragraph"

"To test our model for the order of action of genes in a regulatory hierarchy that governs the Wnt10b/beta-catenin signaling pathway, we designed an epistatic functional assay for *WNT10B*. We transfected MDA-MB-231 cells with pcDNA3-GFP and pcDNA3-WNT10B vectors, cells were harvested and qt-PCR was conducted for *WNT10B*, *HMGA2* and *CCNA2* (Fig. 5E). Concurrently, we repeated the previously mentioned transfection with our MDA-MB-231-*shHMGA2* silenced verified cell lines (Fig. 5F). The results illustrate that WNT10B induced the expression of *HMGA2* and is its known downstream target *CCNA2* in control cells. In contrast, cell lines silenced for *HMGA2* can still express *WNT10B* but the expression of *HMGA2* and its downstream target *CCNA2* is lost. The above experiment supports the epistatic activity of WNT10B on both *HMGA2* and *CCNA2* gene expression in triple-negative MDA-MB-231 cells."

13. Given the same sample set was used to evaluate the differences in disease-free survival between groups that express low and high WNT10b and HMG2A, a correlation between the expression of both genes and its significance should be draw as this is pivotal to the author's claims.

Point by point response: We have utilized a Kendal Tau-b correlation coefficient analysis for the correlation between the two markers in our own 55 TNBC samples and as well for both beta-catenin and BMI-1 for another ongoing publication. The results show that concurrence occurs in the paired analysis between the 4 markers. The strongest correlation is between HMGA2 and BMI1 (0.80386) while WNT10b has an identical coefficient of correlation (0.79497) with both beta-catenin and HMGA2 (data not discussed in the manuscript). Please see above to reviewer #1 for the detail data for this point by point response for the eyes of the reviewers only.

© EMBO

Supplemental Figure 6 staining's not comparable in terms of intensity, light and contrast.

Point by point response: We have corrected this with Photoshop.

Referee #2 (Other Remarks):

The triple negative breast cancer (TNBC) lacks targeted therapy. Identifying the critical pathways driving the growth of this type of breast cancers is a high priority in the breast cancer research field. The important role of the Wnt pathway in some of the TNBC has been implicated in several studies published in the past few years. The authors in the manuscript tried to demonstrate that the mouse MMTV-Wnt10B mammary tumor model mimics human TNBC. They further attempted to link the Wnt10B-induced canonical Wnt pathway to HMGA2 expression and the proliferation of cancer stem cells which they believed to be the CD44+ CD24+ cells. While the issue that the authors tried to address is important, the data presented are not convincing or lack proper controls. Some of the concerns are listed below:

1. The authors concluded that WNT10B was strongly expressed in >60% TNBC based on IHC using a WNT10B antibody from Abcam (page 7 and Supplemental Material and Methods). The authors stated that that was the first time that WNT10B was detected in primary human breast tumors by IHC (page22). There are four antibodies against WNT10B from Abcam: ab70816, ab105860, ab66721, and ab91201 (http://www.abcam.com/index.html?pageconfig=searchresults). Based on the company's website, both ab70816 and ab105860 recognize some unknown proteins based on Western blot, especially at 47kDa and 93kDa regions, making them less specific to WNT10B. The ab66721 work for ICC (immunocytochemistry) but not IHC; and the ab91201 IHC staining signal is located outside the cells in several tissues, which is significantly different from the staining pattern seen in this manuscript. For these reasons, the detail information of the antibody used in this study and the positive/negative controls are critical for evaluating the result. Unfortunately, neither the specific antibody information nor the positive and negative controls can be found in this manuscript, making it difficult to evaluate the result. Furthermore, based on the Oncomine search, there is no significant difference of the WNT10B expression level between TNBC and non-TNBC. Therefore, the staining status of WNT10B in non-TNBC samples should be presented to show whether WNT10B is specifically expressed in the TNBC. This oncomine analysis should be discussed.

Point by point response: We thank the reviewer for the thorough analysis of the WNT10B antibody and we completely agree with the concerns. We have searched exhaustively for WNT10B antibodies over the years and we are excited about a WNT10B antibody from Abcam, catalog number Ab91201. We apologize for the oversight of not listing it earlier. We have done extensive serial dilutions with the antibody to optimize specific-labeling to help address this concern. We purchased TMAs for the control requested for IHC. Illustrated in Fig. 1A are the majority of the TMA's signature that is: negative for both ER^+ and PR^+ and some positive in the $HER2^+$ tumors. The few TN in the samples had a gradient of positive expression but all were well above the negative controls.

© EMBO

2. The authors claimed that the LacZ+CD44+CD24+ cells in the Wnt10bLacZ mammary tumor are a "stem-cell population" (page 11 and other pages). This claim lacks critical evidence. The limiting dilution transplantation assay is the only accepted method to demonstrate that a specific cell population is a stem cell population. This assay needs the transplantation of both the proposed stem cell population and the non-stem cell population. Only when the proposed stem cell population gives rise to tumor growth at a significantly higher frequecy than the non-stem-cell population, the proposed stem cell population can be concluded to be a stem cell-enriched population. Unfortunately, the authors only transplanted the LacZ+CD44+CD24+ cells without including the non-LacZ+CD44+CD24+ cell transplantation comparison.

Point by point response: We thank the reviewer for these thoughts and we agree. We have removed all references dealing with this topic from the manuscript. This area still requires more work in progress. We have only utilized FACS-LacZ sorted cells from *Wnt10b*^{LacZ}-induced primary tumors for *in vitro* assays for treatment with Wnt inhibitors.

3. The authors demonstrated that the Wnt10b-driven tumors are TN tumors by showing ER, PR and Erbb2 negativity using qt-PCR (Fig2 and S3). Because the autors used pregnant mammary gland as a posittive contol, the relatively low mRNA level of ER or PR in the Wnt10b-driven tumors does not necessaryly mean that these tumors are ER/PR negative. IHC-based scoring on these tumors is necessary to evaluate whether these tumors are ER/PR negative. A similar problem is also true for concluding HER2 negativity.

Point by point response: We now provide IHC-based evidence for the absence of ER-alpha, PR, and HER2 expression in *Wnt10b*^{LacZ}-driven tumors (Fig. 2A). We utilized wildtype mouse uterus tissue for controlling the ER-alpha and PR expression levels and the MMTV- ErbB2^{TG} tumor for HER2 expression control.

4. The authors tried to demonstrate "nuclear HMGA2 expression is restricted to TNBC as it was not detectable in ER-α+, PR+, Her2+ and TP breast cancer samples (Fig.6B)" (page 19). While they stated that 38 TNBC samples were used to demonstrate that greater more than 80% of them were positive for nuclear HMGA2, they did not show how many ER-α+, PR+, Her2+ and TP breast cancer samples were used for comparison. This is a very important concern because the Oncomine TCGA microarray data show no significant difference of the HMGA2 expression level between the TNBC and non-TNBC. There are many microarray datasets available in the Oncomine. Instead of chosing the large datasets such as the TCGA breast, the authors chose the relatively small dataset of Richardson to conclude that "HMGA2 is highly and specifically expressed in triple-negative (ER-PR-ErbB2-) human breast cancer". However, even in this dataset, the difference of HMGA2 expression between the TNBC and non-TNBC is not significant. Therefore, the authors' presentation of the analysis of public datasets was misleading.

Point by point response: We apologize for these concerns and we did not attempt to mislead on purpose. We have removed all references for the Oncomine data and will in the future better understand its usefulness to present new arguments. We now provide new evidence from our own collection of

TNBC and from the KM-plotter, which shows that HMAG2 expression has clinical relevance for the basal-like breast cancer subtype (Fig. 6C , D).

5. Stainings for nuclear beta-catenin, axin2, CK6 is not convincing.

Point by point response: Similar concerns were also mentioned by another reviewer. We thank the reviewers for this critique. We understand the controversy over the relevance of beta-catenin in breast cancer, why is not always nuclear, if it is relevant? To address this we:

i) We have removed all of the co-staining from immunofluorescence experiments with beta-catenin, CK5, CK6, CK14 or CD44 from the manuscript.

ii) 3 new AXIN2 IHC stainings (Suppl. Fig. S1B) from similar sequential tumor sections as in Fig. 1C were conducted. Taken together, we feel (Lugli et al, 2007)both the IHC and ISH for AXIN2 present evidence for active canonical Wnt signaling in the TNBC samples.

iii) We undertook a set of experiments to alter the pH value of our samples and repeated IHC for beta-catenin utilizing further TN samples. In the revised manuscript we present evidence for both nuclear (white arrows) and cytoplasmic (red arrows) beta-catenin (Fig. S1C). Many cells are adjacent to each other that have both phenotypes. Published data for colorectal cancer and other studies on TNBC have shown that the overall content of nuclear and increased cytoplasmic beta-catenin varies between 5-80%in the tumor tissues (Geyer et al, 2011; Khramtsov et al, 2010; Lugli et al, 2007; Martensson et al, 2007; Wong et al, 2003) Moreover, the distribution of nuclear beta-catenin throughout the tumor tissue is very heterogenous and often found at the invasive tumor front (Brabletz et al, 2005; Fodde & Brabletz, 2007) Our IHC staining results for beta-catenin confirm those observations (see also new Suppl. Fig.S8) and verify that nuclear beta-catenin can be detected in our TNBC patient samples verifying activated Wnt/beta-catenin signaling in these tumors. One of the sections corresponds to the IHC for AXIN2, which was illustrated above in ii (Fig. S1B).

6. On page 3, the authors described "These breast cancers are divided into six subtypes that are highly heterogeneous both histologically (Weigelt & Reis-Filho, 2009) and by gene expression profiling (Perou et al, 2000; Sorlie et al, 2003)luminal A, luminal B, ERBB2/HER2, normal breast-like, basal-like and triple-negative (TN)." The terms used in the IHC-based clinical classification and the micro assay-based classification should not be mixed together.

Point by point response: We thank the reviewer for these thoughts and we agree that caution of terms must be made clearer in our manuscript. We have added the following text to pages 6-7:

“ It is important to note that the molecular, clinical and pathological profiles of basal-like and TNBC are similar as they overlap by 60-90% (Al Tamimi et al, 2010); the terms are often interchangeable but they are not identical in their gene expression profiles leading to at least 6-8 complex subtypes (Perou et al, 2000). So for this manuscript the term basal-like will be utilized when referencing microarray data (i.e.

mRNA ERα$^-$, PR$^-$ and HER2$^-$) and the term TNBC will reference to analysis by pathologist who subsequently classified the tumors as ERα$^-$, PR$^-$, HER2$^-$ by IHC. Triple-negative (TN) will be utilized to describe either basal-like and TNBC".

7. CD61 and Sca1 are not MaSC marker (page 8).

Point by point response: We have removed all of the stem cell data and discussions from the manuscript.

8. The rationale of the comparison of mammosphere-forming frequency between normal mammary gland cells and tumor cells is not clear (page 12).

Point by point response: We have modified this section and have made changes to better reflect our rationale.

i) To explain our rationale better, our new data illustrates that HMGA2 is lost in the Wnt10b^{KO} mouse (Fig. 2E) in the early embryo during mammogenesis. We then illustrate our mammosphere-forming frequency to test between gain-of-function cells (*Wnt10b*^{LacZ}-derived tumor cells) and loss-of-function cells (*Wnt10b*^{KO}-derived mammary cells) for "self-renewal" (Fig. 2F) and that tumor spheres correlate with expression of Hmga2. Finally, we utilize the mammosphere-forming assay again using primary tumor cells (Fig. 3H-J) to block their formation with the Wnt-inhibitors ICG-001 and Wnt-C59.

9. Some of the citations in text and bibliography are not formatted well or incomplete.

Point by point response: We apologize for the oversights and have updated our bibliography to correct this.

Referee #3 (Other Remarks):

The manuscript by Wend et al. reports on the role of Wnt signalling in triple negative breast cancer by making use of interesting mouse models and of primary human material. Overall, I found that, although it relies on previous evidence on the role of Wnt in TNBC and as such it is not entirely novel, it contains several advancements in our knowledge of the underlying cellular and molecular mechanisms and in particular on the role played by WNT10B as a ligand and of HMGA2 as a downstream effector. Having said that, the manuscript is excessively long as it encompasses a rather extensive body of experimental evidence presented in a confusing fashion that makes it extremely difficult for the reader to go through. Also, too often statements are based on "data not shown" and the rationale for a number of experiments has not been explained. In view of this, I'd propose major revision of the manuscript along the lines of my specific comments listed here below.

1. Page 6-7. The panel of 14 (German) and 24 (US) primary TNBCs samples is rather limited and, ideally, larger cohorts should be employed to validate the observations on WNT10B expression. Also, the evidence relative to cells earmarked by nuclear beta-catenin localization is central to this manuscript but is, somewhat surprisingly, not presented (data not shown). The IHC images presented in Fig. 1A, especially with Ab's directed against beta-catenin are not convincing and nuclear beta-catenin is not evident. As it is generally known, IHC analysis of beta-catenin subcellular localization is tricky and antigen retrieval protocol based on neutral pH should be preferred. From the images in my possession, strong (overstained?) cytoplasmatic staining is seen which makes it difficult to evaluate nuclear expression. The IF-based evidence for the co-localization of nuclear beta-cat with basal epithelial markers and CD44 (Fig. S1B-C) is also not very convincing and again this might be due to the poor graphic resolution of the figures in my pdf file. Also, CD44 is here mentioned as a "stem cell marker" with no references to back this up. To the best of my knowledge, although there are reports on the use of CD44 to enrich (cancer) stem cells (when combined with other CD markers) in specific tissues, CD44 is more often expressed in progenitor cells.

Point by point response: We thank the reviewer for this critique. We understand the controversy over the relevance of beta-catenin in breast cancer, why is not always nuclear, if it is relevant? We have worked on this and have improved the nuclear staining for beta-catenin in our TNBC samples.

i) We have added new data on nuclear beta-catenin and AXIN2 expression (Suppl. Fig. S1C,D) showing subcellular (red arrowhead) and nuclear (white arrowhead) localization.
ii) We have removed all data from CD44, CK5, CK6 and CK14 from the manuscript. We have focused the revised manuscript on the question how Wnt10b mechanistically regulates HMGA2 and the role of HMGA2 on proliferation.
iii) We have validated the expression of WNT10B in multiple control breast cancer subtypes (see below).

2. Pages 7-8. I admittedly am not an expert on this but the data relative to WNT10B as a predictor of survival outcome (Fig. 1C-D) do not appear to be very strong. Again, important data to support this concept (LRP6 as a predictor and the specificity of WNT10B for TNBC but not for ER-a-positive breast cancers is stated but not presented (data not shown). Overall, the conclusive paragraph at page 8 contains a number of rather hard conclusions which are not entirely supported by the experimental evidence (also the evidence relative to AXIN2 expression is not particularly convincing (Fig. 1A; also in view of the scarcity of specific Ab's capable of recognizing AXIN2 in IHC) and which should be tempered.

Point by point response: We thank you for your critique and have made major changes to address the concerns.

i) IHC for WNT10B expression has now been conducted on 125 samples from TMA analysis with multiple breast cancer subtypes to better assess expression (Fig. 1A,B).
ii) We have increased our own sample collection to include 31 TNBC with complete clinical data and 28 with partial clinic data and illustrate the clinical relevance for WNT10B (Fig. 1C).

iii) We have identified a basal-like (i.e. TN) data base from KM-plotter illustrating that WNT10B can have predictive value.

iv) We provide new AXIN2 IHC in TNBC. We have tempered our argumentation of our results throughout the manuscript.

3. Page 8-9. To analyse the expression of MaSC markers in Wnt10b-driven tumors qt-PCR and IHC (again as data not shown) analysis is employed. However, the markers employed were originally found to enrich for MaSCs primarily by FACS analysis when employed in specific combinations (e.g.CD29hiCD24+). As such, it is difficult to interpret the significance of these results. Instead, I would have expected a thorough FACS analysis of these mouse tumors coupled with limiting dilution transplantation assays in immune-deficient or syngeneic hosts. Also, the sentence on CD44+CD24-/low in human TNBC (page 9) appears as "out of the blue" and not so relevant to this section of the result. In general, these type of comments which entirely rely on previous literature should be confined to the Discussion section. The transplantation assays performed with Lin-Lacz+ tumour cells have been performed with extremely high number of cells (Fig. S2B-C; >0.5*106) and are not informative relative to their identity as CSCs or tumour-initiating cells. Also in this case, the data relative to Lacz+CD44hiCD24hi transplantation are not shown and it is not clear what the rationale was to prefer this specific combination of CD markers to.

Point by point response: We thank you for your critique and have made major changes to address the concerns. For this whole section on MaSC markers and FACS analysis of CD44CD24 tumor cells we completely agree with you and the other reviewers. We do not have complete limiting dilution assays to argue for a CSC phenotype. All data dealing with this topic have been removed from the revised manuscript.

4. Page 10. To characterize (quantify?) Lacz+CD44hiCD24hi tumor cells, FACS analysis was employed (Fig. 2D). This part is unclearly written and I had difficulties in understanding what was actually done. The figure shows 3 distinct FACS panels relative to Lin-Lacz+ (tumour) cells (i.e. not Lacz+CD44hiCD24hi as stated in the text) with no indication to what they represent (not even in the legend). From the text, with some guessing, one infers that, from left to right, they show normal mammary gland cells from wild type and a transgenic Wnt10bLacz mice, and tumour cells from Wnt10b-driven tumors. Provided my interpretation is correct, it is not clear what the significance of these analyses is in the absence of transplantation assays (at limiting cell multiplicities) for each of these subpopulations. Once again, the rationale for the choice of CD24 and CD44 instead of other previously established combinations of CD markers is unclear. In the last paragraph, it is stated that the expression of specific Wnt targets was in fact lower in Lacz+CD44hiCD24hi tumor cells when compared with Lacz+ and bulk tumour cells. This is surprising as it indicates a lower level of Wnt signalling in the alleged CSC population. These data should be confirmed by Wnt reporter assays rather than by evaluation of few downstream targets. This is also partly contradictory with the co-localization of these stem cell markers and nuclear beta-catenin.

Point by point response: We thank you for your critique and have made major changes to address the concerns. As previously stated we have removed the whole bulk of data and discussion on the tumorgenic cell in our model. We have conducted extensive analysis in our tumor model for the known mammary

gland associated markers (CD44, CD24, CD29 and CD49f) in the $LacZ^+$ cells and they are all present with complex interaction that are not well characterized yet. So we removed this and focus the manuscript on Wnt10b regulation of HMGA2 and its role on proliferation.

5. Page 11. When it comes to Hmga2 expression, it is notable that this is highest in the bulk (unsorted) tumour cells when compared to all other subpopulations. Again, this questions the relationship between Wnt signalling, nuclear beta-cat and cancer stemness, also in relation to Hmga2 expression. Notably, Hmga1 is mostly expressed in Lacz+CD44hiCD24hi tumor cells but this observation is somewhat left unnoticed and is not followed up any further. From this perspective, the conclusive statement of this paragraph according to which Hmga1 and Hmga2 expression is increased in the stem-cell population is true only for the former but not for the latter.

Point by point response: We completely agree with the critique and have made a major change in our experimental lineage-dump strategy to address the concerns.

i) We have used a new kit from Stem Cell Technologies to improve our linage-dumping (MG Stem Cell Enrichment cat# 19757) and repeated the qt-PCR for Hmga2 (i.e. Lin^- $LacZ^+$ cells) (Fig. 2C). The results illustrate that $LacZ^+$ cells express as much Hmga2 as the sorted primary tumor cells and it is about 2-fold over that of Hmga1 in the same cells. We also compared the above to virgin mammary gland and ErbB2 tumors.

ii) We have new data that HMGA2 levels are decreased in the $Wnt10b^{KO}$ mouse during the early stages of mammogenesis (Fig. 1E).

6. Page 12. The mammosphere assays were performed with bulk tumour (and normal) cells and as such are not very informative. Preferably, FACSorted subpopulations of tumour cells should have been employed under controlled experimental conditions (limiting cell multiplicities in matrigel).

Point by point response: We have repeated some of the mammosphere assays to make more informative insight into the mechanism for maintenance of the spheres. We FACS sorted (Lin^-LacZ^+) (Fig. 3H,I) and exposed them to the Wnt–inhibitors ICG-001 and Wnt-C59. We illustrate that sphere formation requires both upstream Wnt-ligand and downstream beta-catenin signaling.

7. Page 19. The data relative to the high (>80%) frequency of nuclear HMGA2 expression in TNBCs is again presented as "data not shown". Also, it is said that many of these TNBC were in fact metaplastic breast cancers and as evidence for that H&E staining is presented (Fig. 6A and SA-C). Metaplasia should be confirmed by IHC with specific markers for squamous or other "illegitimate" differentiation lineages. To the best of my capacity to analyse the figures (with the previously mentioned limitation of the poor graphic resolution) the images do not support this.

Point by point response: We have toned down the use of metaplastic breast cancer because we did not have sufficient time to provide evidence by specific markers. We utilize the terminology based on the report of the pathologists who scored and helped us analyzing the data. They also utilize the images from the H&E staining that phenotypically illustrates differentiated cells that look chondrocyte-like with large nuclei (Fig. 1A and Suppl. Fig. S1A-C).

8. Pages 21-25. In view of my general introductory comments, the lengthy discussion should be shortened considerably.

Point by point response: We thank the reviewer for these comments and we completely agree.

i) We have made a major revision to the manuscript based on the recommendations from several of the reviewers to shorten results, discussions and data presentation.
ii) The discussion has been shortened considerably.

9. Page 23. "...we also provide evidence that Wnt10b-driven tumors are highly enriched in Lacz+CD44hiCD24hi cells that have cancer-initiating capacity when serially passaged in syngeneic mice (data not shown)". These data are indeed not shown throughout the manuscript and as such the authors cannot make this claim! Likewise, self-renewal assays (repeated cycles of CSC sorting by FACS, their transplantation at limiting dilution thus demonstrating not only their self-renewal but also their capacity to differentiate and recapitulate the primary tumors) were never formally performed. In this page, self-renewal of CD44+ cells (?) is implied but was never shown. Likewise, in the follow-up of this discussion, it is suggested that CD44+ cells from TNBC tumors may be enriched in CSCs but the evidence for this is limited to immortalized cell lines resembling TNBC and not to primary patient material.

Point by point response: We thank the reviewer for these comments and we completely agree.

i) We have completely removed all the data from CD44CD24 cell- based self-renewal and cancer initiation because of the lack of convincing data.

Overall, I believe that the evidence relative to WNT10B and HMGA2 expression and their downstream effectors in mouse and human TNBCs is novel and of general interest. However, when it comes to cancer stemness, its relationship with Wnt signalling, and the identification of a specific subpopulation of TNBC cells the evidence is much weaker and in part simply not shown or not appropriately performed. Given the value of the data on the functional role of to WNT10B and HMGA2 expression in TNBC, I would have preferred a shorter and easier to read manuscript with focus on these two genes, independently from the cancer stem cell aspects which are clearly less developed. Hopefully, my comments will be of help to the authors to improve their interesting manuscript.

Point by point response:

i) As suggested by the reviewer, we have now focused the manuscript on the functional role of WNT10B and HMGA2.
ii) We provide mechanistic data (Fig 3 & 5).
iii) We provide relevant data showing that WNT10B and HMGA2 expression have predictive value for TNBC (Fig. 1 &6).

iv) We used another Wnt-inhibitor (Wnt-C59) that block Wnt-ligand secretion.
v) We have dramatically reduced the text.

REFERENCES:

Brabletz T, Hlubek F, Spaderna S, Schmalhofer O, Hiendlmeyer E, Jung A, Kirchner T (2005) Invasion and metastasis in colorectal cancer: epithelial-mesenchymal transition, mesenchymal-epithelial transition, stem cells and beta-catenin. *Cells Tissues Organs* **179:** 56-65

Fodde R, Brabletz T (2007) Wnt/beta-catenin signaling in cancer stemness and malignant behavior. *Curr Opin Cell Biol* **19:** 150-158

Geyer FC, Lacroix-Triki M, Savage K, Arnedos M, Lambros MB, MacKay A, Natrajan R, Reis-Filho JS (2011) beta-Catenin pathway activation in breast cancer is associated with triple-negative phenotype but not with CTNNB1 mutation. *Mod Pathol* **24:** 209-231

Khramtsov AI, Khramtsova GF, Tretiakova M, Huo D, Olopade OI, Goss KH (2010) Wnt/beta-catenin pathway activation is enriched in basal-like breast cancers and predicts poor outcome. *Am J Pathol* **176:** 2911-2920

Lugli A, Zlobec I, Minoo P, Baker K, Tornillo L, Terracciano L, Jass JR (2007) Prognostic significance of the wnt signalling pathway molecules APC, beta-catenin and E-cadherin in colorectal cancer: a tissue microarray-based analysis. *Histopathology* **50:** 453-464

Martensson A, Oberg A, Jung A, Cederquist K, Stenling R, Palmqvist R (2007) Beta-catenin expression in relation to genetic instability and prognosis in colorectal cancer. *Oncology reports* **17:** 447-452

Perou CM, Sorlie T, Eisen MB, van de Rijn M, Jeffrey SS, Rees CA, Pollack JR, Ross DT, Johnsen H, Akslen LA, Fluge O, Pergamenschikov A, Williams C, Zhu SX, Lonning PE, Borresen-Dale AL, Brown PO, Botstein D (2000) Molecular portraits of human breast tumours. *Nature* **406:** 747-752

Sorlie T, Tibshirani R, Parker J, Hastie T, Marron JS, Nobel A, Deng S, Johnsen H, Pesich R, Geisler S, Demeter J, Perou CM, Lonning PE, Brown PO, Borresen-Dale AL, Botstein D (2003) Repeated observation of breast tumor subtypes in independent gene expression data sets. *Proceedings of the National Academy of Sciences of the United States of America* **100:** 8418-8423

Weigelt B, Reis-Filho JS (2009) Histological and molecular types of breast cancer: is there a unifying taxonomy? *Nat Rev Clin Oncol* **6:** 718-730

Wong SC, Lo ES, Chan AK, Lee KC, Hsiao WL (2003) Nuclear beta catenin as a potential prognostic and diagnostic marker in patients with colorectal cancer from Hong Kong. *Mol Pathol* **56:** 347-352

© EMBO

2nd Editorial Decision 15 September 2012

Thank you for the submission of your revised manuscript "WNT10B/β-catenin Signaling Induces HMGA2 and Proliferation in Metastatic Triple-Negative Breast Cancer" to EMBO Molecular Medicine. We have now finally received the reports from the reviewers who were asked to re-review your manuscript.

As you will see, the Reviewers acknowledge that the manuscript was improved during revision. However, they still raise concerns about the IHC quality and the interpretation of some results. Since we do acknowledge the potential interest of your findings, we would be willing to consider a revised manuscript with the understanding that the referee concerns must be convincingly and conclusively addressed.

On a more editorial note, in your revised manuscript, please address the following points:

- For experiments involving human subjects the submission must include a statement that informed consent was obtained from all subjects and that the experiments conformed to the principles set out in the WMA Declaration of Helsinki [http://www.wma.net/en/30publications/10policies/b3/] and the NIH Belmont Report [http://ohsr.od.nih.gov/guidelines/belmont.html]. Please see our Guide to Authors for further information and provide the necessary information in the respective Material and Methods part.

- The description of all reported data that includes statistical testing must state the name of the statistical test used to generate error bars and P values, the number (n) of independent experiments underlying each data point (not replicate measures of one sample), and the actual P value for each test (not merely 'significant' or 'P < 0.05').

- In addition, we noted that the resolution of some figure panels (including the IHC panels) is rather low and that the contrast of some panels (for example Fig 4G) should be improved. Please include higher resolution pictures and note that immunoblots should be surrounded by a black line to indicate the borders of the blot, if the background is faint (for example Fig 3G).

- We noted that you included unpublished data in your point-by-point response. These would be published in a Peer Review Process File. Please let us know whether you agree with their publication, if you would like to delete the data from the file or if you would like to opt-out of having the PRPF published.

I look forward to seeing a revised version of your manuscript as soon as possible.

Yours sincerely,

Editor
EMBO Molecular Medicine

***** Reviewer's comments *****

Referee #1 (Remarks):

The authors have adequately addressed, clarified or provided reasonable explanations to almost all of my concerns. Overall, the quality of the paper has improved; the flow of the manuscript eased and now the reader can identify a working hypothesis that is largely supported by the data.

With regard to my major points, the authors have made an effort to provide strong clinical evidence to position their findings. Moreover, they have improved their statistical analysis particularly with regards to patient data analysis. The reader can now link the model of WNT10B with the expression of HMGA2, their mechanistic association. Most importantly, they have restricted their claims regardless the stem cell related matters posted in their previous manuscript version. These changes have largely reduced the noise within the text and streamline the message. Besides, most of the

weakly supported data has been eliminated.

Focusing on the mechanistic analysis the use of two independent Wnt-related inhibitors, the causal validation of HMGA2 downstream of WNT10b and others have strengthened the authors' claims. Finally, a proper re-evaluation of the MDA-231 data and the INK4 locus re-activation has been settled.

Thus, the new data and comments provided are convincing. Moreover, the explanation and the unpublished disclosed information regarding point 3 does suffice. Finally, they have improved and upgraded the quality of the figures.

Given all the above, the manuscript represents a substantial advance on the field pending on some remaining sticky points that must be addressed.

1. Page 6, first paragraph, line 2-3. "the translation wnt10b model may provide a novel therapeutic tool to develop inhbitiors for stem cell-like cancers..." This affirmation is pure speculation, not supported at all by the data. The authors must refer to TNBC at most.

2. Page 7, 4th line. "... on the TMA from Ohio were selected ..." . To define the origin of a TMA by stating "from Ohio" is not scientifically appropriate. Please refer to Ohio Tissue Bank Cohort or use acronym.

3. Page 7, 6th line. The sentence "In contrast, the most of the TNBC..." contains too many "the"

4. Page 7, last paragraph. It is not clear in which data set or patient cohort the statistics are calculated. Please clarify

5. Page 8, last sentence 2nd paragraph. The sentence is not supported by the data since the authors do not clarify the unique causality and specificity of WNT10B for TNBC tumors (there are other Wnt-ligands) and no experimental data on preclinical mouse models is provided (i.e. WNT10b KO crossed with PyMT or Wnt-inhibitors blockade of WNT10B driven tumors in vivo). Thus, the sentence is pure speculation and should be contained in the discussion section.

6. Page 21, 2nd paragraph, 3rd sentence. It is not clear which patients (data set, cohort etc) do the authors refer to. Please clarify.

7. Pag 21, last paragraph. "In summary, we have presented evidence supporting that HMGA2 expression is an important clinical marker ...". This sentence overstates the power of HMGA2. Clearly, the authors present clinical evidence of the capacity of HMGA2 to predict survival outcome in TNBC. Yet, this marker is not independent of other clinical variables currently used in the standard of care (Tumor size, nuclear grade or even Ki67). Thus, its importance is limited to the current research and the understanding of the TNBC biology. The authors must eliminate the word "important" prior to "clinical marker" since given the association with tumor size this marker currently does not have sufficient value to be implemented in the clinical setting, thus is not an important clinical marker.

8. Page 23, 1st paragraph, 2nd line. "We demonstrate that this is due to the presence of WNT10B-ligand...". This sentence is too strong since other Wnt-ligands cannot be excluded to contribute. Please re-phrase.

9. Page 23, 1st paragraph, last sentence. The authors must clarify that the marker is not independent of other well-established markers to avoid any over interpretation by the readers.

10. Page 24, 2nd paragraph. The authors enthusiastic interpretation of the potential application of the data should be tune down by clarifying that the need of testing this hypothesis in preclinical experimental models (i.e. WNT10b transgenic mice) is warranted.

Referee #2:

I am still not convinced. the antibody and staining specificity for wnt and beta-catenin as well as others is still a concern.

2nd Revision - authors' response 26 October 2012

(please see next page)

2nd Revision - authors' response 26 October 2012

Referee #1 (Remarks):

The authors have adequately addressed, clarified or provided reasonable explanations to almost all of my concerns. Overall, the quality of the paper has improved; the flow of the manuscript eased and now the reader can identify a working hypothesis that is largely supported by the data.

With regard to my major points, the authors have made an effort to provide strong clinical evidence to position their findings. Moreover, they have improved their statistical analysis particularly with regards to patient data analysis. The reader can now link the model of WNT10B with the expression of HMGA2, their mechanistic association. Most importantly, they have restricted their claims regardless the stem cell related matters posted in their previous manuscript version. These changes have largely reduced the noise within the text and streamline the message. Besides, most of the weakly supported data has been eliminated.

Focusing on the mechanistic analysis the use of two independent Wnt-related inhibitors, the causal validation of HMGA2 downstream of WNT10b and others have strengthened the authors' claims. Finally, a proper re-evaluation of the MDA-231 data and the INK4 locus re-activation has been settled.

Thus, the new data and comments provided are convincing. Moreover, the explanation and the unpublished disclosed information regarding point 3 does suffice. Finally, they have improved and upgraded the quality of the figures.

Given all the above, the manuscript represents a substantial advance on the field pending on some remaining sticky points that must be addressed.

1. Page 6, first paragraph, line 2-3. "the translation wnt10b model may provide a novel therapeutic tool to develop inhibitors for stem cell-like cancers..." This affirmation is pure speculation, not supported at all by the data. The authors must refer to TNBC at most.

   Point by point response: We have removed **"…**for stem cell-like cancers and…"

2. Page 7, 4th line. "... on the TMA from Ohio were selected ..." To define the origin of a TMA by stating "from Ohio" is not scientifically appropriate. Please refer to Ohio Tissue Bank Cohort or use acronym.

   Point by point response: We have corrected the following "from Ohio" to "...the Ohio Tissue Bank Cohort…"

3. Page 7, 6th line. The sentence "In contrast, the most of the TNBC..." contains too many "the"

   Point by point response: We removed one of "the" from the sentence.

4. Page 7, last paragraph. It is not clear in which data set or patient cohort the statistics are calculated. Please clarify.

   Point by point response: We clarified the above by adding our collection of 59 TNBC; it now states **"**To this end we utilized the available clinical data from our collection of 59 TNBC samples,…"

5. Page 8, last sentence 2nd paragraph. The sentence is not supported by the data since the authors do not clarify the unique causality and specificity of WNT10B for TNBC tumors (there are other Wnt-ligands) and no experimental data on preclinical mouse models is provided (i.e. WNT10b KO crossed with PyMT or Wnt-inhibitors blockade of WNT10B driven tumors in vivo). Thus, the sentence is pure speculation and should be contained in the discussion section.

Point by point response: We removed the sentence “Thus, we should consider WNT10B/β-catenin signaling as a potential therapeutic target for TNBC.”

6. Page 21, 2nd paragraph, 3rd sentence. It is not clear which patients (data set, cohort etc) do the authors refer to. Please clarify.

Point by point response: We corrected the sentence and added “basal-like” and “(Fig. 1E)” for clarification; it now reads as follows: “Remarkably, basal-like patients with high *HMGA2* mRNA levels (80% of the patients) had a significant lower probability of survival than those with low *HMGA2* levels [log-rank test P=0.0308, hazard ratio (HR) =2.0], albeit fewer patients passed the criteria than in the WNT10B analysis (Fig. 1E).”

7. Page 21, last paragraph. "In summary, we have presented evidence supporting that HMGA2 expression is an important clinical marker ...". This sentence overstates the power of HMGA2. Clearly, the authors present clinical evidence of the capacity of HMGA2 to predict survival outcome in TNBC. Yet, this marker is not independent of other clinical variables currently used in the standard of care (Tumor size, nuclear grade or even Ki67). Thus, its importance is limited to the current research and the understanding of the TNBC biology. The authors must eliminate the word "important" prior to "clinical marker" since given the association with tumor size this marker currently does not have sufficient value to be implemented in the clinical setting, thus is not an important clinical marker.

Point by point response: We corrected the sentence and eliminated the word "important" prior to "clinical marker"; it now reads as follows: “In summary, we have presented evidence supporting that HMGA2 expression is a clinical marker that has the capacity to predict survival outcome in TNBC and basal-like breast cancer patients.”

8. Page 23, 1st paragraph, 2nd line. "We demonstrate that this is due to the presence of WNT10B-ligand...". This sentence is too strong since other Wnt-ligands cannot be excluded to contribute. Please re-phrase.

Point by point response: We removed the strong sentence "We demonstrate that this is due to the presence of WNT10B-ligand..." and replaced it with the following: “Additionally, we correlate the expression of the WNT10B-ligand in similar regions but we cannot at this time exclude that other WNT-ligands mediate canonical Wnt-signaling (i.e. WNT1 or WNT3A amongst others) that may be responsible for the above observation.”

9. Page 23, 1st paragraph, last sentence. The authors must clarify that the marker is not independent of other well-established markers to avoid any over interpretation by the readers.

Point by point response: We removed the above statement.

10. Page 24, 2nd paragraph. The author's enthusiastic interpretation of the potential application of the data should be tune down by clarifying that the need of testing this hypothesis in preclinical experimental models (i.e. WNT10b transgenic mice) is warranted.

Point by point response: We have addressed the reviewer's concerns; the 2nd paragraph now reads as follows:

"Mechanistically, our data now provide evidence that WNT10B activates canonical β-catenin signaling leading to proliferation and up-regulation of HMGA2 in TNBC directly by components of the canonical Wnt signaling pathway. Furthermore, the Wnt/β-catenin inhibitor ICG-001 can significantly suppress mRNA and protein levels of HMGA2 and block proliferation in TNBC cells (model in Fig. 6E). Our data also illustrate that HMGA2 expression is significantly correlated with the capacity to predict metastasis in our relatively-small TNBC cohort samples (i.e. 12/45) (Fig. 6D and Supplemental Fig. S7D). Accordingly, we observed that *Wnt10b^{LacZ}* transgenic mice give rise to rare spontaneous lung metastasis expressing high levels of *Hmga2* and HMGA2 in FACS-sorted Lin^{-}LacZ^{+} lung metastasis cells (Wend *et al.*, Wnt 30-Years EMBO symposium, 2012 Netherlands). The above results support the rationale that the *Wnt10b/β-catenin/Hmga2* axis needs to be further tested for their functional role in TNBC and related lung-metastasis in a preclinical experimental model.

Referee #2:

I am still not convinced. the antibody and staining specificity for wnt and beta-catenin as well as others is still a concern.

COMMENTS: We thank the reviewer for raising the concerns in regards to the specificity of the antibodies used for our current manuscript. I would like to note at this time that we are taking the concerns raised by the reviewer very seriously and have made a tremendous effort to address as many of the concerns as possible (within the limits of the available antibodies used in the field). Furthermore, to alleviate the reviewer concerns we have sequentially diluted all of the antibodies from 1/25 to as much as 1/5000 optimizing each antibodies specificity while concurrently changing ranges of pH's and optimizing buffers. We have now included as many controls to alleviate the concerns for the IHC of human samples including commercially available Tissue Microarrays (TMA's) with a wide range of breast cancer subtypes and "adjacent-normal breast tissue" from nearby tumors. We have conducted IHC experiments on various stages in embryonic development contrasting wildtype versus Wnt10b-knockout mice utilizing our antibodies. For the purpose of this manuscript we have focused on the development of mouse mammary gland placodes at embryonic day E14.5. We now have utilized UCLA's imaging core that has state of the art advanced light-microscopes in collaboration with core director Dr. Laurent Bentolila to improve the quality of our images for this MS. Finally, I would like to thank the reviewer for the due diligence to expect specificity for IHC. It has made our work more fundamentally solid and hopefully you will appreciate our latest attempt to alleviate the concerns raised.

Point by point response: We have addressed the reviewer's concerns with the following changes:

1) Figure 1A. TMA's have been reimaged for improvement of the resolution and improving Wnt10b IHC contrast.

2) Figure 1C was reimaged with advanced light-microscopes for higher magnification illustrating two improved inserts that highlights better contrast of WNT10B cytoplasmic localization vs. no nuclear localization.

3) We provide a new Supplemental Figure 1 A-B to alleviate the concerns for the specificity of the WNT10B antibody.

i) We conducted immunoblotting against WNT10B protein expression highlighting the strong-specificity of the antibody (ab91201 5A7, Abcam) in transgenic primary tumors and a secondary syngeneically transplanted tumor vs. that of the established cell line utilized throughout our manuscript (WZA^{LacZ}). We observed no upper mobility cross-reacting bands but a very specific single band corresponding with the correct molecular weight predictive for WNT10B protein (43 kDa). The reviewer raised concerns from the previous (i.e. 1st) critique that upper mobility bands ranging from 75Kd-130Kd analyzed by western (described by the manufacture) to be a problem.

ii) We utilized our mouse normal mammary gland cell line (NMuMG) overexpression system to highlight Wnt10b-specificty. The parental NMuMG cell line is known to produce all the Wnt-ligands (but known to express lower levels of *Wnt10b*). The NMuMG-Wnt10b overexpressing cell line was engineered to only express ~2-fold more Wnt10b mRNA levels and was subsequently re-established in my lab to ensure specificity of Wnt10b expression levels (Miranda-Carboni et al, 2008). The results illustrate that we can identify the correct specific-band for Wnt10b (43 kDa) as well as the upper mobility 75 kDa - 130 kDa bands that are proportionally increased in the overexpressing Wnt10b cell line. This may suggest that it may be due to post-translational modifications of the Wnt10b protein (ubiquitination, myristoylation, palmitoylation or glycosylation) as this is well known for Wnt ligands to be necessary for activating their signaling, albeit we did not address this experimentally.

iii) We also illustrate by western blot analysis a band that corresponds to the correct specific-band for WNT10B in two human triple-negative breast cancer cell lines.

iv) To further alleviate concerns, we tested for the cross-reactivity of the Wnt10b antibody by IHC in wildtype vs. Wnt10b-knockout ($Wnt10b^{-/-}$) embryos at E14.5. We illustrate specific cytoplasmic-localization of WNT10B in wildtype mice in the epidermis layer, mesenchymal tissue and mammary gland placodes. Importantly, positive staining of these tissue structures is lost in $Wnt10b^{-/-}$ embryos. To further discuss the specificity, other Wnt-ligands (Wnt6 and Wnt10a) are expressed in the mammary gland placodes and several other Wnt–ligands are expressed in the epidermis layer at E14.5 (Wnt3, 4, 6, 7b, 10a, 10b and alternatively Wnt7a and 16) reviewed extensively (Wend et al, 2010). Our IHC data strongly suggest that other Wnt-ligands are not cross-reacting with this particular Wnt10b antibody because we see no IHC background staining in $Wnt10b^{-/-}$ mice. The dilution used for this experiment was at 1/1000 and DAB staining was developed with DAKO kits for 5 minutes.

4) We provide a new Supplemental Figure 8 to alleviate the concerns for the specificity of the β-catenin antibody staining.

COMMENTS: We are concise of the controversy in breast cancer for β-catenin in regards to IHC data and its relevance, due to the lack of mutations in the Wnt/beta-catenin pathway. Especially, when it is contrasted to colorectal cancer where such mutations have been well established and proven to be fundamental for the disease. Nonetheless, several rationales have been provided for the relevance of Wnt/β-catenin signaling in breast cancer that is supported by the literature. Further rationale to investigate the Wnt/β-catenin pathway in TNBC was provided by a study that analyzed publically available data sets by gene expression profiling and robustly identified 548 TN breast tumor data-sets (Lehmann et al, 2011). The results illustrated that TN tumors are highly heterogeneous comprising at least seven subtypes. Distinct gene ontologies were utilized to identify important pathways. Among these Wnt/β-catenin signaling was identified to be significantly activated in mesenchymal-like (ML), mesenchymal stem-like (MSL), basal-like 2 (BL2) and to less-of-a-degree in the basal-like 1 (BL1) TN subtype. MDA-MB-231 cells are MSL and MDA-MB-468 is a BL1 breast cancer cell line (Lehmann et al, 2011). Furthermore, published data for colorectal cancer and other studies on TNBC have shown that the overall content of nuclear and increased cytoplasmic β-catenin varies between 5-80% in the tumor tissues (Geyer et al, 2011; Khramtsov et al, 2010; Lugli et al, 2007; Martensson et al, 2007; Wong et al, 2003). Moreover, the distribution of nuclear β-catenin throughout the tumor tissue is very heterogeneous and often found at the invasive tumor front (Brabletz et al, 2005; Fodde & Brabletz, 2007). Additionally, a particular publication illustrated the relevance of IHC with an antibody for activated β-catenin and was able to predict patient outcome in breast cancer (Lin et al, 2000).

Point by point response: We have addressed the reviewer concerns by providing a new set of IHC results that we have optimized (by sequentially changing the pH and diluting the antibodies) to enhance detection of nuclear localization and to contrast it to a monoclonal non-phospho (ACTIVE) β-catenin (Ser33/37/Thr41) antibody recognizing only active β-catenin, which is not phosphorylated (Cell Signaling, #8814). In our new Supplemental Figure 8 we compare and contrast TMAs from different breast cancer subtypes (ER^+, PR^+, $HER2^+$, and Triple-Positive [TP]) to TNBC using two β-catenin antibodies (Sigma C2206 and Cell Signaling #8814) . In addition, we verify activation of Wnt/β-catenin signaling in TNBC by IHC for AXIN2 (Abcam, ab32197), which is a well-known target of the Wnt/β-catenin pathway.

i) Supplemental Figure 8A illustrates our optimization with (what we will call) old pan-β-catenin antibody (Sigma C2206) to ER^+, PR^+, HER^+ and TP^+. A colorectal cancer (CRC) sample served as a positive control to contrast it directly to TN tumors. The data reveals that ER^+ tumors have low levels of membrane-bound β-catenin in contrast to PR^+ tumors that have clear cell adhesion localization of β-catenin but little if any cytoplasmic accumulated β-catenin localization. $Her2^+$ tumors have both sublocalization of β-catenin (membrane-bound and cytoplasmic) and TP are mostly negative. We contrast this with the TN tumors and the antibody is highly detectable and highly accumulated in the cytoplasm (yellow arrow blue nuclei) with a few rare cells that are nuclear localized (red arrows). CRC is our control for cytoplasmic accumulation and nuclear localization of β-catenin.

ii) Supplemental Figure 8B shows CRC as a control to contrast it directly to TN with ACTIVE non-phosphorylated β-catenin (Cell Signaling #8814). For the TNBC samples we illustrate reduced stroma staining and greater nuclear and cytoplasmic localization (yellow arrows) and in one sample mostly nuclear localization (red arrows). When the

activated non-phosphorylated β-catenin antibody is tested against other breast cancer subtypes (ER^+,PR^+ and TP^+) it is substantially decreased and consistent with our modeling. In $HER2^+$ breast cancer one can find mostly cytoplasmic non-phospho β-catenin but much less compared to the pan-antibody and much less than in the TN tumors. This is again consistent with our modeling in published literature. It is known that a subclass of TN carries a $Her2^+$-Wnt signature and *vice versa* thus these results would be consistent with some expression level of AXIN2 (Perou, 2010).

iii) In Supplemental Figure 8C we reanalyzed and optimized the IHC for AXIN2 which is known to be a direct Wnt/β-catenin gene target representing transcriptionally active β-catenin. The results illustrate that other subtypes of breast cancers have little or no expression of AXIN2 with the exception of HER2 tumors. Nonetheless, when contrasted to either CRC or TN the levels of AXIN2 protein expression are high and localized to the cytoplasm for the most part.

We have further addressed the reviewer concerns about antibody staining and specificity by providing better pictures of our IHC results for the detection of ERα, PR, HER2, and HMGA2. The results strengthen our claim that our *Wnt10b*^{LacZ} tumor mouse model gives raise to triple-negative mammary tumors highly expressing HMGA2.

iv) Figure 2A; was reimaged utilizing the advanced light-microscope highlighting the specific staining for ERα, PR and HER2 at higher resolutions (inserts 60X). The colors have been greatly improved to better reflect contrast in the nuclear stained cells *vs.* negative stained cells then our previous images.

v) Figure 2D (IHC for HMGA2); we reimaged with the advanced light-microscope and changed the contrast to better reflect specific-localization. Dilutions were done at 1/2500 with 2 minutes development using DAKO kits. We have array data confirming that HMGA2 is the highest regulated gene in our model. With the new images we can see greater specific labeling of HMGA2 as in some cells the blue nuclei is very evident (black arrowheads). Furthermore, HMGA2 is highly expressed in the cytoplasm with a degree of the cells having nuclear only staining.

Brabletz T, Hlubek F, Spaderna S, Schmalhofer O, Hiendlmeyer E, Jung A, Kirchner T (2005) Invasion and metastasis in colorectal cancer: epithelial-mesenchymal transition, mesenchymal-epithelial transition, stem cells and beta-catenin. *Cells Tissues Organs* **179:** 56-65

Fodde R, Brabletz T (2007) Wnt/beta-catenin signaling in cancer stemness and malignant behavior. *Curr Opin Cell Biol* **19:** 150-158

Geyer FC, Lacroix-Triki M, Savage K, Arnedos M, Lambros MB, MacKay A, Natrajan R, Reis-Filho JS (2011) beta-Catenin pathway activation in breast cancer is associated with triple-negative phenotype but not with CTNNB1 mutation. *Mod Pathol* **24:** 209-231

Khramtsov AI, Khramtsova GF, Tretiakova M, Huo D, Olopade OI, Goss KH (2010) Wnt/beta-catenin pathway activation is enriched in basal-like breast cancers and predicts poor outcome. *Am J Pathol* **176:** 2911-2920

Lehmann BD, Bauer JA, Chen X, Sanders ME, Chakravarthy AB, Shyr Y, Pietenpol JA (2011) Identification of human triple-negative breast cancer subtypes and preclinical models for selection of targeted therapies. *J Clin Invest*

Lin SY, Xia W, Wang JC, Kwong KY, Spohn B, Wen Y, Pestell RG, Hung MC (2000) Beta-catenin, a novel prognostic marker for breast cancer: its roles in cyclin D1 expression and cancer progression. *Proceedings of the National Academy of Sciences of the United States of America* **97:** 4262-4266

Lugli A, Zlobec I, Minoo P, Baker K, Tornillo L, Terracciano L, Jass JR (2007) Prognostic significance of the wnt signalling pathway molecules APC, beta-catenin and E-cadherin in colorectal cancer: a tissue microarray-based analysis. *Histopathology* **50:** 453-464

Martensson A, Oberg A, Jung A, Cederquist K, Stenling R, Palmqvist R (2007) Beta-catenin expression in relation to genetic instability and prognosis in colorectal cancer. *Oncology reports* **17:** 447-452

Miranda-Carboni GA, Krum SA, Yee K, Nava M, Deng QE, Pervin S, Collado-Hidalgo A, Galic Z, Zack JA, Nakayama K, Nakayama KI, Lane TF (2008) A functional link between Wnt signaling and SKP2-independent p27 turnover in mammary tumors. *Genes & development* **22:** 3121-3134

Perou CM (2010) Molecular stratification of triple-negative breast cancers. *Oncologist* **15 Suppl 5:** 39-48

Wend P, Holland JD, Ziebold U, Birchmeier W (2010) Wnt signaling in stem and cancer stem cells. *Semin Cell Dev Biol* **21:** 855-863

Wong SC, Lo ES, Chan AK, Lee KC, Hsiao WL (2003) Nuclear beta catenin as a potential prognostic and diagnostic marker in patients with colorectal cancer from Hong Kong. *Mol Pathol* **56:** 347-352

3rd Editorial Decision 07 November 2012

Thank you for the submission of your revised manuscript to EMBO Molecular Medicine. I am pleased to inform you that we will be able to accept your manuscript pending the following final amendments:

The description of all reported data that includes statistical testing must state the name of the statistical test used to generate error bars and P values, the number (n) of independent experiments underlying each data point (not replicate measures of one sample), and the actual P value for each test (not merely 'significant' or 'P < 0.05').

Also, please follow the instructions for submission of the revised form as described below where you have not done so before.

Please submit your revised manuscript within two weeks. I look forward to seeing a revised form of your manuscript as soon as possible.

Yours sincerely,

Editor
EMBO Molecular Medicine
