# Supplementary material for: WNT10B/β-catenin signalling induces HMGA2 and proliferation in metastatic triple-negative breast cancer
Source: EMBO Mol Med. 2013 Jan 11;5(2):264–79. doi: 10.1002/emmm.201201320 (PMC3569642; doi:10.1002/emmm.201201320)
Supplement: Supplementary file 2 [file emmm0005-0264-SD2.pdf]

Supplemental Section:

1) Eight supplemental figures and legends

2) Supplemental Materials and Methods

3) Table_S1: Clinical Characteristics of Breast Cancer Patients

4) Table_S2: Oligonucleotide sequences for transient RNAi

5) Table_S3: Primer sequences used for qt-PCR of ChIP samples

Figure S1. Verification of WNT10B-antibody specificity by WNT10B expression analysis in mammary tumor samples, cell lines and embryonic mammary anlagen. (A) Western blot analysis verifying expression of WNT10B on the following was conducted: *Wnt10b^{LacZ}* tumor samples (1°: primary tumor, 2°: transplanted secondary tumor), a *Wnt10b^{LacZ}* tumor-derived cell line (WZA^{LacZ}), parental NMG vs. NMG-10b-overexpressing cells, and human TNBC cell lines MDA-MB-231 and MDA-MB-468. Actin served as a loading control. Molecular weights are indicated in kDa on the left side. Note that the predicted molecular weight of WNT10B is 43 kDa. (**B)** IHC of WNT10B in wildtype and *Wnt10b*-knockout (*Wnt10b$^{-/-}$*) mammary gland anlagen (MA) at embryonic day 14.5. Dashed black lines indicate MA. Note that the WNT10B antibody does not stain the epidermal layer (EL) and MA of *Wnt10b$^{-/-}$* mice. The WNT10B antibody shows no cross-reactivity with other Wnt ligands that are co-expressed in these regions at the same time (e.g., Wnt 3, 4, 5, 6, 7a, 7b, 10a, 11, 16) (Reddy *et al.* 2001). Bar = 50 μm.

Figure S2. *Wnt10b*-driven tumors are consistent with a triple-negative (ER$^-$PR$^-$HER2$^-$) and basal-epithelial expression phenotype and *Wnt10b^{LacZ}*-induced tumors show active Wnt/β-catenin signaling. (A) Hierarchical cluster analysis of 10-week old virgin mammary gland, 12-day pregnant mammary tissue, MMTV-*Wnt10b^{TG}*, and MMTV-*ErbB2^{TG}* mouse tumors. Triplicate samples were processed for microarray analysis and data was analyzed by GeneSpring™. **(B)** QT-PCR validates array data for *ErbB2*, *PR* and *ERα*. Experiments were normalized to *GADPH* (n=3). Values represent fold induction of over wild type 10 week old virgin mammary gland values. Values represent the means ± 2 standard deviations. **(C)** Verification of CK5 and CK6 expression levels by IHC in *Wnt10b*^{TG}-driven tumors. CK5$^+$ and CK6$^+$ cells are not detectable in *ErbB2*^{TG} tumors. Insets show 40X magnifications. **(D)** Whole-mount β-galactosidase staining of wildtype (FVB, left) and *Wnt10b*^{LacZ} transgenic mammary glands (line WZ6 [low] and line WZA [high], and a mammary tumor [WZA, right]). 7X magnifications are shown. **(E)** Analysis of β-galactosidase (LacZ) activity in tissues from virgin 10 month aged-match FVB wildtype mammary gland #3, transgenic *MMTV-Wnt10b*^{LacZ} founder WZA88F mammary gland #3 (low expression sister) and founder WZA89F mammary gland #3 tumor (high expression sister). **(F)** Adjacent sections from *Wnt10b*^{LacZ}-induced mammary tumors were analyzed by IF for the expression of β–Galactosidase/LacZ and by IHC for the expression of β-catenin and the Wnt/β-catenin target gene Axin2.

Figure S3. Wnt/β-catenin signaling is essential for the growth and the expression of a specific set of cell cycle regulators in triple-negative breast cancer cells. (A) Hierarchical cluster analysis of microarray data from 10-week old virgin mammary tissue, 12-day pregnant mammary tissue, and primary

mammary gland tumors from *MMTV-Wnt10b*^{TG} and *MMTV-ErbB2*^{TG} mice, respectively. Mean expression levels of 11 signature genes involved in proliferation ("intrinsic gene signature") and cell cycle regulation are shown that distinguish *MMTV-Wnt10b*^{TG} mammary tumors from *MMTV-ErbB2*^{TG} tumors and wildtype mammary tissue (each group n=3, data analyzed with GeneSpring^{TM}). **(B)** Verification of microarray gene expression data by qt-PCR for virgin and pregnant mammary tissue and for mammary tumors from *MMTV-Wnt10b*^{TG} and *MMTV-ErbB2*^{TG} mice. Error bars represent the means and the standard deviations from three independent experiments. **(C)** shRNA-mediated knockdown of *Hmga2* leads to attenuated growth of NMG-10b cells. Shown is one *shHmga2* clone compared to control NMG-10b and parental NMG cells. Error bars represent the means and the standard deviations from three independent experiments. **(D)** Reduced expression of *Hmga2* in two different *WZA*^{LacZ} clones after shRNA-mediated knockdown of *Hmga*2, as determined by qt-PCR. Error bars represent the means and the standard deviations from three independent experiments; p-value: ***=0.0005 versus *shGFP* control cells (Student's t-test). **(E)** Proliferation of *WZA*^{LacZ} mammary tumor cells upon treatment with the Wnt/β-catenin inhibitor ICG-001 (10 µM). Error bars represent the means and the standard deviations from three independent experiments. **(F)** Reduced expression of *Ccna2* in two different *WZA*^{LacZ} clones after shRNA-mediated knockdown of *Hmga*2, as determined by qt-PCR. Error bars represent the means and the standard deviations from three independent experiments; p-value: ***=0.0003 versus *shGFP* control cells (Student's t-test). P values of <0.05 were considered to be statistically significant (D, F).

Figure S4. *Wnt10b*-overexpressing MCF7 cells show increased expression of HMGA2 and Wnt target genes and still respond to estrogen treatment. BTL10 cells show active Wnt/β-catenin signaling, and unchanged proliferation of HUMEC cells upon treatment with ICG-001. (A) Analysis of mRNA expression levels of estrogen signaling targets (*XBP1* and *pS2*), *HMGA2* and Wnt target genes upon 17β-estradiol (E2) treatment, as analyzed by qt-PCR. Error bars represent the means and the standard deviations from three independent experiments. **(B)** Proliferation of MCF7 and MCF7-Wnt10b cells upon treatment with the Wnt/β-catenin inhibitor ICG-001 (10 µM in 1% DMSO). Error bars represent the means and the standard deviations from three independent experiments. **(C)** Analysis of *Hmga2* mRNA expression levels in MCF7-Wnt10b and MCF7 cells upon ICG-001 treatment, as analyzed by qt-PCR. Error bars represent the means and the standard deviations from three independent experiments. **(D)** Human triple negative BTL10 breast cancer cells show active Wnt/β-catenin signaling, as visualized using a lentiviral-transduced TCF/LEF^{GFP} reporter system. Percentage indicates GFP-positive BTL10 cells. **(E)** Proliferation of non-tumorigenic human HUMEC mammary cells upon

treatment with the Wnt/β-catenin inhibitor ICG-001 (10 μM in 1% DMSO). Error bars represent the means and the standard deviations from three independent experiments.

Figure S5. Wnt/β-catenin signaling is essential for the expression of a specific set of cell cycle regulators in triple-negative breast cancer cells. (A, B) Expression of genes regulating cell cycle and self-renewal in MDA-MB 231 and MDA-MB 468 cells after ICG-001 treatment (10 μM). Error bars represent the means and the standard deviations from three independent experiments. **(C)** Effect of siRNA-mediated knockdown of β-catenin on expression of genes involved in Wnt/β-catenin signaling and cell cycle regulation in MDA-MB231 cells, as analyzed by qt-PCR. Cells were transfected with 30 nM siRNA, mRNAs were analyzed after 48 hr. Error bars represent the means and the standard deviations from three independent experiments; p-values: a=0.01, b=0.04, c=0.03, d=0.01, e=0.02, f=0.02, g=0.02, h=0.04, i=0.03, j=0.02 versus si-Luciferase control cells (Student's t-test). **(D)** Expression of genes regulating cell cycle and self-renewal in BTL-10 cells upon control (1% DMSO) or ICG-001 (10 μM) treatment. Error bars represent the means and the standard deviations from three independent experiments; p-values: a=0.03, b=0.03, c=0.04, d=0.02, e=0.01, f=0.04 versus control treatment (Student's t-test). P values of <0.05 were considered to be statistically significant (C, D).

Figure S6. HMGA2 is highly and specifically expressed in triple-negative ($ER^-PR^-ErbB2^-$) human breast cancer. (A-C) IHC shows expression of nuclear HMGA2 in human TNBC samples from different patient groups. Hematoxylin-Eosin (HE) staining from an adjacent section is shown on the left. Bar, 50 μm. **(D)** Absence of HMGA2 expression in adjacent "normal" human breast tissue from four independent patient samples diagnosed with TNBC (i-iv). **(E)** HMGA2 is predominantly expressed in the nucleus of human TNBC. The graph demonstrates the relative frequency of nuclear HMGA2 immuno-reactivity in TNBCs (n=59).

Figure S7. TNBC patients expressing high levels of WNT10B or HMGA2 have an unfavorable clinical outcome. (A-D) Associations between WNT10B and HMGA2 expression (determined by IHC in TNBC patients) and clinical parameters such as tumor size (>1.5 cm), proliferation (Ki67%), nuclear grade and metastasis were measured on a continuous (A, B) or ordinal scale (C, D) and were evaluated using Kendall's Tau (A-C) or Fisher's exact test (D). Kendall's Tau (τ), p-value (*p*) and sample size (n) are depicted separately for each test. Additional clinical information on patient samples is provided in Supplemental Table 1. P values of <0.05 were considered to be statistically significant (A-D).

Figure S8. Human triple-negative breast cancers specifically express high levels of cytoplasmic and nuclear β-catenin as well as AXIN 2 indicating active Wnt/β-catenin signaling. (A, B) The subcellular localization of β-catenin in TNBC and other subtypes of human breast cancer (ER^+, PR^+, HER^+, and triple-positive [TP^+]) was analyzed by IHC using antibodies detecting all β-catenin protein (A) or exclusively activated β-catenin (B). A colorectal carcinoma (CRC) is shown as a positive control for β-catenin accumulation in the cytosol and nuclei. Arrows highlight tumor cells with cytoplasmic (yellow) and nuclear β-catenin (red). In ER^+, PR^+, HER^+, and TP^+ tumors β-catenin is observed predominantly at the membrane (A) or is only hardly detectable (B). Bar, 50 µm. **(C)** IHC analysis of AXIN 2 (a Wnt/β-catenin target gene) in TNBC and other subtypes of human breast cancer (ER^+, PR^+, HER^+, and TP^+), as analyzed by IHC. A colorectal carcinoma (CRC) is shown as a positive control for AXIN2 expression. In ER^+, PR^+, HER^+, and TP^+ tumors AXIN2 expression is very low or undetectable. Bar, 50 µm.

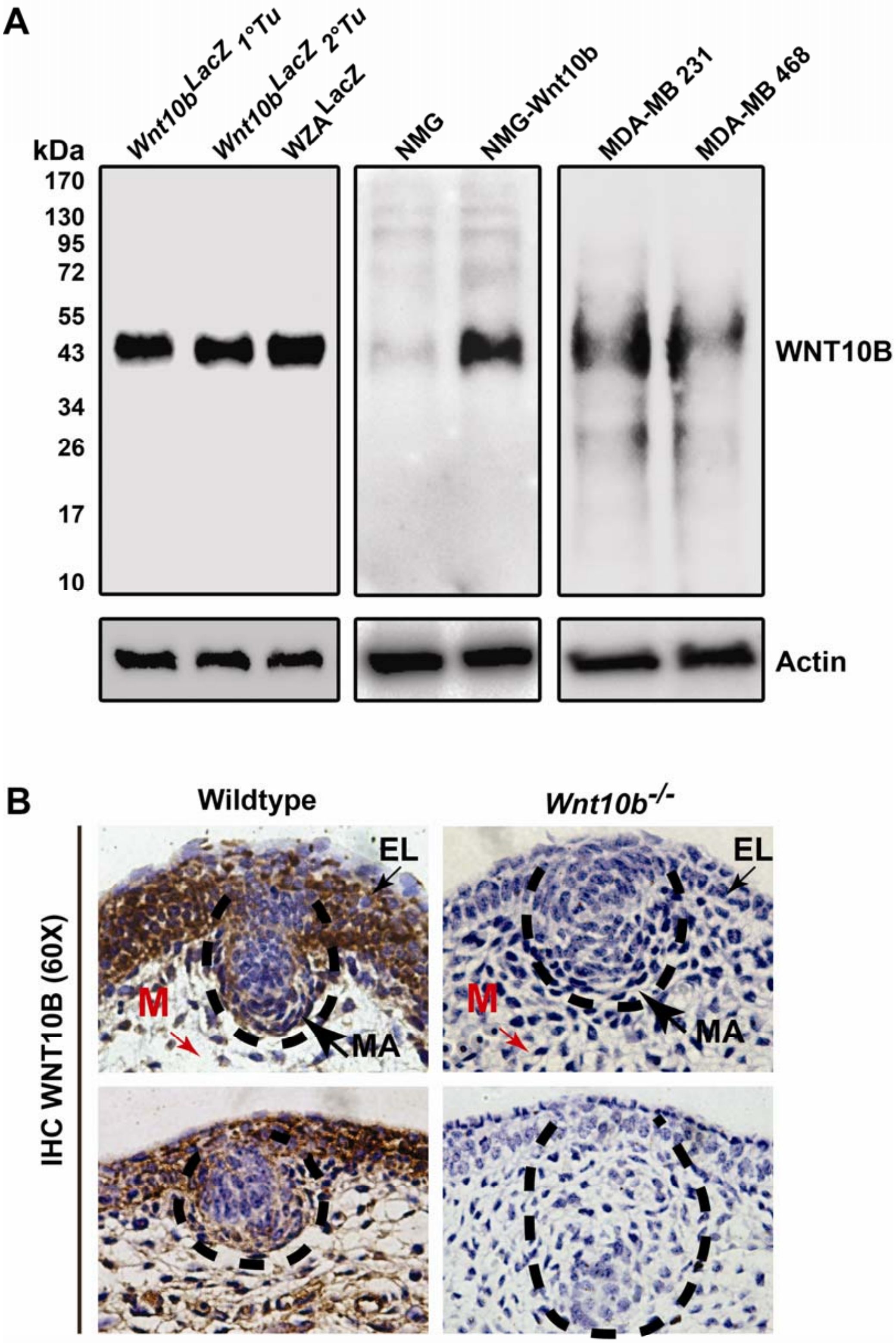

Suppl. Figure 1
Wend *et al.*

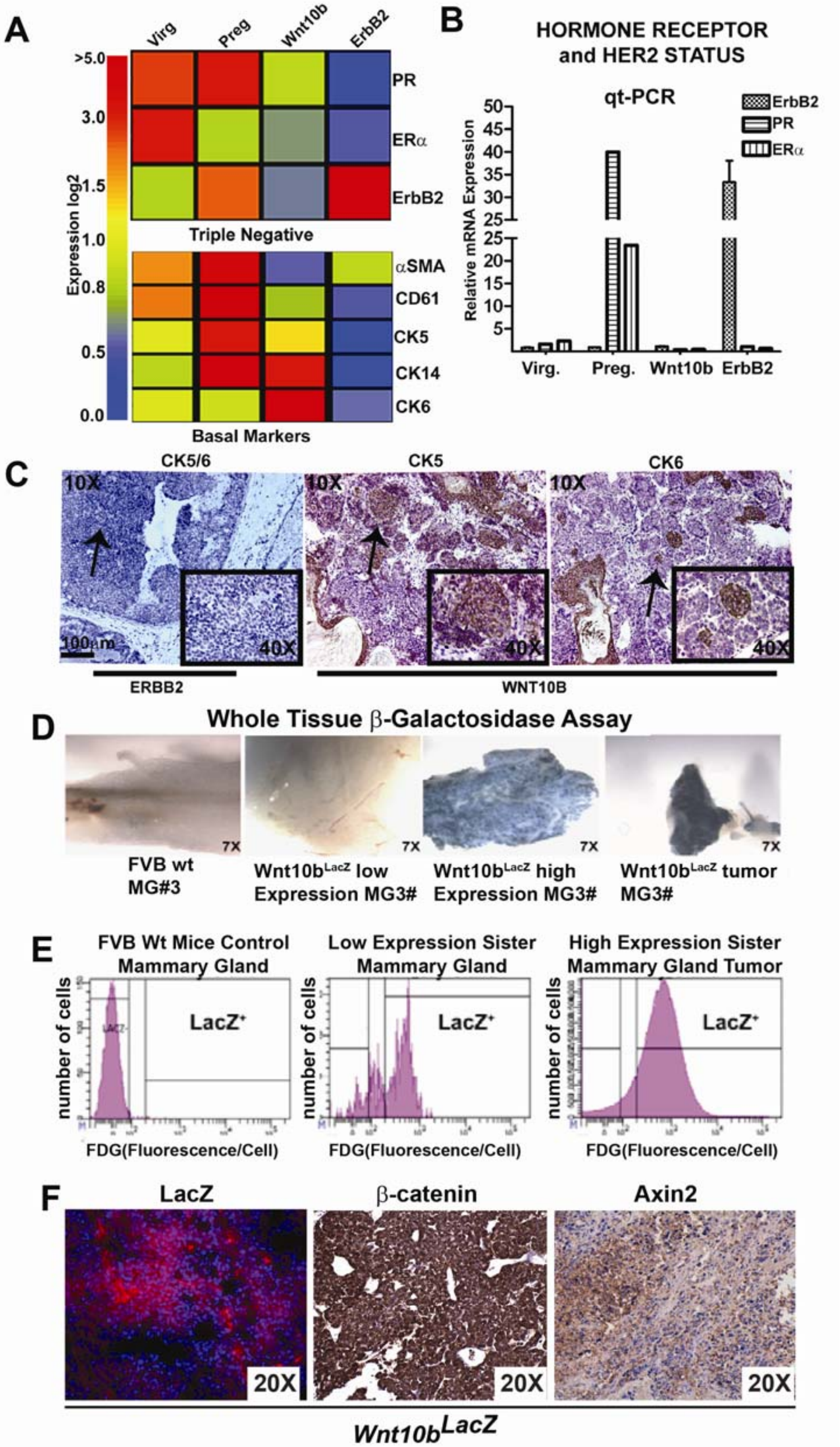

Suppl. Figure 2.
Wend *et al.*

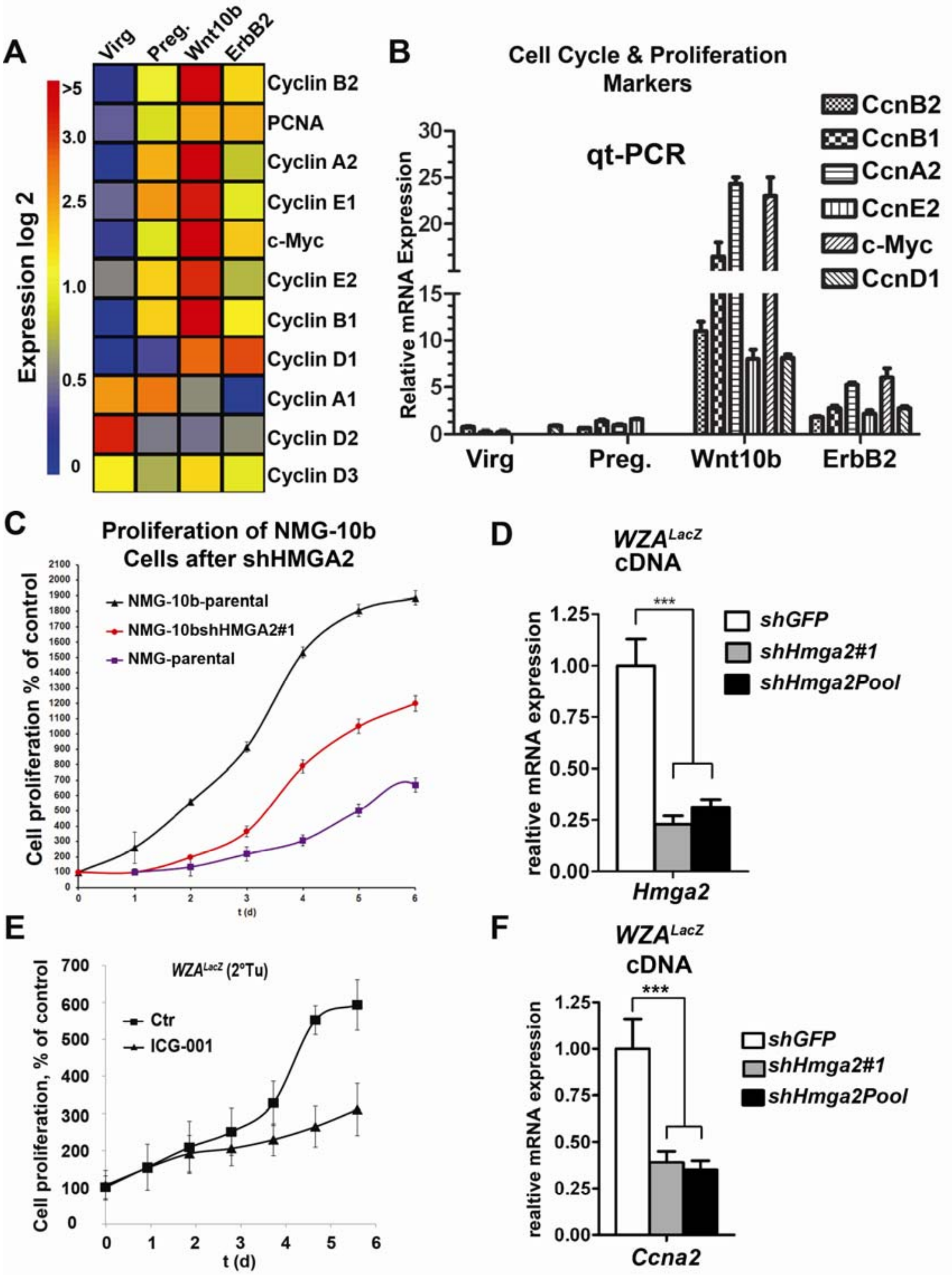

Suppl. Figure 3
Wend *et al.*

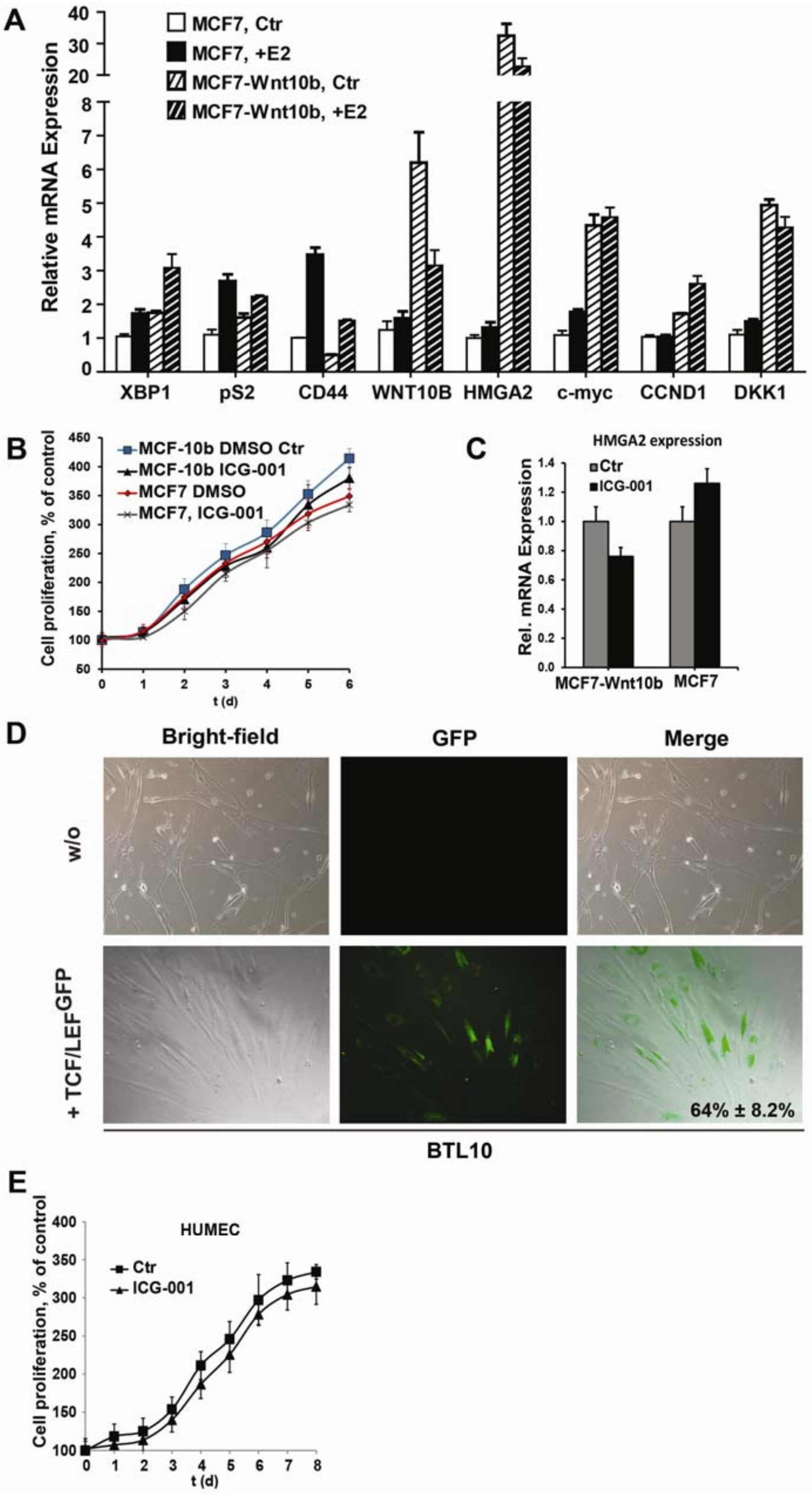

Suppl. Figure 4
Wend *et al.*

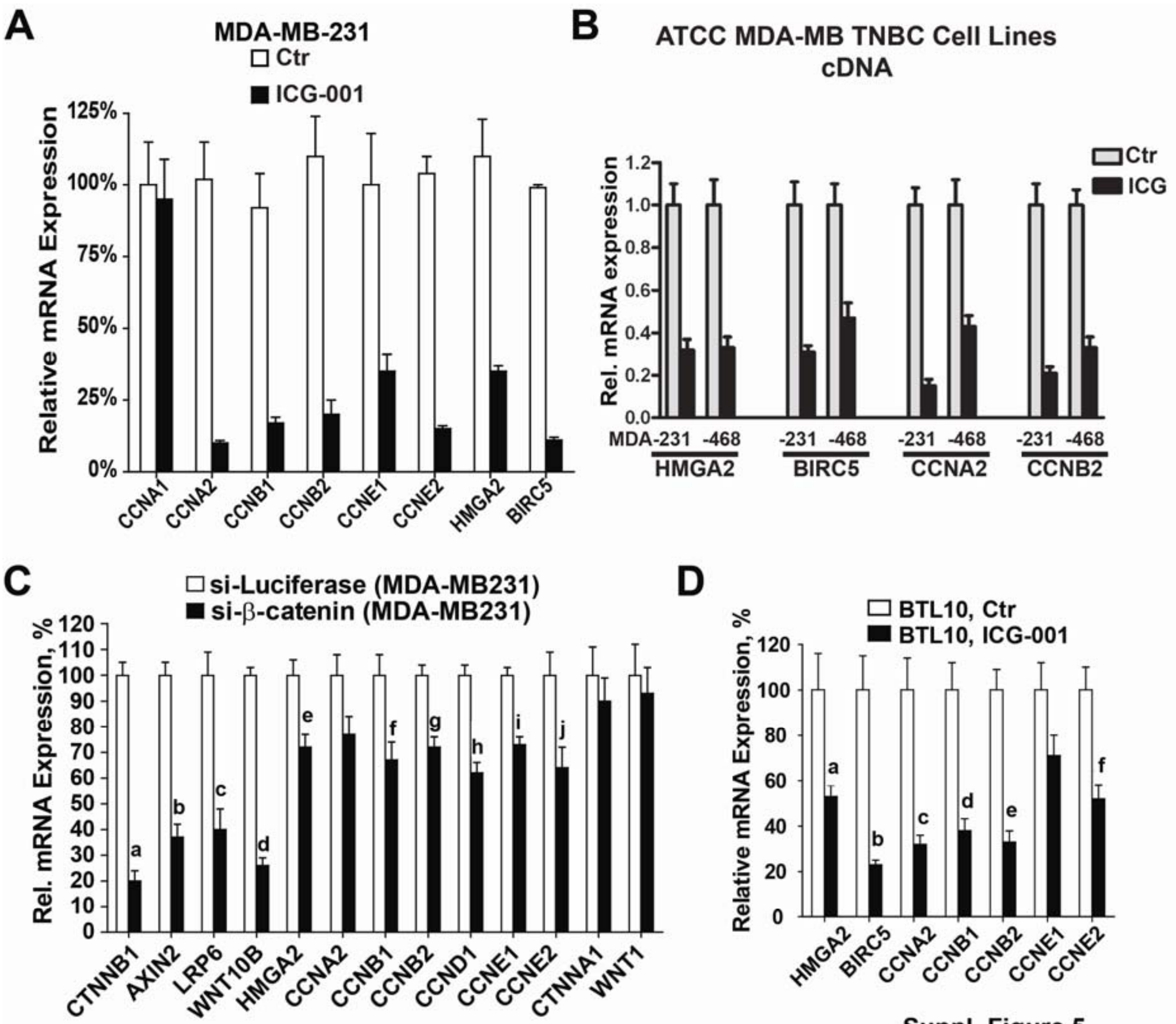

Suppl. Figure 5.
Wend *et al.*

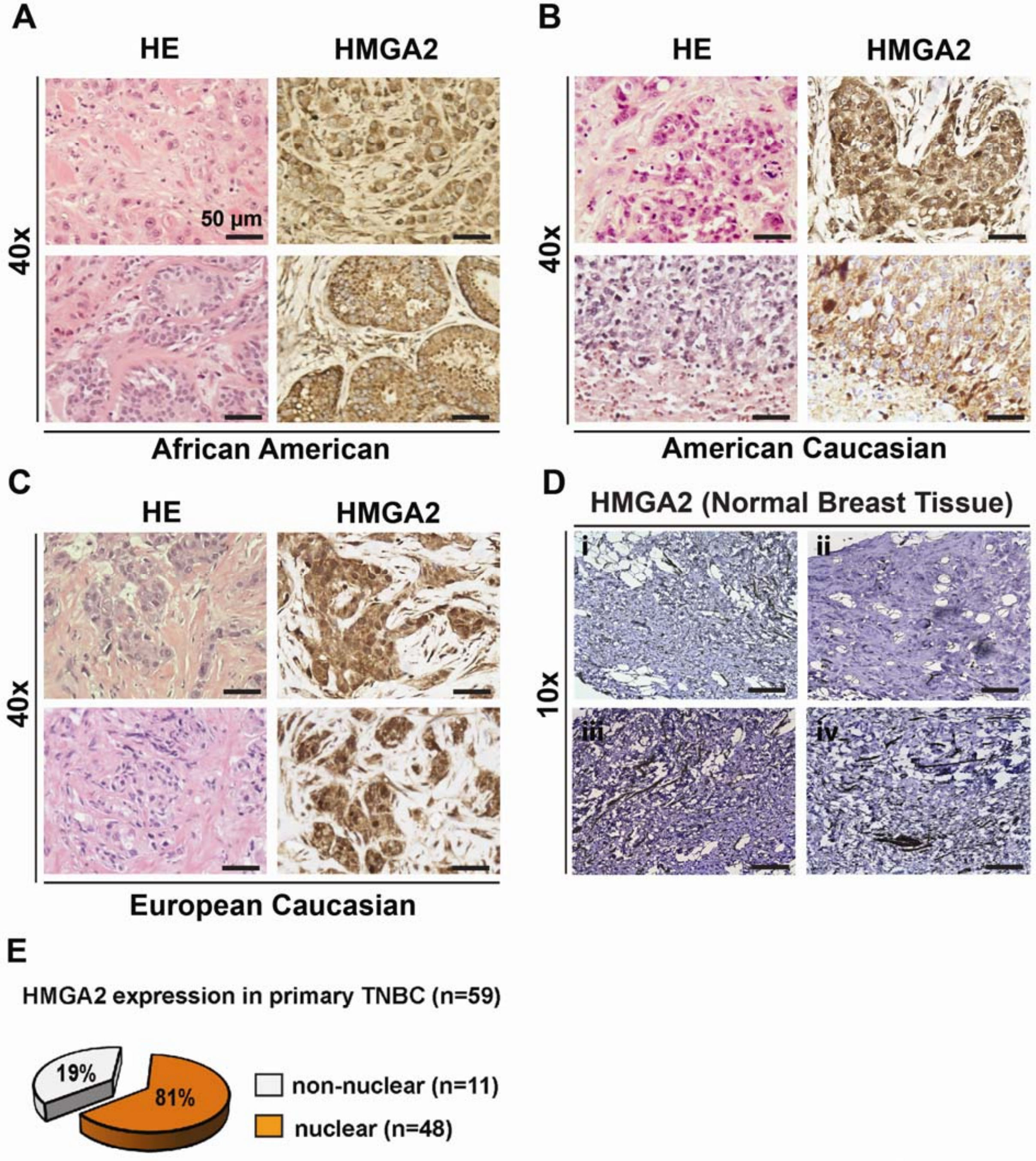

Suppl. Figure 6.
Wend *et al.*

A

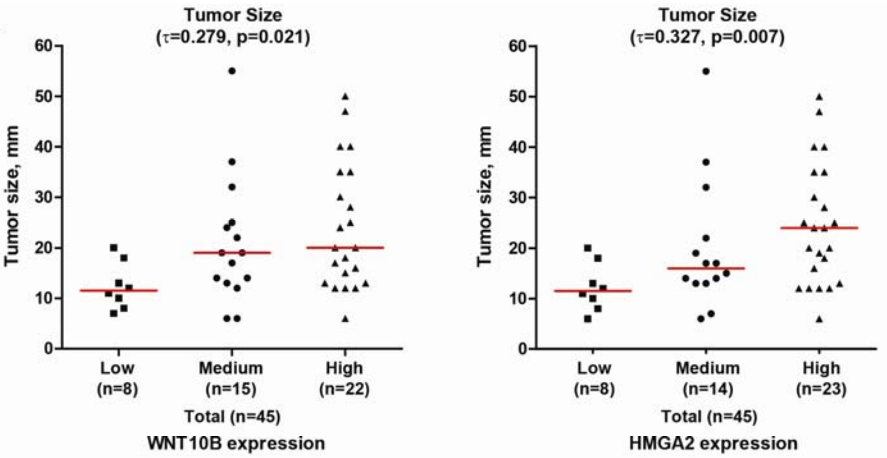

B

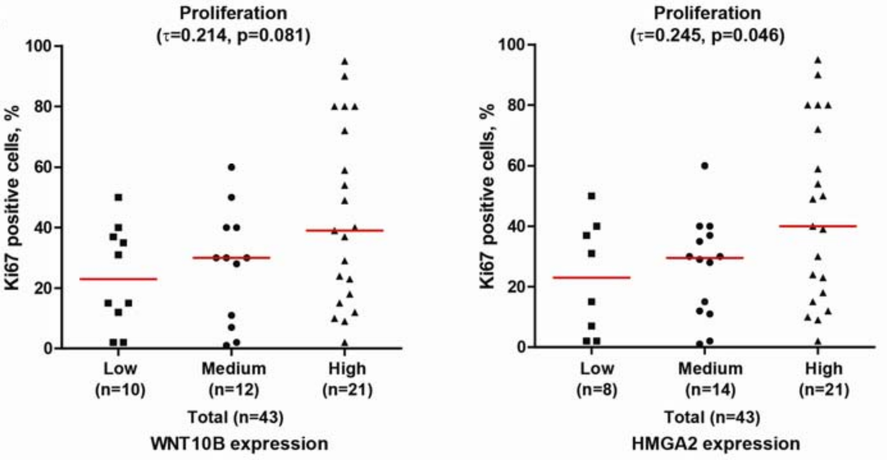

C

Nuclear Grade	WNT10B expression			
	Low	Medium	High	Total
1	1	0	0	1
2	2	2	1	5
3	3	4	13	20
Total (n)	6	6	14	26

Nuclear Grade	HMGA2 expression			
	Low	Medium	High	Total
1	1	0	0	1
2	2	2	1	5
3	3	6	11	20
Total (n)	6	8	12	26

Nuclear Grade		
Statistics	WNT10B	HMGA2
τ	0.420	0.371
p	0.025	0.047
n	26	26

D

Metastasis	WNT10B expression			
	Low	Medium	High	Total
no	8<br>(100%)	11<br>(73.3%)	14<br>(63.6%)	33
yes	0<br>(0%)	4<br>(26.7%)	8<br>(36.7%)	12
Total (n)	8	15	22	45

Metastasis	HMGA2 expression			
	Low	Medium	High	Total
no	8<br>(100%)	11<br>(78.6%)	14<br>(60.9%)	33
yes	0<br>(0%)	3<br>(21.4%)	9<br>(39.1%)	12
Total (n)	8	14	23	45

Metastasis		
Statistics	WNT10B	HMGA2
p	0.076	0.045
n	45	45

Suppl. Figure 7
Wend *et al.*

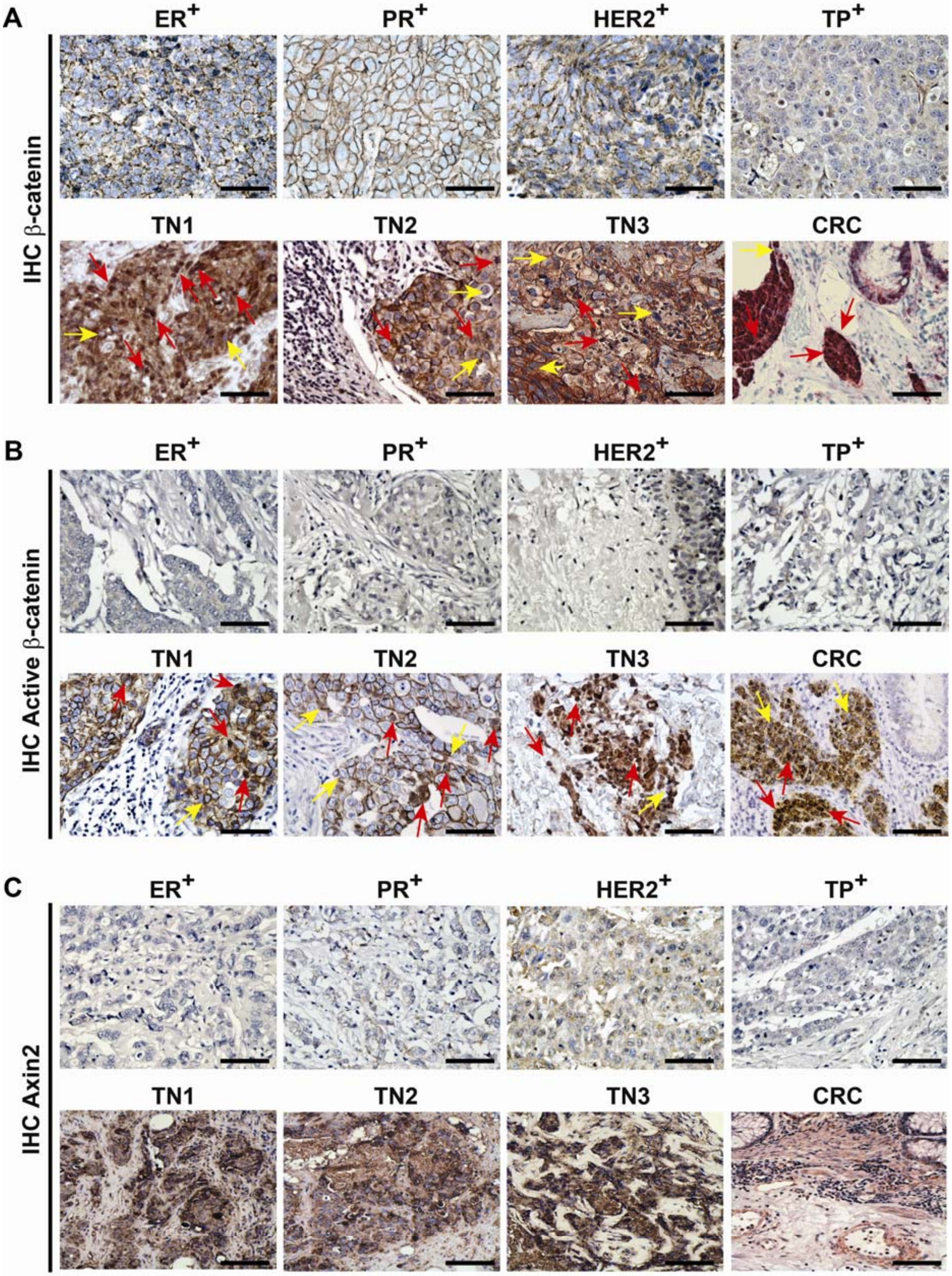

Suppl. Figure 8.
Wend *et al.*

Supplemental Material and Methods:

Human Breast Cancer Tissues: Breast tumor specimens from patients with primary triple-negative breast carcinoma were evaluated. Archival formalin-fixed paraffin-embedded tissues were obtained from the surgical pathology archive of the University of Chicago, USA (H.S.), Cedars-Sinai Medical Center, Los Angeles, USA (S.B.), and Charité University Medicine, Berlin, Germany (C.L.). Tumor staging and grading was performed according to current clinical and pathological classifications. Clinical characteristics of breast cancer patients can be found in Table S1.

Human Tissue Micro Arrays: For immunohistochemistry studies, normal human breast tissue and primary human breast cancer samples from different breast cancer subtypes were obtained from University of Chicago Tissue Bank and from Ohio State University Human Tissue Bank. All specimens were previously stained via Hematoxylin and Eosin and reviewed by study-assigned pathologist's samples.

Cell isolation for FACS analysis: Freshly dissected wildtype mammary glands or mammary tumors from mutant mice were minced and digested for 4 hrs at 37°C in EpiCult-B with 5% fetal bovine serum (FBS), 300 U/ml collagenase and 100 U/ml hyaluronidase (Stem Cell Inc.). Thereafter, epithelial organoids were incubated with 0.25 % trypsin in citrate (Stem Cell Inc.) followed by a treatment with 5 mg/ml dispase II (Stem Cell Inc.) plus 0.1 mg/ml DNase I (Sigma). After lysis of the red blood cells in ice-cold 0.8% NH_4Cl (PBS), cells were washed with staining buffer (1% FBS/PBS) and filtered through a 40 μm mesh. Thereafter, cells were stained for $LacZ^+$/β-galactosidase (FluoReporter lacZ Kit, Molecular Probes) according to the manufacture's protocol. Cells were sorted using FACS Aria (BD Biosciences) and data was analyzed using CELLQuest (BD Biosciences) or FlowJo (Tree Star) softwares. Apoptotic cells were excluded by elimination of DAPI-positive cells.

Cell Culture and Cell proliferation assay: The BTL-10 cell line was established from direct culture of a stage 3 triple-negative breast cancer Caucasian patient and can be obtained from J.L.L. upon request. For the cell proliferation assay cells were seeded at a density of $1x10^3$ cells in 100 μl of medium into each well of 96-well plates and cells were treated accordingly. 10 μl of WST-1 solution was added to each

well, and samples were incubated at 37˚C for 3.5 h. A plate reader was used to detect 414 nm (detection wavelength) and 630 nm (reference wavelength) values, respectively (n=3, test medium served as blank).

Mammosphere formation assay (MSA): MSA cultures were carried according to manufacturer's protocol (Stemcell Technologies). Briefly, $2x10^5$ cells were seeded in ultra-low adherence 6-well culture dishes (Nalge Nunc) and cultured for 10 days in complete Mammocult culture medium (Stem Cell Inc.) containing ICG-001 (10µM) or DMSO (1:1000) as control (each n=3). Lin^-LacZ^+ cells of mouse *Wnt10b*^{LacZ} mammary tumors were seeded at a density of $2x10^3$ cells in 200 µl into each well of ultra-low adherence 96-well plate (Corning) and cultured for 15 days in complete Mammocult culture medium (Stem Cell Inc.) containing ICG-001 (10µM) or DMSO (1:1000) as control. MSA was evaluated using an inverted microscope (Leica DMI6000 B).

Immunohistochemistry (IHC): Tumor tissue and 7-week age-matched whole mammary gland were fixed in 4% paraformaldehyde and embedded in paraffin. A standard deparafinization and staining procedure was used as described (Miranda-Carboni et al, 2008). As primary antibodies were used: WNT10B (ab91201 5A7, Abcam), β-Catenin (C2206, Sigma), Non-phospho (Active) β-Catenin (#8814, Cell Signaling), AXIN2 (ab32197, Abcam), Ki67 (Labvision), Beta-Galactosidase (RGAL-45A-Z, Immunology Consultants Lab), CK5 (PRB-160P, Covance), CK6 (PRB-169P, Covance), HMGA2 (ab52039, Abcam), ERα (MC-20, Santa Cruz), PR (ab2764 PR-AT 4.14, Abcam), and HER2 (OP15, Millipore). Anti-mouse or anti-rabbit secondary antibodies and DAB-based IHC staining solutions were used (K4001, K4003, K3468, DAKO) and counterstaining was conducted with Hematoxylin QS (Vector Labs). ISH was performed as described (Huelsken et al, 2000) and sections were counterstained using nuclear fast red (Vector Labs).

Imaging: Immunohistochemically stained tissue sections were mainly visualized on a Nikon ECLIPSE microscope (Nikon Instruments Inc., USA) using Nikon NIS Elements software. Brightfield microscopic image acquisition of nuclear structures and β-catenin signals was visualized on a Leica DMRE upright microscope (Leica Microsystems, Wetzlar, Germany) equipped with a water immersion objective (HCX APO 63x/0.90 NA) and a Nuance EX flexible bandwidth multispectral imaging system (CRi/Caliper Life

Sciences, Woburn, MA). Images were captured automatically by 10 nm increments from 450 nm to 750 nm under constant light illumination. The resulting spectral imaging data set was unmixed and analyzed with the vendor's software allowing spectral characterization for each of the multi-labeled components in the image.

Western blotting: Cells were lysed as previously described (Miranda-Carboni et al, 2008) 50-250μg of protein were loaded per lane and separated by SDS-PAGE 10% gels. After transfer, Immobilon-P (Millipore) was immunoblotted using the following primary antibodies: HMGA2 (#5269, Cell Signaling), PCNA (#2586, Cell Signaling), Cyclin A2 (E23.1, sc-53228, Santa Cruz), Cyclin B1 (H-433, sc-752, Santa Cruz), Cyclin E1 (M-20, sc-481, Santa Cruz), Cdk2 (D-12, sc-6248, Santa Cruz), Cdk4 (C-22, sc-260, Santa Cruz), E2F-1 (C-20, sc-193, Santa Cruz), p-Rb (Ser807/811, sc-807/811, Santa Cruz), Rb (C-15, sc-50, Santa Cruz), β-Catenin (H-102, sc-7199, Santa Cruz), CBP (A-22, sc-369, Santa Cruz), β-actin (20-33 A5060, Sigma). ImmunoPure-peroxidase conjugated secondary antibodies (Thermo Scientific) were used according to manufacturer's protocols.

Chromatin immunoprecipitation (ChIP). ChIP was performed as previously described (Krum et al, 2008a; Krum et al, 2008b) Briefly, cross-linked chromatin was isolated from each plate and separate precipitation reactions were set up for each target protein complex using specific antisera. DNA was amplified by QT-PCR using the Applied Biosystems Power SYBR Green amplification system. Immunoprecipitated chromatin was amplified in triplicate for each target gene and run on an iCycler thermocycler, as described above. Normalization was conducted to input chromatin, and to the *mGapdh* or to *hHBB* minimal promoters. Antibodies used included RNA pol II (#17-672, Millipore) and β-catenin (sc-7199 H-102, Santa Cruz). Each experiment was repeated at least 3 times. Primer pairs for each gene are provided in Supplemental Table S4.

RNA and Real-Time PCR: Isolation of total RNA was performed using TRIzol (Invitrogen) according to manufacturer’s protocol. RNA was treated with DNA-free kit (Ambion) and converted to cDNA with iScript cDNA Synthesis Kit (BioRad) or Maxima First Strand cDNA Synthesis Kit (Fermentas) according to manufacturer’s protocol**.** cDNA was subjected to quantitative PCR (qt-PCR) using the

iCycler thermocycler (BioRad) or Realplex2 cycler (Eppendorf). qt-PCR was conducted in a final volume of 20 µl using Maxima SYBR Green/ROX qPCR Master Mix (Fermentas) according to manufacturer's protocols. Amplification conditions were: 95°C (5'), 40 cycles of 95°C (30s), 55°C (60s) and 72°C (60s). Primer pairs for each gene are provided in Supplemental Table S3.

Reporter Gene Assay: A GFP-based reporter assay was used to measure transcriptional activity of Wnt/βcatenin signaling. For TCF/LEF-mediated transcription, a Cignal™ Lenti TCF/LEF Reporter (GFP) reporter assay kit (Cat# CLS-018G, SABiosciences) was used according to the manufacturer's protocol. GFP signal was measured by immunofluorescence microscopy.

Affymetrix Microarrays. The samples were processed by UCLA's DNA Microarray Core Facility (http://www.genetics.ucla.edu/microarray/). The procedures for probe preparation, hybridization, washing, scanning and signal intensity normalization was by manufactures protocols using GeneChip Operating Software (GCOS) v1.1.1 Affymetrix (Santa Clara, CA). Analysis was conducted on the Affymetrix GeneChip Mouse Genome 430 2.0 arrays in triplicates for virgin and pregnant tissue and/or wnt10b/erbB2 derived tumors.

Bioinformatics. MAIME structure http://www.mged.org. Using GeneSpring GX 7.3 software (Agilent Corp.) the log of ratio normalized expression data was analyzed with cross-gene error model turned on and normalized per manufacture protocol. Calculations without the assumption of equality of variances were done using Welch's approximate t test and 1-way-ANOVA, with p value cutoff of 0.05. Benjamini and Hochberg false discovery rate was used. Hierarchical clustering analysis was performed the average-linkage method (Eisen, 1998 #208)**.** Additional hypothesis-driven analyses were conducted using a conjoint Boolean consistency so noise ratio could be decreased and looking at gene expression 1.6 fold or greater over virgin samples.

Table S1: Clinical Characteristics of Breast Cancer Patients[1]

No	Origin	Age at Diagnosis	Tumor size, mm	Ki67 %	Nuclear grade	Metastasis (1-yes, 0-no)	WNT10B intensity[2]	HMGA2 intensity[2]
1	CS LA[3]	78	14	2%	2	0	Medium	Medium
2	CS LA	37	22	30%	3	1	Medium	Medium
3	CS LA	43	20	54%	3	0	High	High
4	CS LA	45	24	15%	2	0	High	High
5	CS LA	33	47	72%	3	1	High	High
6	CS LA	71	6	7%	3	0	Medium	Low
7	CS LA	66	12	23%	3	1	High	High
8	CS LA	35	12	49%	3	0	High	High
9	CS LA	63	13	29%	3	0	High	Medium
10	CS LA	79	18	80%	3	0	High	High
11	CS LA	84	20	50%	3	0	Low	Low
12	CS LA	80	35	9%	3	0	High	High
13	CS LA	70	13	39%	3	1	High	High
14	CS LA	56	25	59%	na	1	High	High
15	CS LA	44	12	12%	3	1	High	High
16	CS LA	58	8	2%	2	0	Low	Low
17	CS LA	70	6	1%	2	0	Medium	Medium
18	CS LA	43	12	30%	na	0	Medium	High
19	CS LA	46	10	31%	3	0	Low	Low
20	CS LA	45	7	12%	3	0	Low	Medium
21	CS LA	68	50	10%	3	0	High	High
22	CS LA	79	19	28%	3	1	Medium	Medium
23	CS LA	51	12	37%	2	0	Low	Low
24	CS LA	53	11	2%	1	0	Low	Low
25	CS LA	45	15	37%	3	0	High	Medium
26	CS LA	54	40	18%	na	1	High	High
27	CS LA	53	30	24%	3	1	High	High
28	CS LA	52	20	2%	3	0	High	High
29	CS LA	49	55	11%	3	0	Medium	Medium
30	CS LA	47	6	na	na	0	High	High
31	CS LA	37	14	na	na	0	Medium	Medium
32	U Chicago[4]	62	18	na	na	0	Low	Low
33	U Chicago	86	13	na	na	0	Low	Low
34	U Chicago	71	25	na	na	0	Medium	High
35	U Chicago	43	37	na	na	0	Medium	Medium
36	U Chicago	60	17	na	na	0	High	Medium
37	U Chicago	83	32	na	na	0	Medium	Medium
38	U Chicago	71	13	na	na	1	Medium	Medium
39	U Chicago	66	17	na	na	0	Medium	Medium
40	U Chicago	84	19	na	na	0	Medium	High
41	U Chicago	64	24	na	na	1	Medium	High
42	U Chicago	63	40	na	na	0	High	High
43	U Chicago	51	35	na	na	1	High	High
44	U Chicago	70	28	na	na	0	High	High
45	U Chicago	55	16	na	na	0	High	High
46	Ch Berlin[5]	50	na	50%	na	na	Medium	High

47	Ch Berlin	58	na	90%	na	na	High	High
48	Ch Berlin	55	na	40%	na	na	High	High
49	Ch Berlin	48	na	40%	na	na	Medium	Low
50	Ch Berlin	71	na	95%	na	na	High	High
51	Ch Berlin	51	na	35%	na	na	Low	Medium
52	Ch Berlin	87	na	15%	na	na	Low	Low
53	Ch Berlin	38	na	60%	na	na	Medium	Medium
54	Ch Berlin	92	na	30%	na	na	Medium	Medium
55	Ch Berlin	76	na	40%	na	na	Medium	Medium
56	Ch Berlin	44	na	80%	na	na	High	High
57	Ch Berlin	68	na	15%	na	na	Low	Medium
58	Ch Berlin	69	na	40%	na	na	Low	Medium
59	Ch Berlin	60	na	80%	na	na	High	High

[1]Tumor specimens from 59 female patients with grade 3 primary triple-negative breast tumors (i.e., negative for ER, PR, Her2) were evaluated. Tumor staging and grading was performed according to current clinical and pathological classifications.
[2]Scoring was based on intensity and percentage of positively stained cells for WNT10B and HMGA2 by immunohistochemistry, Low; 1-30%, Medium; 30-70%, High; ≥70%
[3]Dept. of Pathology and Laboratory Medicine, Cedars Sinai Medical Center, Los Angeles, USA
[4]Dept. of Pathology, The University of Chicago, Chicago, IL 60637, USA
[5]Institute of Pathology, Charité University Medicine/UKBF, 12200 Berlin, Germany

Table S2: Oligonucleotide sequences for transient RNAi:

Gene	Oligo No.	Target Sequence
CTNNB1	1	5’-GCUGAAACAUGCAGUUGUAUU-3′
	2	5’-GAUAAAGGCUACUGUUGGAUU-3′
	3	5’-CCACUAAUGUCCAGCGUUUUU-3′
	4	5’-ACAAGUAGCUGAUAUUGAUUU-3′
Luciferase	1	5’-CACUUACGCUGAGUACUUCGAdTdT-
	2	5’-UCGAAGUACUCAGCGUAAGUGdTdT-

Table S3. Primer sequences used for qt-PCR:

Primer	Sequence	Orientation
hAXIN2	5’-TCA AGT GCA AAC TTT CGC CAA CCG-3’	S
	5’-TGG TGC AAA GAC ATA GCC AGA ACC-3’	AS
hβ-actin	5’GGACTTCGAGCAAGAGATGG-3’	S
	5’-AGC ACT GTG TTG GCG TAC AG-3’	AS
mβ-actin	5’-AGC CAT GTA CGT AGC CAT CC-3’	S

	5'-CTC TCA GCT GTG GTG GTG AA-3'	AS
hBIRC5	5'-CCG CAT CTC TAC ATT CAA GAA CTG GC-	S
	5'-TTG ACA GAA AGG AAA GCG CAA CCG-3'	AS
hBMI1	5'-TGC CTA AAA GCG GGT ACT ACC-3'	S
	5'TGC AAA GGT CGA ACC AGT TGG GA-3'	AS
hc-myc	5'-TCT CCA CAC ATC AGC ACA ACT ACG-3'	S
	5'-TGT GTT CGC CTC TTG ACA TTC TCC-3'	AS
mc-myc	5'-TCC TGA AGC AGA TCA GCA ACA ACC-3'	S
	5'-TGC TTG AAT GGA CAG GAT GTA GGC-3'	AS
hCCNA1	5'-TGT CTG TTC TGA GAG GGA AAC TGC-3'	S
	5'-AAG GAG AAA CTG GTT GGT GGT TGG-3'	AS
hCCNA2	5'-CCA ATA CTT TCT GCA TCA GCA GCC-3'	S
	5'-AAT GAT TCA GGC CAG CTT TGT CCC-3'	AS
hCCNB1	5'-ATT GTG TGC CCA AGA AGA TGC TGC-3'	S
	5'-TTA GAT GCT CTC CGA AGG AAG TGC-3'	AS
hCCNB2	5'-TTT ACA GGT TCA GCC AGT TTC CCG-3'	S
	5'-TGC TCG CCT TAA GAA GTG TAG TGG-3'	AS
hCCND1	5'-AAG TTC ATT TCC AAC CCA CCC TCC-3'	S
	5'-AGA AGG GCT TCA ATC TGT TCC TGG-3'	AS
mCcnd1	5'-GAC TGC CGA GAA GTT GTG CAT-3'	S
	5'-GTT CAC CAG AAG CAG TTC CAT TT-3'	AS
hCCNE1	5'-TTA CCC AAA CTC AAC GTG CAA GCC-3'	S
	5'-AGA GGG TGT TGC TCA AGA AAG TGC-3'	AS
hCCNE2	5'-TGA GGT CCA TAC TTC TAG ACT GGC-3'	S
	5'-GAT ATC CTC TTC ACT GCA AGC ACC-3'	AS
hCTNNA1	5'-AGC TTG TTC GAA TGT CTG CAA GCC-3'	S
	5'-ATC GAC AGC ATC TGT GAG AAC ACG-3'	AS
hCTNNB1	5'-TTC GAA ATC TTG CCC TTT GTC CCG-3'	S
	5'-AAT TCG GTT GTG AAC ATC CCG AGC-3'	AS
hDKK1	5'-TGT TGT GCT AGA CAC TTC TGG TCC-3'	S
	5'-TTT CTG TAT CCG GCA AGA CAG ACC-3'	AS
mEr-α	5'-GCCAAGGAGACTCGCTACTG-3'	S
	5'-CTCCGGTTCTTGTCAATGGT-3'	AS
mErbb2	5'-GTTGCTCCCCTGGCCTGCAG-3'	S
	5'-GGCAGTGCCTGCTCTGGGTG-3'	AS
hGAPDH	5'-AAC AGC GAC ACC CAT CCT C-3'	S
	5'-CAT ACC AGG AAA TGA GCT TGA	AS
mGapdh	5'-GGGAAGCCCATCACCATCTT-3'	S
	5'-ACATACTCAGCACCGGCCTC-3'	AS
hHMGA1	5'-GGG GCC GAC CAA AGG GAA GC-3'	S
	5'-GGC ACG CAT GGG TCA CTG CT-3'	AS
mHmga1	5'-GGG GCC GAC CAA AGG GAA GC-3'	S
	5'-GGG GCC GAC CAA AGG GAA GC-3'	AS
hHMGA2	5'-GCC CCA GGA AGC AGC AGC AA-3'	S

	5’-TCG AAC GTT GGC GCC CCC TA-3’	AS
mHmga2	5’-CCG CTG GAC GTC CGG TGT G-3’	S
	5’-CGC CCA GCA CCT TTC GGG AG-3’	AS
hLRP6	5’-AAA TCG GCA GGC AGT GGT TAA AGG-3’	S
	5’-CAT GGA TTT GTG GCA TTT GGC TGC-3’	AS
	5’-ACT GCC AGA TGG ATT GGA AGT GC-3’	AS
hPS2	5’-TTGTGGTTTTCCTGGTGTCA-3’	S
	5’-CCGAGCTCTGGGACTAATCA-3’	AS
mPr	5’-GGT GGA GGT CGT ACA AGC AT-3’	S
	5’-CTC ATG GGT CAC CTG GAG TT-3’	AS
hWNT1	5’-ACG GCG TTT ATC TTC GCT ATC ACC-3’	S
	5’-GTT GTG AAG GTT CAT GAG GAA GCG-3’	AS
hWNT10B	5’-TGG GAT GTG TAG CCT TCT CC-3’	S
	5’-CCC AGC CAA AAG GAG TAT GA-3’	AS
mWnt10b	5’-ATG CGG ATC CAC AAC AAC AG-3’	S
	5’-TGA CGT TCC ATG GCA TTT G-3’	AS
hXBP1	5’-TTGTGGTTTTCCTGGTGTCA-3’	S
	5’-CCGAGCTCTGGGACTAATCA-3’	AS

Legend: Antisense (AS), Sense (S), human (h) and mouse (m)

Table S4. Primer sequences used for qt-PCR of ChIP samples

**Primer**	**Sequence**	**Orientation**
mHmga2 promoter	5’-TCCACCGAGGGTTGCCCGAA-3’	S
	5’-GGTCGCTCTTTTCCCGGGGC-3’	AS
mHmga2 -6kB prom	5’-TTCCATCTCCTTACAGATGGTGGC-3’	S
	5’-GTCTTGCCAGGAGGAATAATGTGC-3’	AS
mMyc promoter	5’-TGCCCAGTCAACATAACTGTACGACC-3’	S
	5’-AGAGCCACTTAGGGATAAACAGCC-3’	AS
mGapdh promoter	5’-ACGGCGGTTCATTCATTTCCTTCC-3’	S
	5’-TGCATACCTTTGCGCATCATCTCC-3’	AS
hHMGA2 promoter	5’-ACTTGGCAGAAAGAGAGTTCTCAGGC-3’	S
	5’-TTCTCCAGGAAAGACTAGAGGCAACC-3’	AS
hHBB promoter	5’-TGGTATGGGGCCAAGAGATA-3’	S
	5’-TAGATGGCTCTGCCCTGACT-3’	AS

Legend: Antisense (AS), Sense (S), human (h) and mouse (m)
